# Risk stratification of ibrutinib toxicity co-administered with triazole antifungals: insights from real-world pharmacovigilance and clinical evidence

**DOI:** 10.3389/fmed.2026.1875804

**Published:** 2026-08-31

**Authors:** Shiyun Li, Zijuan Li, Shiqiao Wang

**Affiliations:** 1 Department of Pharmacy, The Affiliated Guangdong Second Provincial General Hospital of Jinan University, Guangzhou, China; 2 College of Pharmacy, Guangdong Pharmaceutical University, Guangzhou, China

**Keywords:** adverse events, FAERS database, Ibrutinib, pharmacovigilance, triazole antifungal agents

## Abstract

**Objective:**

The co-administration of ibrutinib and triazole antifungals results in known pharmacokinetic interactions, but the comprehensive clinical risk profile and grading characteristics remain underexplored. This study integrated real-world pharmacovigilance data, clinical pharmacokinetic studies, and published clinical cases to evaluate the comparative safety profiles and risk gradients of these drug combinations.

**Methods:**

Adverse drug events (ADEs) from the FDA Adverse Event Reporting System (FAERS) between Q1 2013 and Q2 2025 were analyzed. Disproportionality analyses utilizing the reporting odds ratio (ROR) and proportional reporting ratio (PRR) were employed to detect event reporting signals across ibrutinib without triazole, triazole without ibrutinib, and combination therapy groups. The time-to-onset (TTO) was assessed via Weibull distribution analysis. A systematic review of 18 individual patient cases reported in 11 publications and relevant pharmacokinetic data supported clinical interpretation.

**Results:**

Among the submitted reports, serious outcomes accounted for 66.95% of those involving combination therapy, compared with 46.38% for ibrutinib without triazole and 46.85% for triazole without ibrutinib, respectively. Strong cytochrome P450 3A (CYP3A) inhibitors increased ibrutinib exposure by 5.7– to 10.3-fold, whereas isavuconazole increased exposure by approximately 2.1-fold. Voriconazole-containing reports showed a signal for cerebral aspergillosis (ROR = 474.61, *n* = 3), posaconazole-containing reports showed cardiac events including pericardial effusion (ROR = 24.95, *n* = 4), and isavuconazole-containing reports showed a signal for febrile neutropenia (ROR = 29.7, *n* = 4). For combination therapy reports with analyzable date information (157/590, 26.6%), the median reported onset was 63 days.

**Conclusions:**

The identified pharmacovigilance signals are clinically interpretable in light of known CYP3A-mediated pharmacokinetic interactions, but they should be considered hypothesis-generating rather than causal. These findings support clinical awareness, individualized medication review, and further validation in studies with defined denominators and appropriate control of confounding.

## 1 Introduction

On November 13, 2013, the FDA granted approval for the use of ibrutinib, a Bruton's Tyrosine Kinase (BTK) Inhibitor, in patients with mantle cell lymphoma (MCL) who had undergone at least one previous treatment (1). Ibrutinib has substantially increased survival rates for people with chronic lymphocytic leukemia (CLL)/small lymphocytic lymphoma (SLL) (2), mantle cell lymphoma (MCL) (3), and Waldenström's macroglobulinemia (WM) (4). Ibrutinib is the standard treatment for lymphoma (5–7).

Patients with lymphoma are more susceptible to invasive fungal infections (IFIs) due to their immunocompromised state (8). A single-center retrospective study identified *Aspergillus species* as the causative agent in 27 out of 33 patients (81%) with IFIs who received ibrutinib (9). Triazole antifungal agents are the preferred therapy for *Aspergillus* infections. However, triazole antifungal agents are potent or moderate CYP3A4 inhibitors, which is responsible for the metabolism of ibrutinib. Pharmacological interactions may lead to adverse reactions, which may require discontinuation of treatment. Currently, there has been no systematic comparison of the risk profiles across different triazole antifungals, particularly the newer agent isavuconazole. Most existing evidence is derived from theoretical assumptions or isolated case reports potentially overlooking critical safety signals.

To address these gaps, this study conducted a comprehensive assessment that combined pharmacovigilance analysis based on the U.S. Food and Drug Administration Adverse Event Reporting System (FAERS) with a systematic review of published clinical cases and pharmacokinetic evidence. The study primarily aims to quantify and compare the clinical safety profiles of different triazole antifungal agents, describe the temporal patterns of associated events, and offer clinically actionable insights to inform treatment decisions.

## 2 Methods

### 2.1 Analysis of the FAERS database

FAERS is a publicly available database of drug safety reports submitted by healthcare professionals, companies, and consumers (10). Reports are given a unique code to link the datasets. ADEs were grouped and coded using the Preferred Terms (PTs) in MedDRA version 27.1 [International Council for Harmonisation of Technical Requirements for Pharmaceuticals for Human Use (ICH)] (11). It followed the READUS-PV guideline (12).

We downloaded datasets from the FAERS database covering the period from the first quarter of 2013 to the second quarter of 2025, including data from DEMO, DRUG, REAC, OUTC, and THER, and imported them into R version 4.3.2 (Open Source Software, University of Auckland, New Zealand) for data cleaning. In this work, both generic name and brand name of ibrutinib and each triazole antifungal were utilized to query the database (The details of generic names and brand names are listed in Supplementary Table S1). Given potential duplicate entries in the database, we employed the deduplication principles recommended by the FDA to remove relevant duplicate reports: selecting the most recent FDA_DT in cases when the CASE IDs and FDA_DT (reported data) are identical, and selecting the most recent PRIMARY ID (reported record ID) in those cases (13).

**Figure 1:**
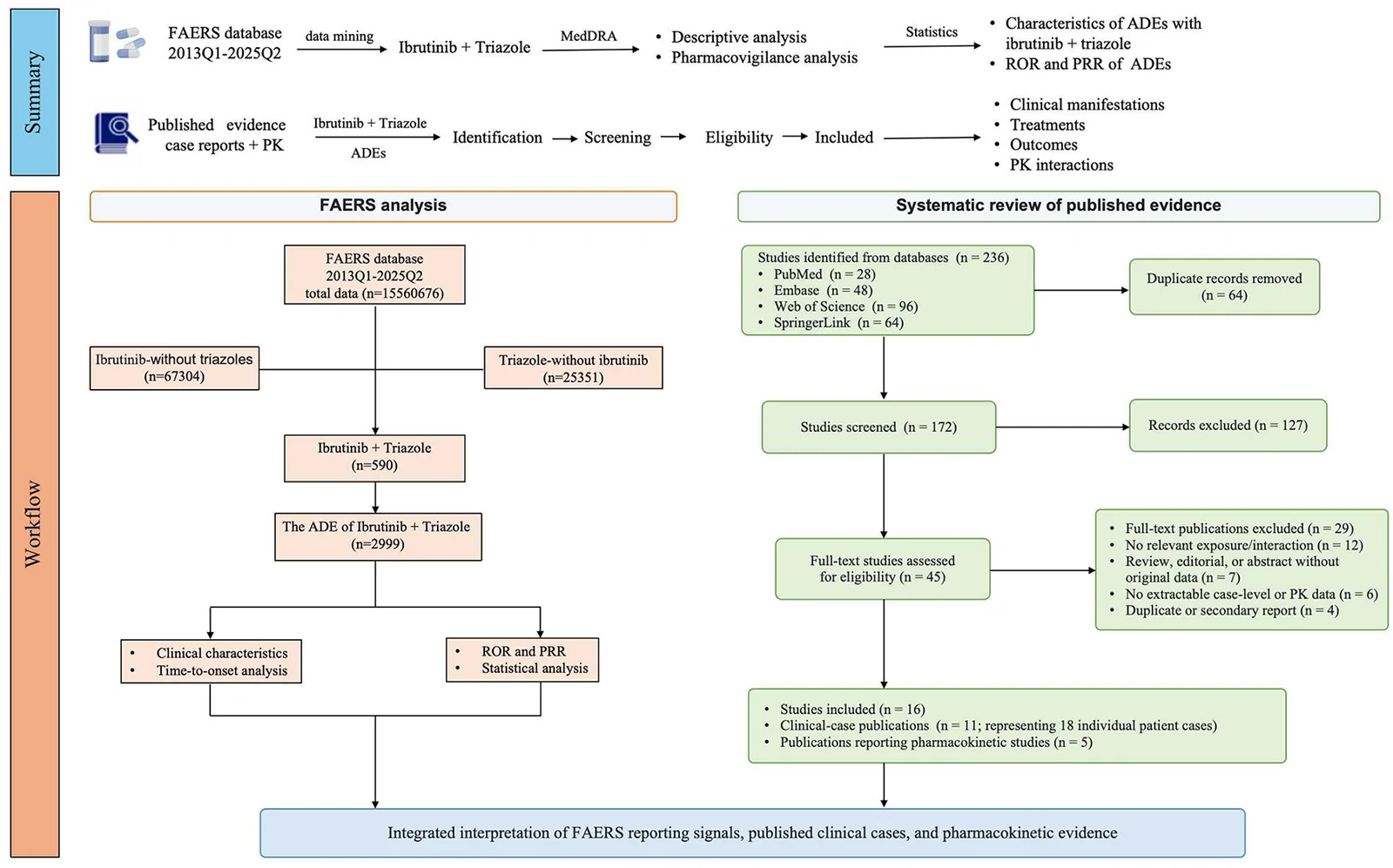
Flow chart showing the analysis process of the study.

In the FAERS database, drug role codes include Primary Suspect (PS), Secondary Suspect (SS), Concomitant (C), and Interacting (I) (10). In this study, the inclusion criteria for the drug data were defined as follows: the ibrutinib without triazole group included reports in which ibrutinib was comprised as the primary suspect drug (PS), with triazole antifungal agents excluded as secondary suspect drugs (SS), concomitant medications (C), or interacting drugs (I). The triazole without ibrutinib group comprised reports in which triazole antifungals were the primary suspect drug (PS), and ibrutinib was not present as SS, C, or I. The ibrutinib-plus-triazole group consisted of reports in which both drugs were present. Either ibrutinib was the PS and triazole was an SS, C or I, or triazole was the PS and ibrutinib was an SS, C or I. These exposure-group labels indicate exclusion of the paired target drug only and do not imply the absence of other concomitant medications. Figure 1 illustrates the screening process.

### 2.2 Systematic literature review

The study selection process was performed independently by two investigators, according to the PRISMA statement (14). Duplicate records were removed using EndNote X8 (Clarivate Analytics, Philadelphia, PA, USA) and then manually checked by DOI, PMID, title, first author, and publication year. Titles and abstracts were screened for relevance, and the full texts of potentially eligible articles were retrieved and assessed against the pre-specified inclusion and exclusion criteria. Disagreements were resolved by discussion

The literature component was conducted as a systematic case review of published clinical cases and pharmacokinetic or drug-interaction evidence, with reference to methodological guidance for systematic reviews (15). PubMed, Embase, Web of Science, and Springer Link were searched from database inception to November 3, 2025. Search strategies are presented in Supplementary Table S2. No language restrictions were initially applied.

Studies were included if they specifically involved the concomitant administration of ibrutinib and any of the five triazole antifungals in humans. The principal exclusion criterion was the absence of confirmed co-administration of both drugs. For pharmacokinetic studies, inclusion required the report of quantitative changes in ibrutinib's pharmacokinetic parameters, with the study design and data interpretation referenced against established clinical drug interaction guidance. For case reports, inclusion criteria require a detailed description of changes in the clinical condition of patients receiving combination therapy. Reviews, editorials, *in-vitro* studies, and conference abstracts with insufficient data were excluded. Clinical-case publications and pharmacokinetic or drug-interaction studies were screened and summarized separately. For the literature component, data were extracted at the individual patient case level. Publications reporting multiple eligible patients were counted once at the publication level but contributed multiple individual cases when patient-level information was available. Pharmacokinetic or drug-interaction studies were summarized separately and were not counted as clinical cases. The database-specific counts, deduplication and PRISMA accounting were illustrated in Figure 1.

### 2.3 Data mining and analysis

Disproportionality and sensitivity analyzes employed two algorithms: the Reporting Odds Ratio (ROR) and the Proportional Reporting Ratio (PRR). The confidence interval (CI) indicates the range within which the true mean of the sample is likely to be found (16). ROR and PRR values were derived from a 2 × 2 contingency table (Supplementary Table S3). According to Supplementary Table S4, the ROR was considered significant if the lower bound of the 95% CI exceeded one, and the PRR was considered significant if it was at least two and the chi-squared (χ^2^) value was at least four (16).

The time-to-onset (TTO) of ADEs was defined as the interval from the start of treatment to the occurrence of an ADE (17). TTO was described using the median and interquartile range (IQR), which shows the spread from the first to the third quartiles (11). Records with missing or incorrectly formatted values were excluded from the TTO and descriptive analyzes. The reporting frequency of drug adverse reactions over time was evaluated and predicted using the Weibull distribution (18). This parametric distribution is jointly determined by the scale parameter α and the shape parameter β*;* if β is less than one, the reporting frequency decreases over time, indicating early failure.

Data extraction and analysis were performed using R version 4.3.2 (Open Source Software, University of Auckland, New Zealand), Microsoft Excel 2019 (Microsoft Corporation, Redmond, WA, USA), and Origin 2021 (OriginLab Corporation, Northampton, MA, USA).

## 3 Results

### 3.1 Clinical baseline characteristics and ADE trends

Demographic and baseline characteristics were analyzed descriptively using FAERS data from Q1 2013 to Q2 2025. ADE reports were evaluated for the ibrutinib without triazole group (*n* = 67,304), triazole without ibrutinib group (*n* = 25,351), and combination therapy group (*n* = 590) as detailed in Table 1. The combination therapy group had the highest proportion of male patients (62.4%), followed by ibrutinib without triazole (57.2%) and triazole without ibrutinib (47.3%). Patients aged over 64 years accounted for 39.3% of the combination therapy group and 35.2% of the ibrutinib without triazole group, while the triazole without ibrutinib group had a higher proportion of patients aged 18–64 (39.5%) and 8.2% under 18. The combination therapy group was higher in reporting proportion of severe ADEs compared to the ibrutinib without triazole group and triazole without ibrutinib group. Severe outcomes, including death, life-threatening events, hospitalization, and disability, occurred in 66.95% of the combination group, 46.38% of the ibrutinib without triazole group, and 46.85% of the triazole without ibrutinib group. Reports from the Americas were most frequent in the combination therapy group (62.5%). Consumer submissions made up the majority of reports in the ibrutinib without triazole (67.7%) and combination therapy (47.5%) groups, while the triazole without ibrutinib group had a higher proportion of reports from physicians and other professionals. Ibrutinib without triazole group reports grew after its 2014 launch, reaching a peak in 2022. Triazole reports remained steady. Combination therapy reports began in 2014, increased until 2018, then declined, but continued annually through mid-2025.

**Table 1:** Demographic characteristics of patients and composition of adverse drug events.

Characteristics		Report number, *N* (%)		
		Ibrutinib without triazole	Triazole without ibrutinib	Combination therapy
**Number of reports**		**67,304**	**25,351**	**590**
Gender	Female	24,532 (36.45)	10,032 (39.57)	201 (34.07)
	Male	38,502 (57.21)	11,981 (47.26)	368 (62.37)
	Unknown or missing	4,270 (6.34)	3,338 (13.17)	21 (3.56)
Age (years)	< 18	371 (0.55)	2,071 (8.17)	15 (2.54)
	18 ≤ and ≤ 64	6,903 (10.26)	10,002 (39.45)	163 (27.63)
	>64	23,703 (35.22)	6,604 (26.05)	232 (39.32)
	Unknown or missing	36,327 (53.97)	6,274 (24.75)	180 (30.51)
Serious outcome	Death	11,293 (16.78)	4,725 (18.64)	121 (20.51)
	Life-threatening	890 (1.32)	1,179 (4.65)	25 (4.24)
	Hospitalization	18,883 (28.06)	5,680 (22.41)	247 (41.86)
	Disability	152 (0.23)	293 (1.16)	2 (0.34)
	Others	36,086 (53.62)	13,474 (53.15)	195 (33.05)
Reported countries (Top five)	Americas	49,269 (73.2)	10,443 (41.19)	369 (62.54)
	Canada	1,778 (2.64)	571 (2.25)	8 (1.36)
	France	1,405 (2.09)	2,469 (9.74)	18 (3.05)
	Colombia	1,267(1.88)	/	10 (1.69)
	Germany	978(1.45)	571(2.25)	11 (1.86)
Reported person	Physician	11,346(16.86)	6,946(27.4)	203 (34.41)
	Pharmacist	1,521(2.26)	2,334(9.21)	18 (3.05)
	Other health-professional	5,636(8.37)	5,393(21.27)	41 (6.95)
	Consumer	45,564 (67.7)	5,897 (23.26)	280 (47.46)
	Unknown	3,237 (4.81)	4,781 (18.86)	48 (8.14)
Reporting year	2013	10 (0.01)	231 (0.91)	/
	2014	932 (1.38)	1,147 (4.52)	23 (3.9)
	2015	3,293 (4.89)	1,709 (6.74)	42 (7.12)
	2016	3,673 (5.46)	1,890 (7.46)	51 (8.64)
	2017	4,656 (6.92)	2,514 (9.92)	63 (10.68)
	2018	6,148 (9.13)	2,455 (9.68)	95 (16.1)
	2019	7,563 (11.24)	2,741 (10.81)	71 (12.03)
	2020	9,604 (14.27)	2,435 (9.61)	76 (12.88)
	2021	6,832 (10.15)	2,116 (8.35)	48 (8.14)
	2022	10,996 (16.34)	2,162 (8.53)	41 (6.95)
	2023	7,779 (11.56)	2,216 (8.74)	39 (6.61)
	2024	4,078 (6.06)	2,390 (9.43)	28 (4.75)
	2025Q2	1,740 (2.59)	1,345 (5.31)	13 (2.2)

### 3.2 Statistical analysis of ADEs across different treatment groups

**Figure 2:**
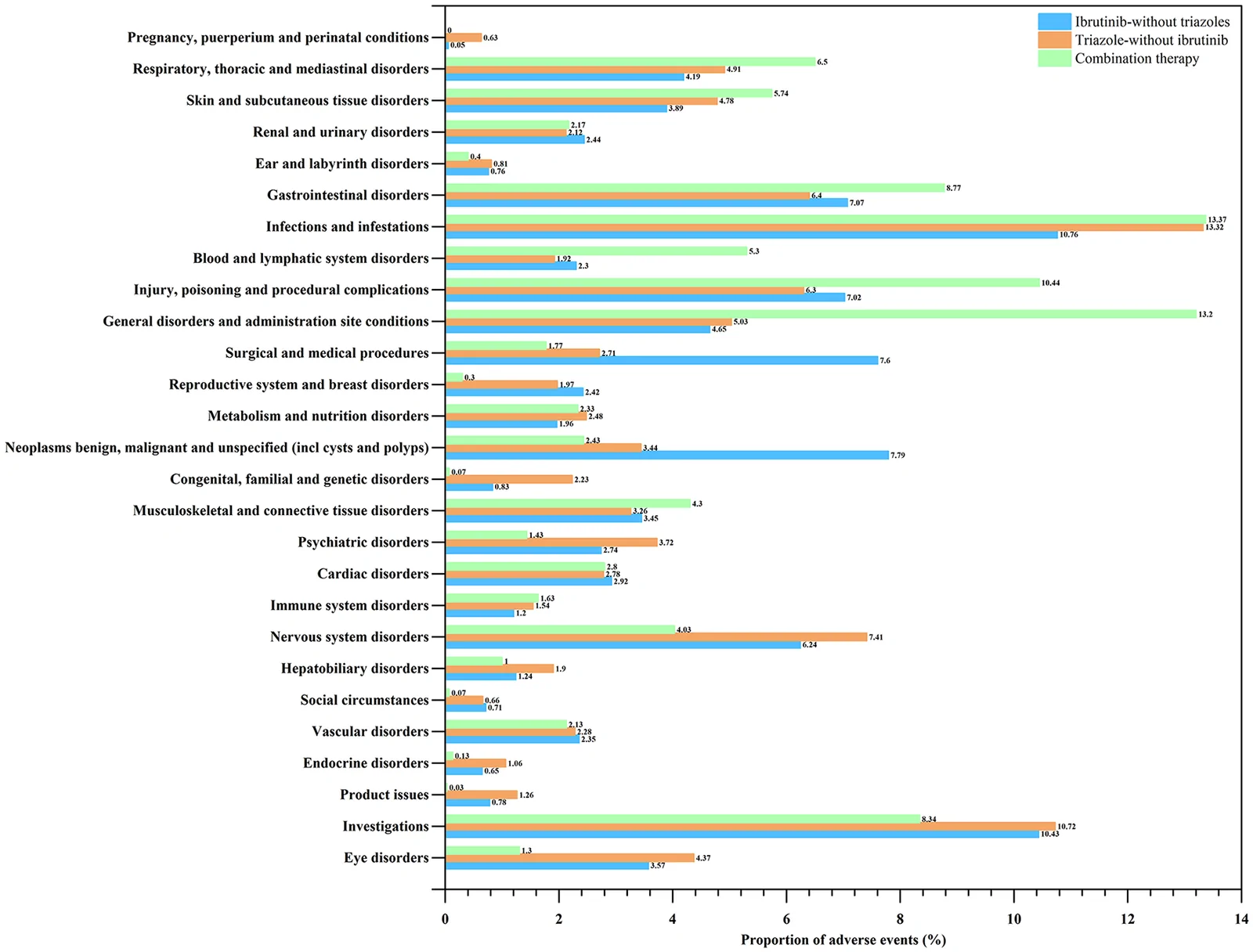
Proportion of adverse events by system organ class for ibrutinib without triazole, triazole without ibrutinib, and combination therapy.

A statistical analysis of ADEs across treatment groups was performed by System Organ Class (SOC). Figure 2 shows the reporting frequency rates of adverse events for each group. In the ibrutinib without triazole group (5,658 reports), the most common ADEs were infections and infestations (10.76%, *n* = 609), investigations (10.43%, *n* = 590), and neoplasms (7.79%, *n* = 441). In the triazole without ibrutinib group (3,955 reports), the most frequent ADEs were infections and infestations (13.32%, *n* = 527), investigations (10.72%, *n* = 424), and nervous system disorders (7.41%, *n* = 293). The combination therapy group (2,999 reports) showed distinct ADEs, with infections and infestations (13.37%, *n* = 401) and general disorders (13.2%, *n* = 396) as the most prevalent. The proportion of injury and procedural complications increased to 10.44% in the combination group, compared to 7.02% in the ibrutinib without triazole group and 6.30% in the triazole without ibrutinib group. Blood and lymphatic system disorders were also more common in the combination group (5.30%) than in the ibrutinib without triazole group (2.30%).

### 3.3 Disproportionality analysis of ADE signals

Table 2 summarizes the top 20 most frequently reported ADEs for ibrutinib, triazoles and their combinations (Supplementary Table S5 for details). In the ibrutinib without triazole group, atrial fibrillation was prominent (*n* = 2,951; ROR = 9.88, 95% CI 9.52–10.26, PRR = 9.76, χ^2^ = 22,232.52) and pneumonia was also frequent (*n* = 2,722; ROR = 2.4). In the triazole without ibrutinib group, major ADEs were drug-induced liver injury (*n* = 361; ROR = 9.02) and hepatic function abnormalities (*n* = 262; ROR = 6.71). In the combination group, pneumonia was the leading event (*n* = 55; ROR = 3.7). Pyrexia (*n* = 37; ROR = 2.34), *Aspergillus* infection (*n* = 16; ROR = 42.48) and hematological toxicities such as decreased platelet count (*n* = 30; ROR = 5.94) were more common under combination therapy.

**Table 2:** Top 20 reported adverse-event counts and disproportionality signals for ibrutinib without triazole, triazole without ibrutinib, and combination therapy.

Ibrutinib without triazole			Triazole without ibrutinib			Combination therapy		
PT	*n*	ROR (95% Cl)	PT	*n*	ROR (95% Cl)	PT	*n*	ROR (95% Cl)
Death	7,707	2.63 (2.57–2.69)	Drug interaction	2,255	13.83 (13.25–14.42)	Pneumonia	55	3.37 (2.58–4.4)
Atrial fibrillation	2,951	9.88 (9.52–10.26)	Condition aggravated	735	2.02 (1.88–2.17)	Pyrexia	37	2.34 (1.69–3.24)
Pneumonia	2,722	2.4 (2.31–2.49)	Acute kidney injury	471	2.03 (1.86–2.23)	Drug interaction	31	4.39 (3.08–6.25)
Contusion	2,563	8.25 (7.93–8.58)	Drug level increased	430	24.94 (22.64–27.47)	Disease progression	31	5.4 (3.79–7.69)
Fall	2,274	2.1 (2.01–2.19)	Hallucination	428	5.39 (4.9–5.93)	Platelet count decreased	30	5.94 (4.14–8.51)
Muscle spasms	1,619	2.67 (2.54–2.81)	Drug resistance	426	12.67 (11.5–13.95)	Atrial fibrillation	28	6.25 (4.31–9.06)
Hemorrhage	1,434	4.16 (3.95–4.39)	Treatment failure	401	3.5 (3.18–3.87)	Contusion	27	5.84 (4–8.53)
Peripheral swelling	1,405	2.2 (2.08–2.32)	*Aspergillus* infection	370	43.45 (39.09–48.29)	Anemia	22	2.53 (1.66–3.85)
Platelet count decreased	1,345	3.86 (3.66–4.07)	Drug-induced liver injury	361	9.02 (8.12–10.01)	Hemorrhage	20	3.98 (2.57–6.19)
White blood cell count increased	1,317	12.16 (11.5–12.86)	Multiple organ dysfunction syndrome	343	7.73 (6.95–8.6)	Urinary tract infection	20	2.4 (1.55–3.73)
Urinary tract infection	1,173	2.03 (1.92–2.15)	Hallucination, visual	342	16.08 (14.44–17.9)	Pleural effusion	19	7.04 (4.48–11.05)
Disease progression	1,153	2.9 (2.73–3.07)	Visual impairment	321	2.12 (1.9–2.37)	Thrombocytopenia	19	3.77 (2.4–5.92)
Hemoglobin decreased	1,082	3.41 (3.22–3.63)	Electrocardiogram qt prolonged	316	7.65 (6.84–8.55)	Febrile neutropenia	19	6.01 (3.83–9.44)
Pleural effusion	1,081	5.87 (5.53–6.24)	Photosensitivity reaction	312	17.13 (15.3–19.17)	Hemoglobin decreased	17	3.7 (2.29–5.95)
White blood cell count decreased	1,011	2.76 (2.59–2.94)	Bronchopulmonary aspergillosis	311	37.03 (33.02–41.53)	Sepsis	16	3.12 (1.91–5.11)
Epistaxis	904	3.6 (3.37–3.84)	Fungal infection	303	7.69 (6.87–8.62)	Infection	16	2.21 (1.35–3.62)
Cataract	856	4.29 (4–4.59)	Hepatotoxicity	303	12.1 (10.8–13.56)	Stomatitis	16	5.27 (3.22–8.61)
Stomatitis	791	3.78 (3.52–4.06)	Respiratory failure	279	3.71 (3.3–4.17)	Oedema peripheral	16	3.81 (2.33–6.22)
Cardiac disorder	786	2.8 (2.61–3)	Hypokalaemia	276	5.59 (4.97–6.3)	*Aspergillus* infection	16	42.48 (25.97–69.48)
Lymphadenopathy	769	7.12 (6.62–7.65)	Hepatic function abnormal	262	6.71 (5.94–7.58)	Epistaxis	14	3.83 (2.27–6.48)

**Figure 3:**
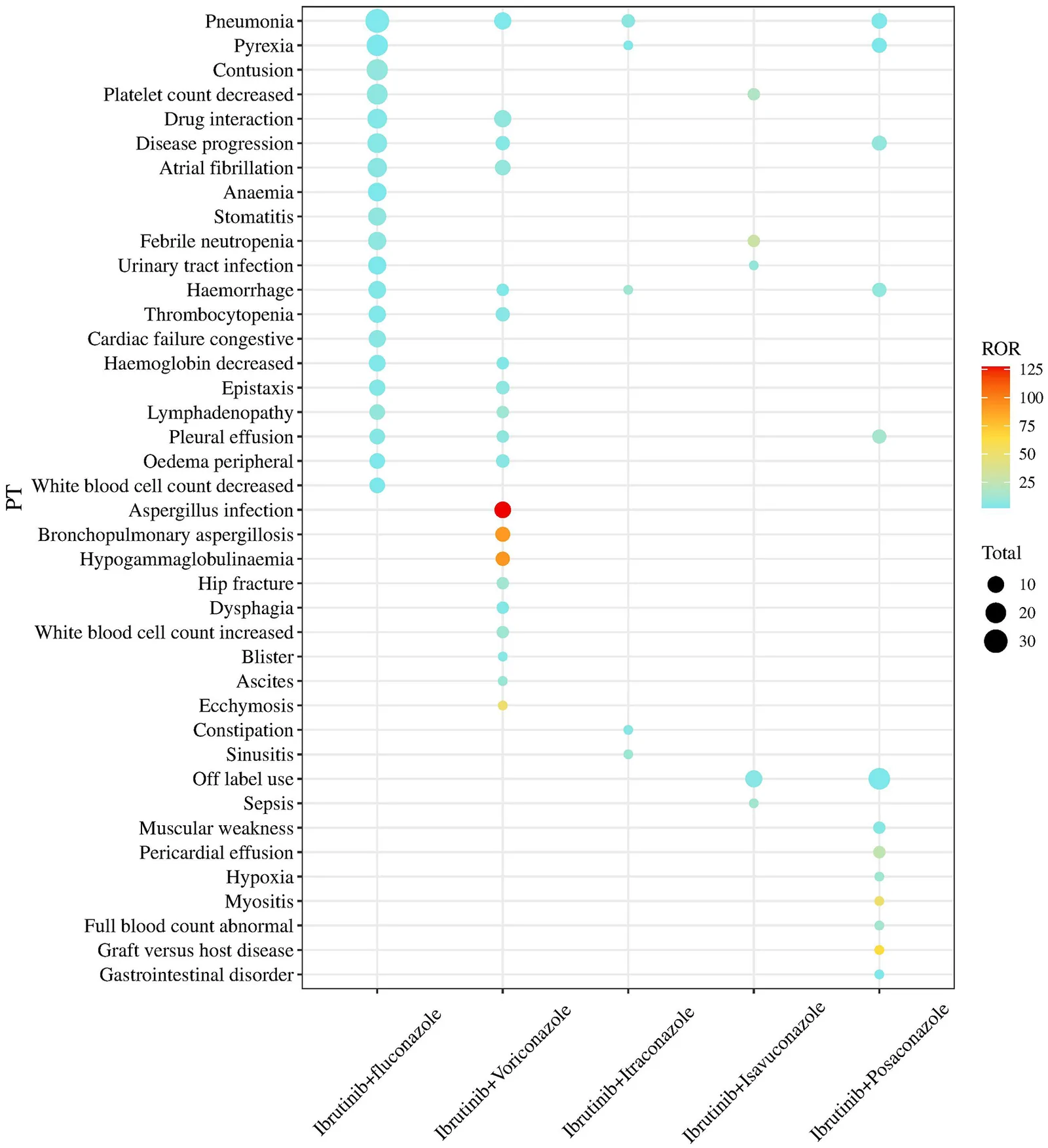
Detection of top20 adverse event signals for the combination of ibrutinib and triazole.

Figure 3 shows the ROR values at PT levels for each group. Specific numerical values can be found in Supplementary Table S6. Among combinations, ibrutinib plus fluconazole had the most cases, amplifying known ibrutinib toxicities, including contusion (*n* = 22; ROR = 8.13), decreased platelet count (*n* = 20; ROR = 6.75), and atrial fibrillation (*n* = 16; ROR = 6.08). Ibrutinib plus voriconazole showed more signals of invasive fungal infections and immunosuppression, such as *Aspergillus* infection (*n* = 10; ROR = 127.62), bronchopulmonary aspergillosis (*n* = 7; ROR = 91.05), and hypogammaglobulinaemia (*n* = 6; ROR = 92.23). The ibrutinib-itraconazole combination had the fewest reports, with main ADEs of hemorrhage (*n* = 3; ROR = 11.95), sinusitis (*n* = 3; ROR = 11.79), pneumonia (*n* = 5; ROR = 6.14), and constipation (*n* = 3; ROR = 5.71). Ibrutinib plus isavuconazole showed significant signals for febrile neutropenia (*n* = 4; ROR = 29.7), decreased platelet count (*n* = 4; ROR = 18.5), and sepsis (*n* = 3; ROR = 13.65). Ibrutinib plus posaconazole increased cardiac and muscular toxicity and graft-vs-host disease (GVHD), including pericardial effusion (*n* = 4; ROR = 24.95), muscular weakness (*n* = 4; ROR = 4.97), myositis (*n* = 3; ROR = 50.72), and GVHD (*n* = 3; ROR = 65.03).

### 3.4 TTO analysis

Table 3 shows the reporting frequency rates and TTO for ibrutinib without triazole, triazole without ibrutinib, and combination therapy by time period. For ibrutinib without triazole group, the median TTO was 183 days (IQR: 45–609), with 36.25% of reports occurring after 360 days, indicating long-term effects. The triazole without ibrutinib group median TTO was seven days (IQR: 3–20), with over 82% of reports within 30 days, indicating acute reactions. Combination therapy had a median TTO of 63 days (IQR: 19–268). Fluconazole had a median TTO of 58 days (IQR: 14–259), with 39.78% of events occurring within 30 days. Voriconazole had a median TTO of 100 days (IQR: 51–592), with 33.33% of events after 360 days. Posaconazole had a median TTO of 66 days (IQR: 32.25–304.25), similar to the overall combination group but with a broader range. Isavuconazole had the shortest median TTO (41 days, IQR: 16.5–73.75), with 66.66% of events occurring within 0–60 days. The itraconazole group was excluded due to a small sample size.

**Table 3:** Onset time of adverse events reported with ibrutinib without triazoles, triazoles without ibrutinib, and combination therapy.

Time to event onset (day)	Reports (*N*, %)							
Ibrutinib without triazoles (11,582)	Triazole without ibrutinib (4,171)	Ibrutinib+ Triazole (157)	Ibrutinib+ fluconazole (93)	Ibrutinib+ Voriconazole (27)	Ibrutinib+ Itraconazole (2)	Ibrutinib+ Isavuconazole (6)	Ibrutinib+ Posaconazole (30)
Median(IQR)	183 (45–609)	7 (3–20)	63 (19–268)	58 (14–259)	100 (51–592)	-	41 (16.5–73.75)	66 (32.25–304.25)
0–30 days	2,339 (20.2)	3,454 (82.81)	50 (31.85)	37 (39.78)	4 (14.81)	-	2 (33.33)	7 (23.33)
31–60 days	1,064 (9.19)	315 (7.55)	26 (16.56)	12 (12.9)	5 (18.52)	-	2 (33.33)	7 (23.33)
61–90 days	804 (6.94)	136 (3.26)	11 (7.01)	4 (4.3)	3 (11.11)	-	1 (16.67)	3 (10)
91–120 days	626 (5.4)	54 (1.29)	10 (6.37)	7 (7.53)	2 (7.41)	-	-	1 (3.33)
121–150 days	516 (4.46)	39 (0.94)	6 (3.82)	4 (4.3)	2 (7.41)	-	-	-
151–180 days	393 (3.39)	23 (0.55)	6 (3.82)	2 (2.15)	-	1 (50)	-	3 (10)
181–360 days	1,642 (14.18)	88 (2.11)	17 (10.83)	11 (11.83)	2 (7.41)	-	1 (16.67)	3 (10)
>360 days	4,198 (36.25)	62 (1.49)	31 (19.75)	16 (17.2)	9 (33.33)	1 (50)	–	6 (20)

It is worth noting that of the 590 reports in the combination therapy group, 433 (73.39%) were excluded from the TTO analysis due to missing or invalid dates for drug initiation or adverse event onset, leaving 157 reports (26.61%) for analysis. Comparison of included vs excluded cases revealed no significant differences in male (60.51% vs. 63.05%), 18 year ≤ Age ≤ 64 year (32.48% vs. 25.87%), serious outcomes (82.80% vs. 61.20%), suggesting that the included subset is broadly representative of the overall combination therapy population with respect to observable characteristics. Specific details are presented in Supplementary Table S7.

Weibull distribution analysis revealed that the shape parameters (β) were 0.68 for ibrutinib without triazole group, 0.64 for triazole without ibrutinib group, and 0.63 for the combination therapy group. The upper limits of the 95% confidence intervals for all groups were below 1, indicating a typical early failure pattern. Table 4 shows the median values and Weibull parameters for each treatment group.

**Table 4:** Time to onset and Weibull distribution analysis among reports with analyzable date information for adverse events reported with ibrutinib without triazoles, triazoles without ibrutinib, and combination therapy.

Drug	Cases	TTO (days)		Weibull distribution				Failure type
	*n*	Median (IQR)	Min–Max	Scale parameter		Shape parameter		
				α	95% CI	β	95% CI	
Ibrutinib without triazole	11,582	183 (45–609)	1–4,991	337.63	328.13–347.13	0.68	0.67–0.69	Early failure
Triazole without ibrutinib	4,171	7 (3–20)	1–3,820	16.19	11.67–20.71	0.64	0.57–0.71	Early failure
Combination therapy	157	63 (19–268)	1–1,869	167.82	123.86–211.78	0.63	0.56–0.71	Early failure

### 3.5 Summary of published clinical cases

After literature screening (Figure 1), 18 individual patient cases were included in the case summary (Table 5). The cohort predominantly consisted of elderly males (*n* = 13, 72.2%), with a median age of 62.5 years (range: 22–79). The vast majority of patients (patient cases *n* = 16, 88.9%) were being treated for B-cell malignancies, most commonly CLL (*n* = 14). The primary indication for triazole therapy was IFI, with aspergillosis being the most prevalent (*n* = 7, 38.9%), including several cases with central nervous system involvement.

**Table 5:** Summary of 18 individual patient cases reported in 11 publications on concomitant use of ibrutinib and triazole antifungal agents.

References	Age (year)	Gender	Indication	Ibrutinib dosage	Antifungal agent	Type of infection	Symptoms	Outcome
Gaye et al. (19)	65	Male	CLL	140 mg qd po	Voriconazole was associated with intravenous liposomal amphotericin B at 350 mg/day.	Pulmonary aspergillosis → cerebral aspergillosis	Admitted for light-headedness, balance disorder and right hemiparesis. A brain MRI scan revealed multiple nodular lesions.	Full recovery after discontinuing treatment.
Bedier et al. (20)	67	Male	CLL	140 mg qd po	Voriconazole 250 mg bid po	Cerebral aspergillosis → Facial acneiform lesions	Severe facial acneiform lesions	Complete response
Petitdemange et al. (21)	70	Not mentioned	CLL	140 mg qd po	Voriconazole 400 mg bid po	*Aspergillus* spondylodiscitis → Pulmonary Aspergillosis	Fever, and chest scan reveals pulmonary parenchymal lesions consistent with pulmonary aspergillosis.	Partial resolution
Sittig et al. (22)	72	Male	CLL	280 mg every-other-day po	Posaconazole 300 mg daily po	Cutaneous mucormycosis	At the follow-up visit after 6 months of combined medication, the patient's lesions had completely healed, and he denied any new lesions or symptoms.	Complete response
Stein et al. (23)	68	Male	CLL	420 mg qd po	Fluconazole 400 mg iv	Possible fungal infection	Atrial fibrillation	No response
Swan and Gottlieb (24)	79	Male	large B-cell lymphoma	560 mg qd po	Fluconazole 400 mg po	*Cryptococcus neoformans* empyema	Pulmonary embolism	Article recorded for further research.
De la Garza-Salazar et al. (25)	22	Male	GVHD	140 mg qd po	Itraconazole 100 mg bid po	GVHD	Shin rash, Diarrhea, abdominal pain	Complete response
Cummins et al. (26)	68	Female	CLL	140 mg qd po	Isavuconazole 200 mg qd po	*Aspergillus fumigatus*	Paroxysmal atrial fibrillation.	Passed away, presumed due to tumor progression.
	47	Female	CLL	140 mg qd po	Isavuconazole 200 mg qd po	*Histoplasma capsulatum*	No adverse events	Partial resolution
	38	Male	CLL	420 mg qd po	Isavuconazole 200 mg qd po	Probable mucormycosis	QTc interval prolongation	Complete response
	62	Male	CLL	420 mg qd po	Isavuconazole 400 mg qd po	*Aspergillus fumigatus*	Failure of antifungal therapy	After switching to voriconazole for1 year, antifungal therapy was reverted back to isavuconazole due to the emergence of significant clinical adverse reactions.
	58	Male	CLL	420 mg qd po	Isavuconazole 200 mg qd po	Probable phaeohyphomycosis	QTc interval prolongation	Complete response
	45	Female	CLL	280 mg qd po	Isavuconazole 200 mg qd po	Probable invasive aspergillosis	Clinically depressed	Passed away, presumed due to tumor progression.
	61	Male	MZL	420 mg → 280 mg qd po	Isavuconazole 200 mg qd po	Proven fusariosis	Hypoactive delirium	Complete response
	76	Male	MZL	140 mg qd po	Isavuconazole 200 mg qd po	Probable invasive aspergillosis	Thrombocytopenia	Complete response
Anonymous (27)	61	Not mentioned	CLL	420 mg qd po	Fluconazole 200 mg/24 h → 200 mg/12 h	Oropharyngeal candidiasis	Heart failure	Outcome not stated
Anonymous (28)	62	Male	CLL	Route and dosage not stated	Voriconazole 300 mg q12h po	Central Nervous System Aspergillosis	Gastrointestinal disturbances, nail changes and photosensitivity	Switch to isavuconazole after voriconazole therapy for 1 year.
Anonymous (29)	51	Male	CLL	420 mg qd po	Posaconazole and amphotericin B liposomal (not all routes and dosages stated)	Mucormycosis of the spleen, lung and the right caudate nucleus	Purpura and florid ecchymosis	Partial resolution

Analysis of clinical outcomes revealed that ADEs attributable to the drug interaction were reported in 16 cases (88.9%). Cardiac toxicity was the most prominent concern, manifesting as new-onset atrial fibrillation (*n* = 2) and QTc interval prolongation (*n* = 2). Other notable ADEs included severe cutaneous reactions (*n* = 2) and neurological symptoms (*n* = 2). Management strategies commonly involved dose adjustment or temporary discontinuation of ibrutinib. Following such interventions, complete resolution of ADEs or the infection was achieved in eight cases (44.4%). Two cases required a switch to an alternative antifungal due to poor tolerance.

### 3.6 Pharmacokinetic interactions between ibrutinib and triazole antifungals

Five pharmacokinetic studies were included, with the reported pharmacokinetic parameters summarized in Table 6. Voriconazole increased the ibrutinib area under the curve (AUC) by 5.7-fold and peak concentration (*C*_max_) by 6.7-fold. Itraconazole and posaconazole demonstrated even greater effects, increasing ibrutinib AUC by approximately 10-fold and 9.5- to 10.3-fold, respectively. The study on posaconazole concluded that staggering the administration times was ineffective in mitigating this interaction. The AUC ratio of the active metabolite PCI-45227 to ibrutinib was significantly decreased across these strong inhibitors, indicating a suppression of ibrutinib's metabolic clearance. The magnitude of interaction was moderate with the weaker CYP3A4 inhibitors. Isavuconazole co-administration led to an approximate 2.1-fold increase in ibrutinib AUC. During concomitant use of fluconazole, the *C*_max_ of ibrutinib in patients was approximately 1.2 times higher than when administered alone.

**Table 6:** Pharmacokinetic interactions between ibrutinib and triazole antifungal agents.

References	Study population	Interacting drug	Ibrutinib dose	Azole dose	Dosing conditions	Effect on ibrutinib AUC	Effect on ibrutinib *C*_max_	PCI-45227/ibrutinib AUC ratio	Notes
de Jong et al. (30)	Patients with B-cell malignancies (*n* = 26)	Voriconazole	140 mg qd (mono), 560 mg (combination)	200 mg po bid	Fed state	↑5.7–fold	↑ 6.7–fold	Decreased from 1.20 to 0.37	Strong CYP3A4 inhibitor; significant exposure increase
Olkkola et al. (31)	Healthy participants (*n* = 11)	Posaconazole	140 mg (mono), 30 mg (with morning posaconazole)/70 mg (with evening posaconazole)	300 mg po qd	Morning dose 1 h before ibrutinib or 12 h apart (evening)	↑ 9.5–fold/↑ 10.3–fold	↑ 8.5–fold/↑ 8.2–fold	Decreased from 3.7 to 0.5/0.54	Staggered dosing ineffective; recommend ≤ 70 mg/day ibrutinib
Tapaninen et al. (32)	Healthy participants (*n* = 11)	Itraconazole	15 mg qd (with itraconazole) vs 140 mg qd (mono)	200 mg po bid (day 1), 200 mg qd (days 2–4)	Fed state	↑ 10–fold	↑ 8.8–fold	Reduced from 3.4 to 0.34	Strong CYP3A4 inhibition; variability reduced by ~50%
Goosen et al. (33)	Healthy participants (*n* = 100)	Isavuconazole	420 mg	200 mg po qd	Overnight fast	≈2.1–fold increase	Not reported	Not reported	Moderate CYP3A4 inhibition; clinical relevance moderate
Yasu et al. (34)	Patient	Fluconazole	540 mg	100 mg po qd	Not reported	Not reported	↑1.2–fold (approx.)	Not reported	Moderate inhibitor; potential for higher effect at higher doses

## 4 Discussion

As a cornerstone therapy for B cell malignancies, ibrutinib's clinical utility is complicated by its pharmacokinetic vulnerability as a sensitive CYP3A4 substrate (35, 36). By systematically integrating over 15.5 million real-world FAERS reports with clinical pharmacokinetic data and case series, this study bridges a critical gap in the mechanistic-to-clinical evidence chain. Our findings identified the severe clinical consequences of ibrutinib-triazole co-administration, revealing that combination therapy results in serious outcomes in 66.95% of cases, compared with 46.38% for ibrutinib without triazole and 46.85% for triazole without ibrutinib, respectively. Crucially, the toxicity profile of the combination is not merely additive but synergistic, manifesting as a distinct risk gradient that directly corresponds to the CYP3A4 inhibitory potency of the specific triazole agent.

### 4.1 Increased risk of bleeding

The proportion of injury, toxicity, and procedural complication reports was 10.44% with concomitant exposure, compared with 7.02% and 6.30% in the comparator groups, and was accompanied by signals such as contusion and hemorrhage. These observations are compatible with the known effects of ibrutinib on platelet function (37). Elevated signals for petechiae (ROR = 26.74, *n* = 7) and ecchymosis (ROR = 30.28, *n* = 5) were observed in the fluconazole subgroup. One published case report documented the emergence of pronounced purpura and florid ecchymosis in a patient receiving ibrutinib concurrently with posaconazole and amphotericin B (29). This case is clinically consistent with bleeding during concomitant exposure. The pattern is plausible because CYP3A4 inhibition may increase ibrutinib exposure and thereby amplify its effects on platelet function. In real-world clinical practice, it is noteworthy that many patients receiving ibrutinib for B-cell malignancies may also require antiplatelet therapy for cardiovascular comorbidities or anticoagulation for atrial fibrillation. The concurrent use of antiplatelet or anticoagulant agents with ibrutinib has been shown to increase the risk of clinically relevant bleeding by approximately 2.5-fold (aOR 2.54, 95% CI: 1.94–3.32) (38). Moreover, retrospective data indicate that major bleeding occurs in up to 8% of patients receiving ibrutinib with therapeutic anticoagulation, with incidence rates as high as 18% for those on rivaroxaban (39). The combination of ibrutinib with triazole antifungals, would be expected to exacerbate this bleeding events in a potentially synergistic manner. Consequently, clinicians should exercise extreme caution when considering the addition of any antiplatelet or anticoagulant therapy in patients already receiving an ibrutinib-triazole combination, carefully weighing thromboembolic risk against bleeding risk on an individual patient basis. When such co-administration is unavoidable, close monitoring for signs of bleeding and consideration of ibrutinib dose reduction are strongly advised.

### 4.2 Increased hematological toxicity

The reporting frequency of blood and lymphatic disorders in the combination therapy group was 5.3%, exceeding the rates observed in the ibrutinib without triazole group (2.3%) and triazole without ibrutinib group (1.9%). Among the top 20 strongest signals for the combination therapy were several hematological toxicities, including platelet count decreased (ROR = 5.94, *n* = 30), febrile neutropenia (ROR = 6.01, *n* = 19), and anemia (ROR = 2.53, *n* = 22). Febrile neutropenia was more frequently reported in the combination therapy group. Neutropenia substantially impairs immune defense against pathogens (40). Current data on the combination of ibrutinib and isavuconazole suggest a hematologic reporting signal that warrants clinical attention. A disproportionality for sepsis was also noted in the isavuconazole subgroup (ROR = 13.65, *n* = 3), though this finding is based on few cases and should be interpreted with caution. Early identification of sepsis risk factors, such as decreased platelet count, and implementation of preventive interventions have been shown to reduce mortality (41). This represents a novel signal requiring particular attention in clinical practice.

### 4.3 Infection as a primary safety risk

Infection-related reporting signals, particularly IFIs, observed in the FAERS database are further supported by the detailed clinical accounts from published clinical cases. The disproportionality analysis identified a strong signal for *Aspergillus* infection in the combination therapy group (ROR = 42.48, *n* = 16). Among the 18 individual patient cases, aspergillosis was the most common specific infection, occurring in seven patients (38.9%). Furthermore, a small-count disproportionality signal for cerebral aspergillosis in the ibrutinib-voriconazole group (ROR = 474.61, *n* = 3) is consistent with reports by Gaye et al. (19) and Bedier et al (20), in which patients receiving this combination developed life-threatening cerebral aspergillosis. This signal should be considered hypothesis-generating and requires validation in larger datasets. Similar consistency was observed for bronchopulmonary aspergillosis and mucormycosis, both of which were identified in the FAERS analysis and the published literature (22). The disproportionality signal for hypogammaglobulinaemia (ROR = 92.23, *n* = 6) may reflect the immune dysfunction associated with ibrutinib therapy. Sun et al. (42) demonstrated that prolonged ibrutinib treatment is associated with declining serum IgG levels and secondary hypogammaglobulinaemia. In addition, from a mechanistic perspective, BTK plays a critical role in neutrophil-mediated antifungal immunity. Upon fungal exposure, BTK in human neutrophils is activated in a TLR2-, Dectin-1-, and FcγR-dependent manner, triggering the oxidative burst essential for killing *Aspergillus* (43). Ibrutinib, by inhibiting BTK, selectively impairs neutrophil-mediated damage to *Aspergillus* hyphae, primary granule release, and the fungus-induced oxidative burst through abrogation of NADPH oxidase subunit p40phox and GTPase RAC2 activation (43). Moreover, Blez et al. (44) demonstrated that neutrophils from ibrutinib-treated patients exhibit decreased production of reactive oxygen species, impaired engulfment of *Aspergillus*, and inability to efficiently kill germinating conidia. These findings establish IFIs as a pharmacologically predictable adverse outcome of BTK inhibition rather than merely an incidental indication for triazole prescription.

However, several infection-related signals identified in this study, including cerebral aspergillosis, bronchopulmonary aspergillosis, and mucormycosis, may also represent drug-induced adverse reactions attributable to ibrutinib without concomitant triazole therapy, given that these infections are typically clinical indications for initiating triazole-based treatment. Therefore, the observed associations should be interpreted cautiously. While our findings support increased vigilance for opportunistic fungal infections in patients receiving concomitant azole antifungals and ibrutinib, they do not establish a causal drug-drug interaction.

### 4.4 Persistent risk of cardiac toxicity

Ibrutinib has been associated with serious cardiac arrhythmias and heart failure (45). In this study, atrial fibrillation was frequently reported in both the ibrutinib without triazole group and combination therapy group. Pleural effusion was more often reported in the combination therapy group. In the posaconazole subgroup, disproportionalities for pericardial effusion (ROR = 24.95, *n* = 4) and pleural effusion (ROR = 14.43, *n* = 6) were detected. While based on limited case numbers and requiring cautious interpretation, these signals are mechanistically plausible given posaconazole's potent CYP3A4 inhibition. The case series involving isavuconazole also described QTc interval prolongation, and itraconazole exhibits negative inotropic effects (46). The fluconazole group showed a reporting signal for congestive cardiac failure (ROR = 5.73, *n* = 11). These quantitative signals are directly and powerfully substantiated by the clinical outcomes documented in the published cases. Among the 18 cases, cardiac toxicity was the most frequently reported adverse event, with three separate cases documenting atrial fibrillation or heart failure (23, 26, 27) and two cases explicitly reporting QTc prolongation in patients receiving the ibrutinib-isavuconazole combination (26). This collective evidence indicates that cardiac toxicity is not an isolated issue but rather a widespread threat that requires routine cardiac monitoring.

Our study integrates pharmacokinetic data with clinical reporting signals to provide a plausible explanation for several observed FAERS signals. Potent CYP3A4 inhibitors (voriconazole, itraconazole, posaconazole) increased ibrutinib AUC by 5.7- to 10.3-fold, which may help explain the observed reporting patterns, including voriconazole-associated aspergillosis and posaconazole-associated cardiac or muscular events. Isavuconazole may be considered when a lower drug-interaction potential is clinically desirable because of its relatively weaker inhibitory effect on CYP3A4 (47). A single-center retrospective cohort study similarly suggested differences in CYP3A-mediated interaction potential among triazoles, as patients receiving isavuconazole required lower doses of immunosuppressants than those receiving other triazole agents (48).

## 5 Study limitations

The FAERS-based analyses are subject to the limitations of spontaneous reporting systems, including under-reporting, selective reporting, and the absence of a defined denominator population. Disproportionality analyses are intended to identify signals of association rather than establish causality. Moreover, some signals were based on a small number of reports (*n* = 3–4). Under these conditions, ROR estimates may be sensitive to fluctuations in reporting counts and may overestimate the strength of association. These findings should therefore be considered hypothesis-generating and require further evaluation. Additionally, the TTO analysis should be interpreted with caution. Due to missing or incomplete date fields inherent in spontaneous reporting, approximately 73.39% of reports in the combination group were excluded from the temporal analysis. Although a baseline comparison between included and excluded cases showed no major discrepancies, this substantial missingness limits the generalizability of our temporal conclusions. Furthermore, residual confounding cannot be excluded. The potential for confounding by indication is complicated by ibrutinib's intrinsic immunosuppressive effects, which themselves increase susceptibility to invasive fungal infections. Notably, the FAERS database does not systematically capture complete information on concomitant use of antiplatelet or anticoagulant agents, which may be important confounders in the assessment of bleeding risk. The lack of such data in a substantial proportion of reports precludes a quantitative sub-analysis of this specific interaction. The integrated case series is also limited by its small sample size and the possibility of publication bias, which restrict its value for independent causal inference. Finally, the pharmacokinetic evidence is derived primarily from controlled experimental settings, and its applicability to patients with complex comorbidities in routine clinical practice may be limited. Despite these limitations, findings from the different data sources were generally consistent and may contribute to understanding the safety profile of the ibrutinib-triazole interaction.

## 6 Conclusions

This study identifies critical real-world pharmacovigilance signals that align with established pharmacokinetic interactions between ibrutinib and triazole antifungals. The findings demonstrate that the severity and specific phenotypes of ibrutinib-associated toxicities are appears to be associated with the CYP3A4 inhibitory potency of the concurrent triazole. Potent inhibitors not only induce new, life-threatening complications but also may be associated with earlier onset, driving a “early failure” pattern. While these findings do not establish critical causality, they strongly underscore the need for clinical vigilance. Consequently, clinicians might consider adopting a risk-stratified management paradigm: implementing preemptive ibrutinib dose reductions proportional to the triazole's potency, prioritizing moderate inhibitors (e.g., isavuconazole) when clinically feasible, and executing a dual-focused monitoring strategy to intercept acute events while surveilling for cumulative toxicities.

## Data Availability

The original contributions presented in the study are included in the article/Supplementary material, further inquiries can be directed to the corresponding author.
