# Supplementary material for: Risk stratification of ibrutinib toxicity co-administered with triazole antifungals: insights from real-world pharmacovigilance and clinical evidence
Source: Front Med (Lausanne). 2026 Aug 31;13:1875804. doi: 10.3389/fmed.2026.1875804 (PMC13572604; doi:10.3389/fmed.2026.1875804)
Supplement: Supplementary file 1 [file Table_1.docx]

### Table S1 Standard drug name and brand names in FAERS database

| Standard drug name | Brand names |
| --- | --- |
| Ibrutinib | Imbruvica, Ibrunex |
| Fluconazole | Diflucan, Apo-fluconazole, Apo fluconazole, Fluc Hexal, Flucobeta, Flucolich, Fluconazol AbZ, Fluconazol AL, Fluconazol STADA, Fluconazol von CT, Fluconazol-ISIS, Fluconazol ISIS, Fluconazol-ratiopharm, Fluconazol ratiopharm, Flunazul, Fungata, Lavisa, Loitin, Neofomiral, Oxifungol, Solacap, Zonal, Triflucan |
| Voriconazole | Vfend, Voriconazole Hikma |
| Isavuconazole | Cresemba |
| Posaconazole | Noxafil, Posanol, Posaconazole AHCL, Posaconazole Accord |
| Itraconazole | Sporanox, Orungal, Tolsura, Onmel |
