# Supplementary material for: Risk stratification of ibrutinib toxicity co-administered with triazole antifungals: insights from real-world pharmacovigilance and clinical evidence
Source: Front Med (Lausanne). 2026 Aug 31;13:1875804. doi: 10.3389/fmed.2026.1875804 (PMC13572604; doi:10.3389/fmed.2026.1875804)
Supplement: Supplementary file 2 [file Table_2.docx]

Table S2 Platform-specific search strategies for published clinical cases and human pharmacokinetic evidence involving ibrutinib and triazole antifungal agents

| Database: PubMed <From inception to November 3, 2025> Fields: Title/Abstract; limits: none for language. | |
| --- | --- |
| **#** | **Searches** |
| 1 | ("ibrutinib") AND ("fluconazole") |
| 2 | ("ibrutinib") AND ("voriconazole") |
| 3 | ("ibrutinib") AND ("posaconazole") |
| 4 | ("ibrutinib") AND ("itraconazole") |
| 5 | ("ibrutinib") AND ("isavuconazole") |
| 6 | ("ibrutinib") AND ("triazole") |
| Database: Embase <From inception to November 3, 2025> Fields: title, abstract, keyword heading word; limits: none for language. | |
| **#** | **Searches** |
| 1 | (ibrutinib) and (fluconazole) |
| 2 | (ibrutinib) and (voriconazole) |
| 3 | (ibrutinib) and (posaconazole) |
| 4 | (ibrutinib) and (itraconazole) |
| 5 | (ibrutinib) and (isavuconazole). |
| 6 | (ibrutinib) and (triazole) |
| Database: Web of Science Core Collection <From inception to November 3, 2025> Field: Title/Abstract; limits: none for language. | |
| **#** | **Searches** |
| 1 | ("ibrutinib") AND ("fluconazole") |
| 2 | ("ibrutinib") AND ("voriconazole") |
| 3 | ("ibrutinib") AND ("posaconazole") |
| 4 | ("ibrutinib") AND ("itraconazole") |
| 5 | ("ibrutinib") AND ("isavuconazole") |
| 6 | ("ibrutinib") AND ("triazole") |
| Database: SpringerLink <From inception to November 3, 2025> Fields: platform full-text/default relevance search; limits: none for language. | |
| **#** | **Searches** |
| 1 | ("ibrutinib") AND ("fluconazole") |
| 2 | ("ibrutinib") AND ("voriconazole") |
| 3 | ("ibrutinib") AND ("posaconazole") |
| 4 | ("ibrutinib") AND ("itraconazole") |
| 5 | ("ibrutinib") AND ("isavuconazole") |
| 6 | ("ibrutinib") AND ("triazole") |
| *Search notes: The six component searches were run separately and their results were combined within each database. | |
| Deduplication and PRISMA accounting: PubMed (n = 28) + Embase (n = 48) + Web of Science (n = 96) + SpringerLink (n = 64) = 236 records. EndNote's Find Duplicates function and manual comparison of DOI, PMID, normalized title, first author, and publication year removed 64 duplicates, leaving 172 unique records. Of these, 127 were excluded during title/abstract screening; 45 full texts were assessed; 29 were excluded; and 16 publications were included. | |
