# Supplementary material for: Risk stratification of ibrutinib toxicity co-administered with triazole antifungals: insights from real-world pharmacovigilance and clinical evidence
Source: Front Med (Lausanne). 2026 Aug 31;13:1875804. doi: 10.3389/fmed.2026.1875804 (PMC13572604; doi:10.3389/fmed.2026.1875804)
Supplement: Supplementary file 3 [file Table_3.docx]

Table S3 Two-by-two contingency table for disproportionality analyses

|  | Target AE | Other AE | Total |
| --- | --- | --- | --- |
| Target drug | a | b | a+b |
| Other drugs | c | d | c+d |
| Total | a+c | b+d | a+b+c+d |
