# Supplementary material for: Risk stratification of ibrutinib toxicity co-administered with triazole antifungals: insights from real-world pharmacovigilance and clinical evidence
Source: Front Med (Lausanne). 2026 Aug 31;13:1875804. doi: 10.3389/fmed.2026.1875804 (PMC13572604; doi:10.3389/fmed.2026.1875804)
Supplement: Supplementary file 4 [file Table_4.docx]

Table S4 Summary of major algorithms used for signal detection

| Algorithms | Equation | Criteria |
| --- | --- | --- |
| ROR | ROR=ad/b/c | lower limit of 95% CI>1, N≥3 |
|  | 95%CI=e^ln(ROR)±1.96(1/a+1/b+1/c+1/d)^0.5^ |  |
| PRR | PRR=a(c+d)/c/(a+b) | PRR≥2, χ^2^≥4, N≥3 |
|  | χ^2^=[(ad-bc)^2](a+b+c+d)/[(a+b)(c+d)(a+c)(b+d)] |  |

Equation: a, number of reports containing both the target drug and target adverse drug reactions; b, number of reports containing other drug adverse reactions that contain the targeted drug; c, number of reports containing the target adverse drug reaction of other drugs; d, number of reports containing other drugs and other adverse drug reactions. 95%CI, 95% confidence interval; N, the number of reports; χ^2^, chi-squared.
