# Supplementary material for: Risk stratification of ibrutinib toxicity co-administered with triazole antifungals: insights from real-world pharmacovigilance and clinical evidence
Source: Front Med (Lausanne). 2026 Aug 31;13:1875804. doi: 10.3389/fmed.2026.1875804 (PMC13572604; doi:10.3389/fmed.2026.1875804)
Supplement: Supplementary file 5 [file Table_5.docx]

Table S5 Report frequency of adverse drug events signals for ibrutinib, triazole, and the combination of ibrutinib and triazole (in descending order).

| Ibrutinib | | | Triazole | | | Combination therapy | | |
| --- | --- | --- | --- | --- | --- | --- | --- | --- |
| PT | n | ROR(95%Cl) | PT | n | ROR(95%Cl) | PT | n | ROR(95%Cl) |
| Death | 7707 | 2.63(2.57-2.69) | Drug interaction | 2255 | 13.83(13.25-14.42) | Pneumonia | 55 | 3.37(2.58-4.4) |
| Atrial fibrillation | 2951 | 9.88(9.52-10.26) | Condition aggravated | 735 | 2.02(1.88-2.17) | Pyrexia | 37 | 2.34(1.69-3.24) |
| Pneumonia | 2722 | 2.4(2.31-2.49) | Acute kidney injury | 471 | 2.03(1.86-2.23) | Drug interaction | 31 | 4.39(3.08-6.25) |
| Contusion | 2563 | 8.25(7.93-8.58) | Drug level increased | 430 | 24.94(22.64-27.47) | Disease progression | 31 | 5.4(3.79-7.69) |
| Fall | 2274 | 2.1(2.01-2.19) | Hallucination | 428 | 5.39(4.9-5.93) | Platelet count decreased | 30 | 5.94(4.14-8.51) |
| Muscle spasms | 1619 | 2.67(2.54-2.81) | Drug resistance | 426 | 12.67(11.5-13.95) | Atrial fibrillation | 28 | 6.25(4.31-9.06) |
| Haemorrhage | 1434 | 4.16(3.95-4.39) | Treatment failure | 401 | 3.5(3.18-3.87) | Contusion | 27 | 5.84(4-8.53) |
| Peripheral swelling | 1405 | 2.2(2.08-2.32) | Aspergillus infection | 370 | 43.45(39.09-48.29) | Anaemia | 22 | 2.53(1.66-3.85) |
| Platelet count decreased | 1345 | 3.86(3.66-4.07) | Drug-induced liver injury | 361 | 9.02(8.12-10.01) | Haemorrhage | 20 | 3.98(2.57-6.19) |
| White blood cell count increased | 1317 | 12.16(11.5-12.86) | Multiple organ dysfunction syndrome | 343 | 7.73(6.95-8.6) | Urinary tract infection | 20 | 2.4(1.55-3.73) |
| Urinary tract infection | 1173 | 2.03(1.92-2.15) | Hallucination, visual | 342 | 16.08(14.44-17.9) | Pleural effusion | 19 | 7.04(4.48-11.05) |
| Disease progression | 1153 | 2.9(2.73-3.07) | Visual impairment | 321 | 2.12(1.9-2.37) | Thrombocytopenia | 19 | 3.77(2.4-5.92) |
| Haemoglobin decreased | 1082 | 3.41(3.22-3.63) | Electrocardiogram qt prolonged | 316 | 7.65(6.84-8.55) | Febrile neutropenia | 19 | 6.01(3.83-9.44) |
| Pleural effusion | 1081 | 5.87(5.53-6.24) | Photosensitivity reaction | 312 | 17.13(15.3-19.17) | Haemoglobin decreased | 17 | 3.7(2.29-5.95) |
| White blood cell count decreased | 1011 | 2.76(2.59-2.94) | Bronchopulmonary aspergillosis | 311 | 37.03(33.02-41.53) | Sepsis | 16 | 3.12(1.91-5.11) |
| Epistaxis | 904 | 3.6(3.37-3.84) | Fungal infection | 303 | 7.69(6.87-8.62) | Infection | 16 | 2.21(1.35-3.62) |
| Cataract | 856 | 4.29(4-4.59) | Hepatotoxicity | 303 | 12.1(10.8-13.56) | Stomatitis | 16 | 5.27(3.22-8.61) |
| Stomatitis | 791 | 3.78(3.52-4.06) | Respiratory failure | 279 | 3.71(3.3-4.17) | Oedema peripheral | 16 | 3.81(2.33-6.22) |
| Cardiac disorder | 786 | 2.8(2.61-3) | Hypokalaemia | 276 | 5.59(4.97-6.3) | Aspergillus infection | 16 | 42.48(25.97-69.48) |
| Lymphadenopathy | 769 | 7.12(6.62-7.65) | Hepatic function abnormal | 262 | 6.71(5.94-7.58) | Epistaxis | 14 | 3.83(2.27-6.48) |
| Thrombocytopenia | 722 | 2.06(1.92-2.22) | Septic shock | 251 | 5.27(4.66-5.97) | Cardiac failure congestive | 14 | 4.27(2.53-7.22) |
| Increased tendency to bruise | 660 | 24.25(22.37-26.28) | Cholestasis | 247 | 12.25(10.8-13.9) | Lymphadenopathy | 13 | 8.15(4.73-14.06) |
| Chronic lymphocytic leukaemia | 582 | 45.12(41.26-49.34) | Renal impairment | 239 | 2.44(2.15-2.77) | White blood cell count increased | 13 | 7.94(4.6-13.69) |
| Onychoclasis | 572 | 24.95(22.88-27.21) | Mucormycosis | 214 | 64.66(56.16-74.44) | Petechiae | 12 | 26.92(15.26-47.48) |
| Cardiac failure congestive | 521 | 2.29(2.1-2.5) | Pathogen resistance | 212 | 18.18(15.85-20.84) | Dysphagia | 11 | 2.48(1.37-4.48) |
| Full blood count abnormal | 490 | 4.88(4.46-5.34) | Febrile neutropenia | 206 | 2.71(2.36-3.1) | Gastrointestinal haemorrhage | 11 | 2.68(1.48-4.84) |
| Hospice care | 476 | 12.64(11.52-13.87) | Delirium | 203 | 5.45(4.74-6.25) | White blood cell count decreased | 11 | 2.07(1.15-3.74) |
| Pulmonary oedema | 464 | 3.39(3.09-3.72) | Neurotoxicity | 195 | 9.41(8.17-10.84) | Bronchopulmonary aspergillosis | 11 | 29.85(16.5-53.99) |
| Hip fracture | 457 | 4.45(4.06-4.88) | Alanine aminotransferase increased | 189 | 3.42(2.97-3.95) | Fungal infection | 10 | 6.04(3.25-11.24) |
| Bone pain | 452 | 2.33(2.12-2.56) | Pancytopenia | 187 | 3.15(2.73-3.63) | Hypogammaglobulinaemia | 10 | 32.12(17.25-59.79) |
| White blood cell count abnormal | 436 | 20.16(18.28-22.24) | Hepatic enzyme increased | 184 | 2.39(2.07-2.77) | Increased tendency to bruise | 10 | 23.15(12.44-43.09) |
| Petechiae | 431 | 14.71(13.34-16.22) | Abortion spontaneous | 180 | 4.36(3.76-5.05) | Septic shock | 9 | 4.52(2.35-8.69) |
| Haematuria | 429 | 3.89(3.54-4.28) | Cardiac arrest | 179 | 2.23(1.93-2.59) | Respiratory failure | 9 | 2.86(1.49-5.51) |
| Cerebral haemorrhage | 421 | 3.67(3.33-4.04) | Rhabdomyolysis | 174 | 4.5(3.87-5.22) | Full blood count abnormal | 9 | 6.14(3.19-11.81) |
| Haematoma | 415 | 5.13(4.65-5.65) | Hepatic cytolysis | 167 | 11.25(9.65-13.11) | Chronic lymphocytic leukaemia | 9 | 40.51(21.04-78.01) |
| Pericardial effusion | 392 | 5.52(4.99-6.1) | Drug level decreased | 165 | 13.47(11.54-15.71) | Neutrophil count decreased | 9 | 4.6(2.39-8.84) |
| Arrhythmia | 391 | 2.85(2.58-3.15) | Candida infection | 162 | 7.17(6.14-8.37) | Leukocytosis | 8 | 10.53(5.26-21.08) |
| Cellulitis | 389 | 2.37(2.15-2.62) | Aspartate aminotransferase increased | 161 | 3.52(3.02-4.11) | Ecchymosis | 8 | 28.45(14.2-56.97) |
| Red blood cell count decreased | 388 | 4.11(3.72-4.55) | Periostitis | 160 | 626.19(503.08-779.44) | Pancytopenia | 8 | 3.23(1.61-6.46) |
| Rash macular | 357 | 3.07(2.76-3.41) | Hyperkalaemia | 146 | 3.97(3.38-4.68) | Cellulitis | 8 | 3.38(1.69-6.77) |
| Laboratory test abnormal | 339 | 3.08(2.76-3.42) | Hyponatraemia | 145 | 2.4(2.04-2.83) | Deep vein thrombosis | 8 | 3.11(1.56-6.23) |
| Skin cancer | 333 | 4.21(3.78-4.7) | Hepatocellular injury | 140 | 7.48(6.33-8.84) | Cataract | 7 | 2.4(1.14-5.04) |
| Skin discolouration | 325 | 2.1(1.89-2.35) | Liver injury | 139 | 5.05(4.27-5.97) | Haematoma | 7 | 5.91(2.82-12.42) |
| Subdural haematoma | 302 | 6.48(5.78-7.27) | Blister | 137 | 2.1(1.78-2.49) | Cerebral haemorrhage | 7 | 4.19(2-8.81) |
| Impaired healing | 298 | 2.92(2.61-3.27) | Drug reaction with eosinophilia and systemic symptoms | 137 | 3.97(3.35-4.69) | Pericardial effusion | 7 | 6.73(3.2-14.13) |
| Night sweats | 283 | 2.64(2.35-2.97) | Haemoptysis | 135 | 4.33(3.65-5.13) | Graft versus host disease | 7 | 23.45(11.16-49.26) |
| Haemoptysis | 282 | 3.14(2.79-3.53) | Rash maculo-papular | 134 | 5.63(4.75-6.68) | Dysuria | 6 | 3.61(1.62-8.04) |
| Localised infection | 280 | 3.29(2.92-3.7) | Liver function test abnormal | 133 | 5.48(4.62-6.5) | Hip fracture | 6 | 4(1.8-8.92) |
| Medical procedure | 269 | 55.62(48.65-63.6) | Pleural effusion | 131 | 2.01(1.69-2.39) | Pneumonia fungal | 6 | 27.22(12.21-60.67) |
| Skin laceration | 266 | 4.39(3.89-4.96) | Torsade de pointes | 130 | 17.51(14.71-20.85) | Pulmonary mass | 6 | 7.43(3.34-16.56) |
| Eye haemorrhage | 265 | 6.47(5.73-7.31) | Altered state of consciousness | 128 | 5.23(4.39-6.22) | Haematuria | 6 | 3.74(1.68-8.33) |
| Hernia | 264 | 4.01(3.55-4.52) | Transaminases increased | 126 | 5.06(4.24-6.03) | Subdural haematoma | 6 | 8.75(3.93-19.5) |
| Post procedural haemorrhage | 261 | 7.91(6.99-8.95) | International normalised ratio increased | 125 | 5.61(4.71-6.7) | Bone marrow transplant | 6 | 79.81(35.75-178.17) |
| Skin lesion | 256 | 2.86(2.53-3.24) | Gamma-glutamyltransferase increased | 124 | 6.48(5.43-7.74) | Mantle cell lymphoma | 6 | 80.17(35.91-178.96) |
| Skin ulcer | 256 | 3.02(2.67-3.41) | Rash erythematous | 123 | 2.46(2.06-2.94) | Haemorrhage subcutaneous | 6 | 35.16(15.77-78.4) |
| Head injury | 255 | 2.52(2.22-2.85) | Liver disorder | 118 | 2.49(2.08-2.99) | Chronic graft versus host disease | 6 | 36.84(16.52-82.14) |
| Blood urine present | 255 | 4.08(3.61-4.62) | Mental disorder | 116 | 2.47(2.06-2.97) | Immunodeficiency | 5 | 6.24(2.6-15.02) |
| Skin disorder | 253 | 2.19(1.93-2.48) | Acute myeloid leukaemia | 116 | 6.98(5.82-8.39) | Cholelithiasis | 5 | 4.07(1.69-9.79) |
| Gout | 248 | 4.16(3.67-4.72) | Squamous cell carcinoma of skin | 113 | 15.57(12.92-18.77) | Staphylococcal infection | 5 | 3.63(1.51-8.74) |
| Cystitis | 246 | 2.33(2.06-2.65) | Arrhythmia | 111 | 2.33(1.93-2.8) | Mental status changes | 5 | 4.58(1.9-11) |
| Skin haemorrhage | 241 | 4.74(4.17-5.38) | Pneumonia fungal | 110 | 21.36(17.66-25.84) | Red blood cell count decreased | 5 | 3.64(1.51-8.75) |
| Nail disorder | 236 | 8.36(7.34-9.52) | Lip swelling | 110 | 2.97(2.46-3.58) | Autoimmune haemolytic anaemia | 5 | 25.88(10.76-62.28) |
| Knee arthroplasty | 236 | 3.35(2.95-3.82) | Blood alkaline phosphatase increased | 109 | 5.33(4.42-6.44) | Liver function test abnormal | 5 | 4.92(2.05-11.84) |
| Transient ischaemic attack | 227 | 2.5(2.19-2.85) | Leukaemia | 104 | 9.32(7.68-11.31) | Lymphoma | 4 | 5.45(2.05-14.54) |
| Lymphocytosis | 219 | 38.8(33.6-44.8) | Actinic keratosis | 102 | 34.94(28.62-42.65) | Gout | 4 | 4.61(1.73-12.29) |
| Immunodeficiency | 217 | 3.94(3.45-4.51) | Pseudoaldosteronism | 100 | 1259.16(896.63-1768.28) | Skin cancer | 4 | 3.47(1.3-9.26) |
| Splenomegaly | 216 | 5.91(5.16-6.76) | Systemic mycosis | 96 | 82.23(66.46-101.73) | Impaired healing | 4 | 2.71(1.01-7.21) |
| Haemorrhagic diathesis | 216 | 20.01(17.4-23) | Adrenal insufficiency | 96 | 6.74(5.52-8.25) | Onychoclasis | 4 | 10.94(4.1-29.17) |
| Blood sodium decreased | 213 | 3.99(3.48-4.57) | Labelled drug-drug interaction medication error | 95 | 7.2(5.88-8.82) | Lymphocytosis | 4 | 42.16(15.79-112.56) |
| Basal cell carcinoma | 212 | 3.99(3.48-4.57) | Squamous cell carcinoma | 93 | 8.7(7.09-10.67) | Sinusitis fungal | 4 | 104.07(38.9-278.4) |
| Tumour lysis syndrome | 211 | 7.26(6.33-8.33) | Hepatic failure | 90 | 3.04(2.47-3.74) | Paronychia | 4 | 18.98(7.11-50.63) |
| Heart rate irregular | 210 | 3.13(2.73-3.59) | Eosinophilia | 89 | 4.67(3.79-5.76) | Mucormycosis | 4 | 26.41(9.9-70.48) |
| Pneumonitis | 207 | 2.22(1.93-2.54) | Drug eruption | 88 | 4.74(3.84-5.85) | Pneumonia bacterial | 4 | 8.87(3.32-23.65) |
| Transfusion | 206 | 5.16(4.5-5.93) | Graft versus host disease | 87 | 12.3(9.95-15.21) | Candida infection | 4 | 4.22(1.58-11.24) |
| Blood iron decreased | 201 | 4.84(4.21-5.57) | Drug level above therapeutic | 86 | 17.22(13.9-21.33) | Basal cell carcinoma | 4 | 5.17(1.94-13.79) |
| Blood pressure abnormal | 200 | 2.81(2.45-3.24) | Agranulocytosis | 85 | 4.21(3.4-5.22) | Device related infection | 4 | 5.16(1.94-13.76) |
| Leukocytosis | 196 | 3.74(3.25-4.31) | Hyperbilirubinaemia | 84 | 7.37(5.95-9.14) | Pneumomediastinum | 4 | 48.3(18.09-128.99) |
| Lymphocyte count increased | 196 | 19.22(16.61-22.25) | Encephalopathy | 83 | 3.15(2.54-3.91) | Urinary incontinence | 4 | 3.06(1.15-8.17) |
| Atrial flutter | 195 | 8.36(7.24-9.64) | Photophobia | 82 | 3.97(3.19-4.93) | White blood cell count abnormal | 4 | 11.83(4.44-31.57) |
| Blood potassium decreased | 195 | 2.09(1.82-2.41) | Ventricular tachycardia | 80 | 5.11(4.1-6.37) | Cytomegalovirus infection | 4 | 5.02(1.88-13.39) |
| Spinal operation | 194 | 4.28(3.71-4.93) | Nephropathy toxic | 80 | 6.44(5.17-8.03) | Central nervous system lesion | 4 | 5.81(2.18-15.49) |
| Mantle cell lymphoma | 194 | 44.37(38.02-51.79) | Liver function test increased | 80 | 2.66(2.13-3.31) | Labelled drug-drug interaction medication error | 4 | 7.23(2.71-19.28) |
| Blood blister | 187 | 19.63(16.9-22.8) | Cardiotoxicity | 79 | 7.34(5.88-9.16) | Acute respiratory distress syndrome | 4 | 5.42(2.03-14.45) |
| Spinal fracture | 185 | 2.75(2.38-3.18) | Dermatitis bullous | 79 | 10.63(8.51-13.28) | Respiratory tract congestion | 4 | 5.16(1.94-13.77) |
| Hip arthroplasty | 176 | 3.45(2.98-4.01) | Disorganised speech | 79 | 33.57(26.77-42.1) | Respiratory tract infection | 4 | 3.07(1.15-8.19) |
| Deafness | 175 | 2(1.73-2.32) | Hepatitis | 78 | 3.05(2.44-3.81) | Pneumocystis jirovecii pneumonia | 4 | 6.91(2.59-18.41) |
| Tooth extraction | 175 | 5.04(4.34-5.86) | Jaundice | 76 | 3.03(2.42-3.79) | Lymphocyte count increased | 4 | 25.25(9.46-67.37) |
| Skin fissures | 170 | 2.51(2.16-2.92) | Stevens-johnson syndrome | 76 | 4.12(3.29-5.17) | Post procedural complication | 4 | 5.41(2.03-14.43) |
| Haemorrhage intracranial | 166 | 3.35(2.88-3.91) | Blood bilirubin increased | 76 | 2.98(2.38-3.73) | Blood lactate dehydrogenase increased | 4 | 6.92(2.6-18.46) |
| Platelet count abnormal | 160 | 7.41(6.33-8.67) | Fusarium infection | 73 | 102.09(79.72-130.75) | Neutrophil count increased | 4 | 8.1(3.04-21.61) |
| Pulmonary mass | 159 | 2.85(2.44-3.33) | Toxic skin eruption | 73 | 7.08(5.62-8.92) | Myositis | 3 | 7.82(2.52-24.27) |
| Richter's syndrome | 158 | 51.82(43.56-61.65) | Inappropriate antidiuretic hormone secretion | 69 | 6.7(5.28-8.49) | Ulcer | 3 | 4.19(1.35-13.01) |
| Mass | 154 | 3.18(2.71-3.73) | Cushing's syndrome | 68 | 15.14(11.9-19.26) | Neoplasm | 3 | 5.21(1.68-16.17) |
| Lymphoma | 153 | 3.02(2.58-3.54) | Hypercalcaemia | 67 | 4.92(3.87-6.25) | Lichenification | 3 | 166.02(53.18-518.24) |
| Ecchymosis | 152 | 7.98(6.79-9.39) | Toxic epidermal necrolysis | 66 | 4.33(3.4-5.51) | Cryptococcosis | 3 | 37.97(12.22-117.96) |
| Rash papular | 147 | 2.02(1.72-2.37) | Mouth ulceration | 65 | 2.78(2.18-3.55) | Cholecystitis | 3 | 6.33(2.04-19.65) |
| Gingival bleeding | 147 | 3.28(2.78-3.86) | Respiratory distress | 65 | 2.31(1.81-2.95) | Skin haemorrhage | 3 | 4.04(1.3-12.52) |
| Rib fracture | 145 | 2.15(1.83-2.53) | Fixed eruption | 64 | 26.12(20.34-33.54) | Gastric ulcer | 3 | 3.65(1.18-11.33) |
| Haemoglobin abnormal | 145 | 6.48(5.49-7.64) | Nervous system disorder | 64 | 2.81(2.2-3.59) | Abdominal mass | 3 | 24.84(8-77.14) |
| Sinus disorder | 144 | 2.06(1.75-2.43) | Immunosuppressant drug level increased | 63 | 16.61(12.94-21.34) | Splenomegaly | 3 | 5.59(1.8-17.34) |
| Knee operation | 144 | 4.32(3.66-5.1) | Acute respiratory distress syndrome | 63 | 3.56(2.78-4.56) | Bacteraemia | 3 | 5.39(1.74-16.72) |
| Eye operation | 141 | 9.41(7.95-11.14) | Shock | 62 | 2.74(2.13-3.51) | Skin mass | 3 | 8.38(2.7-26.02) |
| Macular degeneration | 141 | 3.69(3.12-4.35) | Fungaemia | 62 | 37.95(29.37-49.04) | Muscle atrophy | 3 | 5.44(1.75-16.87) |
| Haemothorax | 140 | 15.91(13.4-18.89) | Symmetrical drug-related intertriginous and flexural exanthema | 62 | 59.65(45.97-77.41) | Urethral disorder | 3 | 161.88(51.87-505.26) |
| Blood test abnormal | 135 | 2.61(2.2-3.09) | Pericardial effusion | 60 | 2.4(1.86-3.09) | Tumour lysis syndrome | 3 | 6.99(2.25-21.68) |
| Sars-cov-2 test positive | 135 | 2.3(1.94-2.73) | Acute hepatic failure | 60 | 3.84(2.98-4.95) | Haemorrhage intracranial | 3 | 4.17(1.35-12.95) |
| Glaucoma | 134 | 2.11(1.78-2.5) | Systemic candida | 58 | 21.56(16.59-28.01) | Cerebral aspergillosis | 3 | 99.32(31.9-309.27) |
| Neoplasm | 133 | 3.35(2.82-3.98) | Mental status changes | 58 | 2.21(1.71-2.86) | Haemolytic anaemia | 3 | 8.08(2.6-25.08) |
| Rehabilitation therapy | 133 | 8.82(7.41-10.49) | Pancreatitis acute | 57 | 2.51(1.94-3.26) | Platelet count abnormal | 3 | 9.4(3.03-29.18) |
| Covid-19 pneumonia | 132 | 2.59(2.18-3.08) | Cerebral aspergillosis | 55 | 85.23(64.31-112.97) | Lung infiltration | 3 | 10.41(3.35-32.3) |
| Squamous cell carcinoma | 129 | 4.18(3.51-4.97) | Generalised tonic-clonic seizure | 55 | 2.26(1.73-2.94) | Richter's syndrome | 3 | 55.75(17.93-173.3) |
| Hair texture abnormal | 128 | 2.33(1.96-2.77) | Posterior reversible encephalopathy syndrome | 55 | 4.5(3.45-5.87) | Bacterial infection | 3 | 3.52(1.13-10.91) |
| Internal haemorrhage | 126 | 2.57(2.16-3.07) | Hallucination, auditory | 54 | 3.27(2.51-4.28) | Platelet count increased | 3 | 4.85(1.56-15.06) |
| Ear haemorrhage | 125 | 19.73(16.43-23.7) | Hypertransaminasaemia | 54 | 6.3(4.82-8.24) | Acute myeloid leukaemia | 3 | 4.3(1.39-13.35) |
| Skin infection | 124 | 3.19(2.68-3.81) | Hypomagnesaemia | 52 | 3.27(2.49-4.29) | Lymphocyte count decreased | 3 | 3.13(1.01-9.72) |
| Cardiac pacemaker insertion | 124 | 7.1(5.94-8.5) | Coagulopathy | 52 | 3.03(2.31-3.98) | International normalised ratio increased | 3 | 3.22(1.04-9.98) |
| Nodule | 122 | 2.66(2.23-3.18) | Labelled drug-drug interaction issue | 52 | 32.17(24.34-42.51) | Diffuse large b-cell lymphoma | 3 | 7.62(2.45-23.64) |
| Cholecystectomy | 119 | 3.77(3.14-4.52) | Erythema multiforme | 51 | 5.66(4.29-7.45) | Blood immunoglobulin m increased | 3 | 110.68(35.53-344.79) |
| Pericarditis | 118 | 2.06(1.72-2.48) | Blood creatine phosphokinase increased | 51 | 2.06(1.56-2.71) | Pharyngitis streptococcal | 3 | 5.51(1.77-17.09) |
| Malignant melanoma | 117 | 2.5(2.09-3) | Bowen's disease | 50 | 33.68(25.34-44.77) | Supraventricular tachycardia | 3 | 7.72(2.49-23.94) |
| Cardiac operation | 116 | 4.26(3.55-5.12) | Long qt syndrome | 50 | 17.29(13.06-22.91) | Pulmonary alveolar haemorrhage | 3 | 11.29(3.64-35.03) |
| Uveitis | 114 | 2.37(1.97-2.85) | Toxic optic neuropathy | 49 | 98.81(73.1-133.54) |  |  |  |
| Arthropod bite | 113 | 4.7(3.9-5.67) | Face oedema | 48 | 2.89(2.18-3.84) |  |  |  |
| Blood immunoglobulin m increased | 113 | 81.37(65.55-101.02) | Bone marrow failure | 48 | 2.07(1.56-2.75) |  |  |  |
| Purpura | 111 | 4.65(3.85-5.61) | Myelodysplastic syndrome | 47 | 3.11(2.33-4.14) |  |  |  |
| Prostatic operation | 110 | 31.96(26.16-39.05) | Scedosporium infection | 47 | 60.59(44.91-81.74) |  |  |  |
| Skin atrophy | 108 | 5.6(4.63-6.78) | Basal cell carcinoma | 46 | 2.48(1.85-3.31) |  |  |  |
| Ventricular tachycardia | 108 | 2.38(1.97-2.88) | Pulmonary mass | 45 | 2.32(1.73-3.11) |  |  |  |
| Prostatomegaly | 106 | 8.11(6.68-9.85) | Cheilitis | 45 | 8.59(6.4-11.53) |  |  |  |
| Sudden death | 104 | 4.06(3.34-4.93) | Dysphoria | 44 | 6.66(4.95-8.97) |  |  |  |
| Oral surgery | 104 | 14.63(11.99-17.84) | Immune reconstitution inflammatory syndrome | 44 | 6.28(4.67-8.46) |  |  |  |
| Mouth haemorrhage | 104 | 4.48(3.69-5.43) | Potentiating drug interaction | 43 | 11.62(8.6-15.72) |  |  |  |
| Autoimmune haemolytic anaemia | 104 | 7.96(6.54-9.68) | Photopsia | 41 | 6.09(4.47-8.28) |  |  |  |
| Haemorrhage subcutaneous | 103 | 8.95(7.35-10.91) | Ileus paralytic | 41 | 10.06(7.39-13.7) |  |  |  |
| Cytopenia | 102 | 2.17(1.78-2.64) | Brain abscess | 40 | 13.77(10.07-18.84) |  |  |  |
| Chemotherapy | 100 | 7.87(6.44-9.6) | Pneumothorax | 40 | 2.19(1.61-2.99) |  |  |  |
| Blood uric acid increased | 99 | 5.66(4.64-6.91) | Hepatitis cholestatic | 40 | 7.18(5.26-9.81) |  |  |  |
| Immune system disorder | 98 | 2.11(1.73-2.57) | Oral candidiasis | 38 | 2.71(1.97-3.72) |  |  |  |
| Compression fracture | 95 | 2.21(1.8-2.7) | Enterococcal infection | 38 | 7.66(5.56-10.55) |  |  |  |
| Squamous cell carcinoma of skin | 95 | 4.49(3.66-5.5) | Hypophosphataemia | 38 | 4.52(3.28-6.22) |  |  |  |
| Skin mass | 94 | 3.82(3.11-4.68) | Electrolyte imbalance | 38 | 3.05(2.22-4.19) |  |  |  |
| Hypogammaglobulinaemia | 94 | 4.38(3.57-5.38) | Inhibitory drug interaction | 38 | 20.38(14.75-28.15) |  |  |  |
| Cardiomyopathy | 93 | 2.22(1.81-2.72) | Myositis | 37 | 4.02(2.91-5.56) |  |  |  |
| Stent placement | 92 | 3.93(3.2-4.83) | Dyschromatopsia | 37 | 33.73(24.23-46.96) |  |  |  |
| Limb operation | 90 | 5.45(4.42-6.71) | Halo vision | 37 | 32.73(23.52-45.56) |  |  |  |
| Cardiotoxicity | 90 | 2.88(2.34-3.55) | Ventricular fibrillation | 37 | 3.65(2.64-5.04) |  |  |  |
| Gastric haemorrhage | 90 | 2.55(2.07-3.14) | Disseminated intravascular coagulation | 37 | 2.96(2.14-4.08) |  |  |  |
| Platelet count increased | 89 | 2.08(1.69-2.56) | Antibiotic level below therapeutic | 37 | 495.2(321.86-761.88) |  |  |  |
| Wound haemorrhage | 86 | 6.67(5.38-8.27) | Acute generalised exanthematous pustulosis | 37 | 4.23(3.06-5.85) |  |  |  |
| Dental operation | 85 | 11.02(8.86-13.71) | Myoclonus | 36 | 2.98(2.15-4.14) |  |  |  |
| Coronary artery occlusion | 85 | 2.52(2.03-3.12) | Xanthopsia | 36 | 134.79(94.04-193.21) |  |  |  |
| Scratch | 84 | 2.38(1.92-2.95) | Cryptococcosis | 36 | 19.44(13.95-27.08) |  |  |  |
| Lymphoedema | 84 | 3.6(2.9-4.46) | Endophthalmitis | 36 | 5.07(3.66-7.04) |  |  |  |
| Aspergillus infection | 83 | 3.17(2.55-3.94) | Hallucinations, mixed | 35 | 7.79(5.58-10.87) |  |  |  |
| Ventricular fibrillation | 82 | 2.8(2.25-3.49) | Mixed liver injury | 35 | 10.02(7.18-14) |  |  |  |
| Blood immunoglobulin m decreased | 82 | 25.03(19.91-31.46) | Klebsiella infection | 35 | 6.23(4.47-8.7) |  |  |  |
| Haemolytic anaemia | 81 | 3.16(2.54-3.94) | Ileus | 34 | 2.78(1.99-3.89) |  |  |  |
| Supraventricular tachycardia | 81 | 3.02(2.42-3.76) | Tachypnoea | 34 | 2.31(1.65-3.24) |  |  |  |
| Scab | 80 | 2.04(1.63-2.54) | Histoplasmosis | 34 | 18.55(13.19-26.09) |  |  |  |
| Eye infection | 80 | 2(1.61-2.5) | Pseudomonas infection | 34 | 3.78(2.7-5.3) |  |  |  |
| Hip surgery | 79 | 4.32(3.46-5.4) | Meningitis cryptococcal | 33 | 17.59(12.45-24.86) |  |  |  |
| Cardiac flutter | 78 | 3.81(3.05-4.77) | Pneumonia bacterial | 32 | 2.95(2.09-4.18) |  |  |  |
| Brain neoplasm | 77 | 2.51(2.01-3.14) | Hepatic encephalopathy | 32 | 3.13(2.21-4.43) |  |  |  |
| Procedural haemorrhage | 77 | 5.64(4.5-7.07) | Sinus bradycardia | 31 | 3.14(2.21-4.47) |  |  |  |
| Tooth fracture | 75 | 2.04(1.62-2.56) | Primary adrenal insufficiency | 31 | 102.09(69.84-149.22) |  |  |  |
| Subarachnoid haemorrhage | 75 | 2.26(1.8-2.84) | Brain oedema | 30 | 2.33(1.62-3.33) |  |  |  |
| Gallbladder operation | 75 | 6.34(5.04-7.98) | Petechiae | 30 | 2.79(1.95-3.99) |  |  |  |
| Urinary bladder haemorrhage | 75 | 11.02(8.74-13.9) | Leukaemia recurrent | 30 | 17.22(11.98-24.74) |  |  |  |
| Pelvic fracture | 74 | 2.89(2.3-3.63) | Dermatitis allergic | 30 | 2.09(1.46-2.99) |  |  |  |
| Onychomycosis | 72 | 5.14(4.07-6.49) | Oral mucosal blistering | 30 | 3.74(2.61-5.35) |  |  |  |
| Nail growth abnormal | 72 | 19.95(15.67-25.4) | Transplant rejection | 30 | 3.3(2.31-4.73) |  |  |  |
| Prostatic disorder | 70 | 3.42(2.7-4.33) | Clostridium difficile colitis | 30 | 2.53(1.77-3.62) |  |  |  |
| Blood immunoglobulin g decreased | 70 | 8.41(6.62-10.68) | Disseminated mucormycosis | 30 | 69.89(47.92-101.91) |  |  |  |
| Blood disorder | 69 | 2.69(2.12-3.41) | Sunburn | 29 | 3.39(2.35-4.88) |  |  |  |
| Muscle strain | 69 | 2.4(1.89-3.04) | Necrosis | 29 | 5.42(3.76-7.82) |  |  |  |
| Leukaemia | 68 | 2.09(1.64-2.65) | Malabsorption | 29 | 4.55(3.15-6.55) |  |  |  |
| Ingrowing nail | 68 | 8.74(6.86-11.14) | Multiple-drug resistance | 29 | 7.03(4.87-10.14) |  |  |  |
| Oral mucosal blistering | 68 | 2.94(2.32-3.74) | Blood lactate dehydrogenase increased | 29 | 2.09(1.45-3) |  |  |  |
| Diffuse large b-cell lymphoma | 68 | 2.49(1.96-3.17) | Haemophagocytic lymphohistiocytosis | 29 | 2.37(1.64-3.41) |  |  |  |
| Bronchopulmonary aspergillosis | 68 | 2.66(2.09-3.38) | Fluoride increased | 28 | 1100.67(595.49-2034.42) |  |  |  |
| Bladder disorder | 67 | 2.13(1.67-2.71) | Disseminated aspergillosis | 28 | 46.71(31.82-68.58) |  |  |  |
| Blood calcium increased | 65 | 2.31(1.81-2.95) | Cross sensitivity reaction | 28 | 11.25(7.74-16.34) |  |  |  |
| Heart rate abnormal | 65 | 3.42(2.68-4.37) | Ataxia | 27 | 2.14(1.47-3.12) |  |  |  |
| Cardiac tamponade | 64 | 4.31(3.37-5.52) | Bacteraemia | 27 | 2.02(1.38-2.94) |  |  |  |
| Retinal haemorrhage | 64 | 3.26(2.55-4.17) | Eyelid oedema | 27 | 2.37(1.62-3.46) |  |  |  |
| Pulmonary haemorrhage | 64 | 2.81(2.19-3.59) | Pulmonary mucormycosis | 27 | 41.72(28.26-61.6) |  |  |  |
| Inguinal hernia | 63 | 3.03(2.37-3.89) | Adrenal suppression | 27 | 32.16(21.85-47.35) |  |  |  |
| Nerve compression | 63 | 2(1.56-2.57) | Haemodynamic instability | 27 | 3.42(2.34-4.99) |  |  |  |
| Post procedural infection | 62 | 2.14(1.67-2.75) | Fungal endocarditis | 26 | 86.52(57.42-130.38) |  |  |  |
| Blood magnesium decreased | 62 | 2.09(1.63-2.69) | Neurological symptom | 26 | 4.34(2.95-6.39) |  |  |  |
| Polyp | 59 | 2.69(2.08-3.48) | Dermatitis exfoliative | 26 | 7.37(5.01-10.85) |  |  |  |
| Vascular rupture | 59 | 12.45(9.58-16.18) | Lung infiltration | 26 | 3.76(2.56-5.53) |  |  |  |
| Pneumonia fungal | 58 | 3.82(2.94-4.95) | Myopathy | 25 | 2.86(1.93-4.24) |  |  |  |
| Spinal stenosis | 58 | 2.04(1.58-2.64) | Lip blister | 25 | 8.24(5.56-12.23) |  |  |  |
| Micrographic skin surgery | 58 | 118.57(86.09-163.31) | Skin hyperpigmentation | 25 | 2.44(1.65-3.61) |  |  |  |
| Red blood cell count increased | 58 | 6.09(4.69-7.91) | Pulmonary haemorrhage | 25 | 3.16(2.13-4.68) |  |  |  |
| Splenic rupture | 57 | 11.32(8.67-14.78) | Visual field defect | 25 | 3.51(2.37-5.2) |  |  |  |
| Aortic aneurysm | 56 | 2.18(1.68-2.84) | Cholestatic liver injury | 25 | 9.81(6.61-14.57) |  |  |  |
| Haemorrhagic stroke | 56 | 2.1(1.62-2.74) | Generalised oedema | 25 | 2.05(1.38-3.03) |  |  |  |
| Waldenstrom's macroglobulinaemia | 56 | 34.57(26.08-45.84) | Pulmonary alveolar haemorrhage | 25 | 3.92(2.65-5.81) |  |  |  |
| Mantle cell lymphoma recurrent | 56 | 38.77(29.17-51.52) | Graft versus host disease in gastrointestinal tract | 25 | 8.06(5.43-11.96) |  |  |  |
| Red blood cell count abnormal | 55 | 10.7(8.16-14.03) | Fluorosis | 24 | 431.26(256.52-725.03) |  |  |  |
| Dental care | 54 | 7.89(6.01-10.35) | Sinusitis fungal | 24 | 26.81(17.82-40.34) |  |  |  |
| Onychomadesis | 54 | 4.02(3.07-5.26) | Tumour lysis syndrome | 24 | 2.33(1.56-3.47) |  |  |  |
| Catheterisation cardiac | 54 | 3.73(2.85-4.88) | Fungal skin infection | 24 | 4.98(3.33-7.44) |  |  |  |
| Investigation abnormal | 54 | 11.29(8.59-14.85) | Product solubility abnormal | 24 | 3.61(2.42-5.39) |  |  |  |
| Renal cyst | 53 | 2.15(1.64-2.82) | Peripheral sensory neuropathy | 24 | 3.62(2.43-5.41) |  |  |  |
| Pericardial haemorrhage | 53 | 7.47(5.68-9.83) | Phaeohyphomycosis | 23 | 34.77(22.85-52.91) |  |  |  |
| Stem cell transplant | 53 | 10.03(7.62-13.21) | Metabolic encephalopathy | 23 | 7.19(4.76-10.84) |  |  |  |
| Ventricular extrasystoles | 53 | 2.03(1.55-2.66) | Coccidioidomycosis | 23 | 17.84(11.78-27) |  |  |  |
| Immunoglobulins decreased | 52 | 10.81(8.18-14.29) | Anuria | 22 | 2.62(1.72-3.98) |  |  |  |
| Coronary arterial stent insertion | 52 | 3.06(2.32-4.02) | Chloropsia | 22 | 206.5(127.57-334.29) |  |  |  |
| Neck mass | 51 | 5.32(4.03-7.02) | Cystitis haemorrhagic | 22 | 4.74(3.12-7.21) |  |  |  |
| Shoulder operation | 51 | 3.5(2.65-4.61) | Acute myeloid leukaemia recurrent | 22 | 9.25(6.07-14.1) |  |  |  |
| Bleeding time prolonged | 51 | 16.13(12.14-21.44) | Blood magnesium decreased | 22 | 2.14(1.41-3.25) |  |  |  |
| Sinus operation | 50 | 6.18(4.67-8.19) | Rash morbilliform | 21 | 7.18(4.67-11.04) |  |  |  |
| Subdural haemorrhage | 50 | 5.54(4.18-7.33) | Oesophageal candidiasis | 21 | 5.02(3.27-7.71) |  |  |  |
| Renal function test abnormal | 50 | 3.13(2.37-4.14) | Escherichia infection | 21 | 2.39(1.56-3.67) |  |  |  |
| Deafness unilateral | 49 | 2.95(2.23-3.91) | Ventricular extrasystoles | 21 | 2.32(1.51-3.56) |  |  |  |
| Protein total decreased | 49 | 4.89(3.68-6.49) | Hepatitis acute | 20 | 3.06(1.97-4.74) |  |  |  |
| Haemorrhage urinary tract | 49 | 5.64(4.24-7.48) | Facial pain | 20 | 2.03(1.31-3.15) |  |  |  |
| Medical device implantation | 49 | 10.32(7.75-13.74) | Mouth swelling | 20 | 2.39(1.54-3.7) |  |  |  |
| Bone marrow transplant | 47 | 9.26(6.91-12.4) | Cutaneous vasculitis | 20 | 5.02(3.23-7.8) |  |  |  |
| Conjunctival haemorrhage | 47 | 3.99(2.99-5.33) | Shock haemorrhagic | 20 | 2.08(1.34-3.22) |  |  |  |
| Angina bullosa haemorrhagica | 47 | 56.58(41.04-78) | Toxic encephalopathy | 20 | 3.4(2.19-5.28) |  |  |  |
| Arterial occlusive disease | 47 | 2.17(1.63-2.89) | Respiratory acidosis | 20 | 4.77(3.07-7.41) |  |  |  |
| Synovial cyst | 46 | 2.89(2.16-3.86) | Immunosuppressant drug level decreased | 20 | 15.01(9.63-23.39) |  |  |  |
| Colonoscopy | 46 | 6.12(4.57-8.21) | Neurological decompensation | 20 | 7.68(4.94-11.93) |  |  |  |
| Food poisoning | 46 | 2.54(1.9-3.39) | Jarisch-herxheimer reaction | 20 | 22.07(14.13-34.47) |  |  |  |
| Ulcer haemorrhage | 46 | 2.08(1.56-2.79) | Cardiovascular insufficiency | 20 | 11.61(7.46-18.07) |  |  |  |
| White blood cell disorder | 46 | 4.86(3.63-6.51) | Ventricular arrhythmia | 19 | 4.71(3-7.4) |  |  |  |
| Energy increased | 45 | 2.28(1.7-3.06) | Geotrichum infection | 19 | 72.86(45.31-117.16) |  |  |  |
| Skin abrasion | 45 | 2.08(1.55-2.79) | Anticoagulation drug level increased | 19 | 40.78(25.64-64.86) |  |  |  |
| Central venous catheterisation | 45 | 4.05(3.02-5.44) | Left ventricular dysfunction | 19 | 2.6(1.66-4.08) |  |  |  |
| Benign prostatic hyperplasia | 45 | 2.81(2.09-3.77) | Hypertrophic osteoarthropathy | 19 | 385.44(217.73-682.32) |  |  |  |
| Hyperuricaemia | 44 | 3.62(2.69-4.88) | Dermatitis exfoliative generalised | 19 | 4.05(2.58-6.37) |  |  |  |
| Lymph node pain | 44 | 7.47(5.53-10.08) | Cachexia | 18 | 3.28(2.06-5.21) |  |  |  |
| Cardiac murmur | 43 | 2.03(1.5-2.74) | Chromatopsia | 18 | 33.1(20.6-53.17) |  |  |  |
| Vitreous haemorrhage | 43 | 5.16(3.81-6.98) | Ecchymosis | 18 | 2.66(1.67-4.22) |  |  |  |
| Haemoglobin increased | 43 | 2.92(2.16-3.94) | Oral disorder | 18 | 2.18(1.37-3.47) |  |  |  |
| Chronic lymphocytic leukaemia recurrent | 43 | 18.67(13.67-25.5) | Toxic neuropathy | 18 | 40(24.84-64.41) |  |  |  |
| Erysipelas | 42 | 2.51(1.85-3.4) | Corneal perforation | 18 | 27.95(17.43-44.82) |  |  |  |
| Fluid intake reduced | 42 | 2.8(2.06-3.79) | Trichosporon infection | 18 | 43.7(27.1-70.48) |  |  |  |
| Coronary artery bypass | 42 | 2.66(1.96-3.6) | Retinal haemorrhage | 18 | 2.63(1.66-4.18) |  |  |  |
| Pleurisy | 41 | 2.39(1.76-3.25) | Cardiac failure acute | 18 | 2.29(1.44-3.64) |  |  |  |
| Nail infection | 41 | 8.63(6.32-11.8) | Inflammatory marker increased | 18 | 2.76(1.74-4.38) |  |  |  |
| Cheilitis | 41 | 2.69(1.98-3.67) | Aplastic anaemia | 17 | 3.03(1.88-4.89) |  |  |  |
| Precancerous skin lesion | 41 | 7.79(5.71-10.64) | Cushingoid | 17 | 4.7(2.92-7.58) |  |  |  |
| Nail discolouration | 41 | 3.02(2.22-4.11) | Porphyria acute | 17 | 10.77(6.67-17.39) |  |  |  |
| Lumbar vertebral fracture | 41 | 2.69(1.98-3.67) | Bacterial sepsis | 17 | 5.05(3.13-8.14) |  |  |  |
| Middle ear effusion | 40 | 5.44(3.98-7.45) | Lung opacity | 17 | 5.02(3.11-8.09) |  |  |  |
| Intestinal haemorrhage | 40 | 2.23(1.63-3.04) | Hepatic enzyme abnormal | 17 | 2.93(1.82-4.72) |  |  |  |
| Supraventricular extrasystoles | 40 | 4.86(3.56-6.65) | Reaction to excipient | 17 | 3.11(1.93-5.01) |  |  |  |
| Non-hodgkin's lymphoma | 39 | 2.14(1.56-2.94) | Tongue blistering | 17 | 7.18(4.45-11.59) |  |  |  |
| Bladder operation | 39 | 10.71(7.77-14.77) | Oral mucosa erosion | 17 | 13.07(8.08-21.13) |  |  |  |
| Blood viscosity decreased | 39 | 29.97(21.44-41.89) | Lung transplant rejection | 17 | 25.15(15.49-40.85) |  |  |  |
| Biopsy | 38 | 13.52(9.74-18.76) | Bilirubin conjugated increased | 17 | 8.36(5.18-13.49) |  |  |  |
| Ventricular arrhythmia | 38 | 3.27(2.38-4.51) | Venoocclusive liver disease | 17 | 3.03(1.88-4.88) |  |  |  |
| Platelet disorder | 38 | 4.38(3.18-6.04) | Acute graft versus host disease in skin | 17 | 6.31(3.91-10.17) |  |  |  |
| Muscle haemorrhage | 38 | 3.22(2.34-4.44) | Bronchopulmonary aspergillosis allergic | 17 | 14.49(8.96-23.43) |  |  |  |
| Malignant pleural effusion | 38 | 7.36(5.33-10.18) | Azotaemia | 16 | 4.32(2.64-7.06) |  |  |  |
| Paranasal sinus hypersecretion | 38 | 3.08(2.24-4.25) | Enthesopathy | 16 | 7.2(4.4-11.78) |  |  |  |
| Intensive care | 37 | 7.28(5.25-10.1) | Granuloma | 16 | 4.63(2.83-7.56) |  |  |  |
| Neutrophil count abnormal | 37 | 3.83(2.77-5.3) | Cell death | 16 | 6.9(4.21-11.29) |  |  |  |
| Foot operation | 36 | 2.09(1.5-2.9) | Bone marrow transplant | 16 | 8.9(5.43-14.57) |  |  |  |
| Cardiac ablation | 36 | 5.83(4.19-8.12) | Periorbital oedema | 16 | 2.98(1.83-4.88) |  |  |  |
| Renal mass | 36 | 5.24(3.76-7.29) | Capillary leak syndrome | 16 | 7.54(4.6-12.34) |  |  |  |
| Recurrent cancer | 36 | 2.44(1.76-3.39) | Acinetobacter infection | 16 | 14.65(8.92-24.04) |  |  |  |
| Melanocytic naevus | 36 | 2.32(1.67-3.22) | Tracheobronchitis | 16 | 21.23(12.9-34.94) |  |  |  |
| Cardiac valve disease | 36 | 2.2(1.58-3.05) | Cholecystitis acute | 16 | 2.96(1.81-4.84) |  |  |  |
| Eye contusion | 35 | 3.7(2.65-5.17) | Intracranial pressure increased | 16 | 2.68(1.64-4.38) |  |  |  |
| Paronychia | 35 | 2.4(1.72-3.34) | Brain natriuretic peptide increased | 16 | 4.9(3-8.02) |  |  |  |
| Lymphocyte count abnormal | 35 | 8.61(6.14-12.07) | Vulvovaginal burning sensation | 16 | 2.07(1.27-3.39) |  |  |  |
| Post procedural haematoma | 35 | 7.12(5.08-9.97) | Chapped lips | 15 | 3.25(1.96-5.41) |  |  |  |
| Umbilical hernia | 34 | 2.12(1.51-2.97) | Skin toxicity | 15 | 2.29(1.38-3.8) |  |  |  |
| Pneumonia viral | 34 | 3.05(2.17-4.27) | Herpes simplex | 15 | 2.61(1.57-4.34) |  |  |  |
| Failure to thrive | 34 | 2.39(1.71-3.35) | Fungal sepsis | 15 | 19.57(11.71-32.72) |  |  |  |
| Central nervous system lymphoma | 34 | 7.42(5.27-10.44) | Aspergilloma | 15 | 47.88(28.32-80.94) |  |  |  |
| Poor peripheral circulation | 34 | 2.54(1.81-3.57) | Photodermatosis | 15 | 38.34(22.77-64.58) |  |  |  |
| Nasal ulcer | 33 | 6.3(4.46-8.91) | Acute pulmonary oedema | 15 | 2.83(1.71-4.71) |  |  |  |
| Iron deficiency | 33 | 2.11(1.5-2.98) | Nephrotic syndrome | 15 | 2.11(1.27-3.5) |  |  |  |
| Cystoid macular oedema | 33 | 4.99(3.53-7.04) | Tongue ulceration | 15 | 4.16(2.5-6.9) |  |  |  |
| Post procedural contusion | 33 | 28.6(19.89-41.12) | Infective aneurysm | 15 | 22.67(13.55-37.95) |  |  |  |
| Haemorrhoidal haemorrhage | 33 | 2(1.42-2.81) | Mycotic endophthalmitis | 15 | 66.9(39.28-113.92) |  |  |  |
| Transplant | 32 | 3.86(2.72-5.47) | Persecutory delusion | 15 | 5.86(3.52-9.74) |  |  |  |
| Sinus node dysfunction | 32 | 3.87(2.73-5.49) | Myocardial necrosis marker increased | 15 | 6.58(3.95-10.94) |  |  |  |
| Cerebral aspergillosis | 32 | 16.16(11.28-23.15) | Gastrointestinal toxicity | 15 | 2.73(1.64-4.54) |  |  |  |
| Hepatitis b reactivation | 32 | 2.38(1.68-3.38) | Cerebral fungal infection | 15 | 52.11(30.78-88.24) |  |  |  |
| Urinary tract disorder | 32 | 2.14(1.51-3.03) | Superficial inflammatory dermatosis | 15 | 33.33(19.83-56.03) |  |  |  |
| Pyoderma gangrenosum | 32 | 2.61(1.84-3.7) | Pulmonary function test decreased | 15 | 2.64(1.59-4.38) |  |  |  |
| Heart valve replacement | 32 | 6.72(4.73-9.55) | Macule | 14 | 3.69(2.18-6.24) |  |  |  |
| Fungal skin infection | 32 | 2.29(1.62-3.25) | Oliguria | 14 | 2.32(1.37-3.92) |  |  |  |
| Bacterial sepsis | 31 | 3.19(2.24-4.55) | Keratitis fungal | 14 | 66.19(38.16-114.82) |  |  |  |
| Haemorrhagic disorder | 31 | 9.02(6.29-12.91) | Lung transplant | 14 | 2.92(1.73-4.94) |  |  |  |
| Cerebral haematoma | 31 | 2.86(2.01-4.08) | Organ failure | 14 | 4.34(2.57-7.34) |  |  |  |
| Craniofacial fracture | 31 | 2.18(1.53-3.1) | Tongue oedema | 14 | 3.38(2-5.71) |  |  |  |
| Sudden cardiac death | 31 | 3.96(2.78-5.65) | Skin necrosis | 14 | 2.6(1.54-4.39) |  |  |  |
| Nasal operation | 30 | 12.95(8.96-18.72) | Lip oedema | 14 | 2.77(1.64-4.68) |  |  |  |
| Appendicectomy | 30 | 2.71(1.89-3.88) | Pseudohyponatraemia | 14 | 88.04(50.32-154.02) |  |  |  |
| Haematotympanum | 30 | 158.54(99-253.89) | Left ventricular failure | 14 | 4.12(2.43-6.96) |  |  |  |
| Skin operation | 30 | 30.37(20.73-44.51) | Mycobacterial infection | 14 | 7.16(4.23-12.13) |  |  |  |
| Tongue ulceration | 30 | 2.88(2.01-4.13) | Vulvovaginal pain | 14 | 2.34(1.38-3.95) |  |  |  |
| Blood immunoglobulin m abnormal | 30 | 120.37(77.03-188.09) | Renal tubular acidosis | 14 | 5.84(3.45-9.88) |  |  |  |
| Urinary tract obstruction | 30 | 3.33(2.32-4.77) | Pulmonary cavitation | 14 | 14.92(8.78-25.35) |  |  |  |
| Animal bite | 29 | 3.35(2.32-4.84) | Product ineffective | 14 | 2.57(1.52-4.34) |  |  |  |
| Discouragement | 29 | 3.12(2.16-4.5) | Renal tubular disorder | 14 | 5.2(3.07-8.79) |  |  |  |
| Renal haemorrhage | 29 | 4.26(2.95-6.15) | Systemic inflammatory response syndrome | 14 | 3.34(1.97-5.64) |  |  |  |
| Ear operation | 28 | 25.38(17.16-37.55) | Graft versus host disease in liver | 14 | 15.07(8.87-25.61) |  |  |  |
| Aortic stenosis | 28 | 2.75(1.89-3.99) | Acquired apparent mineralocorticoid excess | 14 | 266.78(142.77-498.5) |  |  |  |
| Traumatic haemorrhage | 28 | 4.15(2.86-6.04) | Rales | 13 | 2.08(1.21-3.59) |  |  |  |
| Blood potassium abnormal | 28 | 3.11(2.14-4.52) | Endocarditis | 13 | 2.33(1.35-4.01) |  |  |  |
| Pneumonia pseudomonal | 28 | 4.31(2.97-6.27) | Transplant | 13 | 4.5(2.61-7.76) |  |  |  |
| Chronic lymphocytic leukaemia refractory | 28 | 106.43(67.71-167.31) | Hyperpyrexia | 13 | 2.8(1.62-4.83) |  |  |  |
| Renal neoplasm | 27 | 3.65(2.5-5.34) | Papilloedema | 13 | 2.74(1.59-4.73) |  |  |  |
| Lymphadenitis | 27 | 3.61(2.47-5.29) | Acute sinusitis | 13 | 7.94(4.59-13.72) |  |  |  |
| Panniculitis | 27 | 3(2.05-4.39) | Rash vesicular | 13 | 2.4(1.39-4.14) |  |  |  |
| Prostatitis | 27 | 2.89(1.98-4.23) | Brain herniation | 13 | 4.33(2.51-7.47) |  |  |  |
| Spleen disorder | 27 | 5.11(3.49-7.49) | Staphylococcal sepsis | 13 | 2.82(1.63-4.86) |  |  |  |
| Iridocyclitis | 27 | 2.59(1.77-3.78) | Failure to thrive | 13 | 2.64(1.53-4.55) |  |  |  |
| Limb mass | 27 | 2.36(1.62-3.45) | Differentiation syndrome | 13 | 10.55(6.1-18.25) |  |  |  |
| Face injury | 27 | 2.32(1.59-3.39) | Oral fungal infection | 13 | 4.38(2.54-7.56) |  |  |  |
| Adverse food reaction | 27 | 7.08(4.83-10.39) | Mucosal ulceration | 13 | 12.83(7.41-22.22) |  |  |  |
| Nail bed bleeding | 27 | 14.55(9.86-21.49) | Jaundice cholestatic | 13 | 3.87(2.24-6.67) |  |  |  |
| Spondylitis | 26 | 2.01(1.36-2.95) | Contraindicated product prescribed | 13 | 4.69(2.72-8.09) |  |  |  |
| Abdominal mass | 26 | 3.12(2.12-4.59) | Lip squamous cell carcinoma | 13 | 62.4(35.29-110.34) |  |  |  |
| Therapeutic procedure | 26 | 5.99(4.05-8.84) | Rhinocerebral mucormycosis | 13 | 27.43(15.74-47.8) |  |  |  |
| Post herpetic neuralgia | 26 | 5.54(3.76-8.18) | Neuroendocrine carcinoma of the skin | 13 | 10.94(6.32-18.94) |  |  |  |
| Non-cardiac chest pain | 26 | 3.22(2.19-4.74) | Graft versus host disease in skin | 13 | 4.39(2.54-7.57) |  |  |  |
| Heart valve incompetence | 26 | 2.79(1.9-4.11) | Growth failure | 12 | 24.42(13.72-43.47) |  |  |  |
| Blood creatinine abnormal | 26 | 2.15(1.46-3.17) | Self-medication | 12 | 3.56(2.02-6.29) |  |  |  |
| Abdominal lymphadenopathy | 26 | 7.73(5.23-11.43) | Liver abscess | 12 | 3.26(1.85-5.75) |  |  |  |
| Arrhythmia supraventricular | 26 | 9.83(6.64-14.56) | Hypernatraemia | 12 | 2.26(1.28-3.99) |  |  |  |
| Carotid artery occlusion | 25 | 2.46(1.66-3.65) | Ototoxicity | 12 | 5.57(3.16-9.84) |  |  |  |
| Splenic haemorrhage | 25 | 15.17(10.11-22.76) | Pneumonia klebsiella | 12 | 5.83(3.3-10.29) |  |  |  |
| Cardiac fibrillation | 25 | 6.07(4.08-9.03) | Haemorrhage subcutaneous | 12 | 2.92(1.66-5.15) |  |  |  |
| Intra-abdominal fluid collection | 25 | 2.98(2.01-4.42) | Drug clearance decreased | 12 | 8.81(4.99-15.58) |  |  |  |
| Implantable defibrillator insertion | 25 | 7.08(4.75-10.55) | Scopulariopsis infection | 12 | 260.2(132.77-509.91) |  |  |  |
| Central nervous system haemorrhage | 25 | 14.14(9.44-21.2) | Communication disorder | 12 | 3(1.7-5.29) |  |  |  |
| Lip pain | 24 | 2.64(1.76-3.95) | Histoplasmosis disseminated | 12 | 6.91(3.91-12.21) |  |  |  |
| Lung operation | 24 | 8.23(5.47-12.37) | Pneumocystis jirovecii infection | 12 | 7.87(4.45-13.9) |  |  |  |
| Bowen's disease | 24 | 5.42(3.61-8.12) | Electrocardiogram st segment depression | 12 | 6.41(3.63-11.32) |  |  |  |
| Shoulder arthroplasty | 24 | 2.54(1.7-3.79) | Disseminated cryptococcosis | 12 | 13.52(7.63-23.96) |  |  |  |
| Mucosal haemorrhage | 24 | 6.75(4.5-10.14) | Gastrointestinal fungal infection | 12 | 20.79(11.7-36.95) |  |  |  |
| Catheter site haemorrhage | 24 | 3.02(2.02-4.51) | Illusion | 11 | 6.11(3.37-11.07) |  |  |  |
| Product blister packaging issue | 24 | 6(4-9.01) | Lentigo | 11 | 21.15(11.6-38.58) |  |  |  |
| Hyphaema | 23 | 14.44(9.47-22.03) | Sinus arrest | 11 | 7.48(4.13-13.55) |  |  |  |
| Multimorbidity | 23 | 5.29(3.5-8) | Hypercapnia | 11 | 3.43(1.9-6.21) |  |  |  |
| Prostatectomy | 23 | 13.69(8.98-20.87) | Septic embolus | 11 | 14.03(7.72-25.5) |  |  |  |
| Dental implantation | 23 | 3.98(2.63-6.01) | Blood blister | 11 | 3.08(1.71-5.58) |  |  |  |
| Oesophageal stenosis | 23 | 2.79(1.85-4.2) | Paraplegia | 11 | 3.61(1.99-6.52) |  |  |  |
| Aortic valve replacement | 23 | 5.61(3.71-8.49) | Pseudoporphyria | 11 | 29.43(16.08-53.88) |  |  |  |
| Pneumonia pneumococcal | 23 | 5.21(3.45-7.88) | Product deposit | 11 | 5.39(2.98-9.76) |  |  |  |
| Traumatic haematoma | 23 | 6.49(4.29-9.82) | Scintillating scotoma | 11 | 54.9(29.64-101.67) |  |  |  |
| Transurethral prostatectomy | 23 | 15.72(10.29-24) | Pseudomembranous colitis | 11 | 5.5(3.04-9.95) |  |  |  |
| Lung neoplasm | 22 | 2.8(1.84-4.26) | Fungal test positive | 11 | 30.88(16.86-56.57) |  |  |  |
| Brain abscess | 22 | 2.58(1.7-3.93) | Neutropenic colitis | 11 | 4.44(2.45-8.03) |  |  |  |
| Eye injury | 22 | 2.18(1.43-3.32) | Tongue discolouration | 11 | 2.35(1.3-4.25) |  |  |  |
| Brain operation | 22 | 2.78(1.83-4.23) | Hepatitis fulminant | 11 | 4.13(2.28-7.48) |  |  |  |
| Tachyarrhythmia | 22 | 3.15(2.07-4.8) | Pneumonia pseudomonal | 11 | 4.86(2.68-8.79) |  |  |  |
| Otitis media | 22 | 2.22(1.46-3.39) | International normalised ratio abnormal | 11 | 3.43(1.89-6.2) |  |  |  |
| Animal scratch | 22 | 6.63(4.34-10.13) | Hepatosplenic candidiasis | 11 | 76.01(40.66-142.1) |  |  |  |
| Cholecystitis infective | 22 | 2.5(1.64-3.8) | Forced expiratory volume decreased | 11 | 2.84(1.57-5.14) |  |  |  |
| Subcutaneous haematoma | 22 | 5.21(3.41-7.95) | Aspergillus test positive | 11 | 33.58(18.31-61.58) |  |  |  |
| Post procedural swelling | 22 | 5.54(3.63-8.46) | Cytomegalovirus infection reactivation | 11 | 2.03(1.12-3.67) |  |  |  |
| Oesophageal haemorrhage | 22 | 5.5(3.6-8.39) | Transposition of the great vessels | 11 | 16.35(8.99-29.75) |  |  |  |
| Infusion | 21 | 13.62(8.76-21.17) | Neuropsychological symptoms | 11 | 5.14(2.84-9.31) |  |  |  |
| Pustule | 21 | 2.38(1.55-3.66) | Secondary adrenocortical insufficiency | 11 | 5.79(3.2-10.48) |  |  |  |
| Lipoma | 21 | 2.59(1.68-3.98) | Acute lymphocytic leukaemia | 11 | 3.37(1.86-6.09) |  |  |  |
| Neoplasm skin | 21 | 3.92(2.55-6.03) | Acute febrile neutrophilic dermatosis | 11 | 4.52(2.5-8.18) |  |  |  |
| Palliative care | 21 | 3.58(2.33-5.51) | Sputum increased | 10 | 3.72(2-6.92) |  |  |  |
| Toe operation | 21 | 8.52(5.51-13.18) | Kounis syndrome | 10 | 3.54(1.9-6.6) |  |  |  |
| Protein total abnormal | 21 | 4.19(2.72-6.45) | Dysbiosis | 10 | 7.44(3.99-13.88) |  |  |  |
| Pneumonia cryptococcal | 21 | 6.74(4.37-10.41) | Hepatitis toxic | 10 | 4.12(2.21-7.66) |  |  |  |
| Oral blood blister | 21 | 29.93(18.97-47.24) | Solar lentigo | 10 | 7.38(3.96-13.77) |  |  |  |
| Escherichia sepsis | 21 | 2.4(1.56-3.69) | Pruritus genital | 10 | 3.48(1.87-6.47) |  |  |  |
| Tympanic membrane perforation | 21 | 3.66(2.38-5.63) | Bradyarrhythmia | 10 | 6.58(3.53-12.26) |  |  |  |
| Acute febrile neutrophilic dermatosis | 21 | 2.99(1.94-4.6) | Hepatic pain | 10 | 2.05(1.1-3.81) |  |  |  |
| Nephrectomy | 20 | 4.31(2.77-6.71) | Deafness bilateral | 10 | 5.38(2.89-10.02) |  |  |  |
| Onychalgia | 20 | 5.72(3.67-8.91) | Ischaemic hepatitis | 10 | 7.79(4.18-14.54) |  |  |  |
| Infected bite | 20 | 5.16(3.31-8.05) | Cortisol decreased | 10 | 5.33(2.86-9.93) |  |  |  |
| Bladder neoplasm | 20 | 3.6(2.31-5.6) | Congenital aplasia | 10 | 3.9(2.09-7.26) |  |  |  |
| Left ventricular failure | 20 | 2.03(1.31-3.16) | Bone marrow disorder | 10 | 3.1(1.66-5.77) |  |  |  |
| Metabolic encephalopathy | 20 | 2.15(1.38-3.34) | Adenovirus infection | 10 | 2.9(1.56-5.4) |  |  |  |
| Blood iron abnormal | 20 | 5.22(3.35-8.13) | Neoplasm recurrence | 10 | 2.69(1.45-5.01) |  |  |  |
| Endodontic procedure | 20 | 2.5(1.61-3.88) | Visual brightness | 10 | 28.58(15.16-53.87) |  |  |  |
| Atrioventricular block second degree | 20 | 2.34(1.5-3.63) | Opportunistic infection | 10 | 5.1(2.74-9.51) |  |  |  |
| Sars-cov-2 antibody test negative | 20 | 9.57(6.11-14.97) | Burns second degree | 10 | 5.43(2.91-10.12) |  |  |  |
| Central venous catheter removal | 20 | 11.87(7.57-18.62) | Stem cell transplant | 10 | 5.29(2.84-9.86) |  |  |  |
| Discharge | 19 | 3.46(2.2-5.45) | Vanishing bile duct syndrome | 10 | 8.06(4.32-15.04) |  |  |  |
| Nail operation | 19 | 13.54(8.52-21.53) | Electrocardiogram t wave inversion | 10 | 5.71(3.06-10.64) |  |  |  |
| Splenectomy | 19 | 5.73(3.64-9.04) | Cardioactive drug level increased | 10 | 11.84(6.33-22.14) |  |  |  |
| Nail ridging | 19 | 9.38(5.92-14.84) | Central nervous system fungal infection | 10 | 35.33(18.68-66.79) |  |  |  |
| Endotracheal intubation | 19 | 2.09(1.33-3.28) | Myoglobin blood increased | 10 | 8.11(4.35-15.14) |  |  |  |
| Noninfective gingivitis | 19 | 2.99(1.9-4.71) | Acute graft versus host disease | 10 | 2.13(1.15-3.97) |  |  |  |
| Bone marrow disorder | 19 | 2.04(1.3-3.2) | Apparent mineralocorticoid excess | 10 | 190.54(93.91-386.62) |  |  |  |
| Aortic valve stenosis | 19 | 3.53(2.24-5.56) | Respiratory tract infection fungal | 10 | 16.55(8.83-31.01) |  |  |  |
| Hyperleukocytosis | 19 | 5.33(3.38-8.39) | Lip pain | 9 | 2.85(1.48-5.48) |  |  |  |
| Infection susceptibility increased | 19 | 2.32(1.48-3.64) | Palatal oedema | 9 | 6.7(3.47-12.92) |  |  |  |
| B-cell small lymphocytic lymphoma | 19 | 32.16(19.86-52.07) | Nocardiosis | 9 | 2.86(1.48-5.5) |  |  |  |
| Calculus bladder | 18 | 3.99(2.5-6.36) | Vascular purpura | 9 | 6.44(3.34-12.42) |  |  |  |
| Marginal zone lymphoma | 18 | 23.08(14.19-37.52) | Skin erosion | 9 | 2.29(1.19-4.41) |  |  |  |
| Aplasia pure red cell | 18 | 2.04(1.28-3.24) | Lip exfoliation | 9 | 5.91(3.06-11.39) |  |  |  |
| Protein total increased | 18 | 2.12(1.33-3.38) | Optic neuropathy | 9 | 4.04(2.1-7.79) |  |  |  |
| Prostate infection | 18 | 5.6(3.51-8.95) | Hepatic cyst | 9 | 2.57(1.33-4.94) |  |  |  |
| Myocardial injury | 18 | 2.7(1.7-4.3) | Exophthalmos | 9 | 4.01(2.08-7.73) |  |  |  |
| Hyperphosphataemia | 18 | 2.73(1.72-4.35) | Dysaesthesia | 9 | 2.94(1.53-5.66) |  |  |  |
| Peripheral artery occlusion | 18 | 2.85(1.79-4.54) | Retinal toxicity | 9 | 6.44(3.34-12.42) |  |  |  |
| Facial operation | 17 | 35.41(21.21-59.14) | Superinfection bacterial | 9 | 9(4.66-17.37) |  |  |  |
| Pulse abnormal | 17 | 2.09(1.29-3.36) | Cerebellar syndrome | 9 | 4.44(2.3-8.55) |  |  |  |
| Radiotherapy | 17 | 2.51(1.56-4.05) | Cutaneous mucormycosis | 9 | 42.55(21.66-83.58) |  |  |  |
| Ear neoplasm | 17 | 18.05(11.01-29.61) | Endotracheal intubation | 9 | 2.86(1.49-5.5) |  |  |  |
| Cryptococcosis | 17 | 3.11(1.93-5.02) | Sigmoid sinus thrombosis | 9 | 141.48(68.65-291.57) |  |  |  |
| Cystocele | 17 | 2.91(1.81-4.7) | Enterococcal bacteraemia | 9 | 7.81(4.04-15.06) |  |  |  |
| Haematocrit abnormal | 17 | 3.81(2.36-6.15) | Ventricular hypokinesia | 9 | 2.88(1.5-5.55) |  |  |  |
| Pharyngeal haemorrhage | 17 | 4.28(2.65-6.91) | Drug level abnormal | 9 | 12.75(6.59-24.66) |  |  |  |
| Rotator cuff repair | 17 | 3.09(1.91-4.99) | Bk virus infection | 9 | 2.11(1.1-4.06) |  |  |  |
| Splenic infarction | 17 | 3.19(1.98-5.15) | Ulcerative keratitis | 9 | 3.07(1.59-5.9) |  |  |  |
| Catheter placement | 17 | 2.64(1.64-4.26) | Prothrombin time prolonged | 9 | 2.28(1.19-4.4) |  |  |  |
| Aortic valve disease | 17 | 3.6(2.23-5.81) | Acinetobacter test positive | 9 | 48.79(24.76-96.13) |  |  |  |
| Chronic lymphocytic leukaemia transformation | 17 | 44.92(26.64-75.73) | Infection in an immunocompromised host | 9 | 20.36(10.48-39.54) |  |  |  |
| Lymphocyte adoptive therapy | 17 | 14.67(8.98-23.98) | Chronic graft versus host disease | 9 | 2.29(1.19-4.41) |  |  |  |
| Gastrointestinal tube insertion | 17 | 2.21(1.37-3.56) | Gastrointestinal tube insertion | 9 | 3.37(1.75-6.49) |  |  |  |
| Blood viscosity increased | 17 | 10.43(6.41-16.98) | Incorrect product dosage form administered | 9 | 8.75(4.53-16.89) |  |  |  |
| Aspiration pleural cavity | 17 | 6.33(3.91-10.25) | Creatinine renal clearance decreased | 9 | 2.18(1.13-4.2) |  |  |  |
| Laziness | 16 | 2.56(1.56-4.19) | Bundle branch block right | 9 | 2.21(1.15-4.25) |  |  |  |
| Stomach mass | 16 | 5.6(3.41-9.2) | Metastatic squamous cell carcinoma | 9 | 15.46(7.98-29.96) |  |  |  |
| Pulmonary sepsis | 16 | 2.99(1.83-4.9) | Hypothalamic pituitary adrenal axis suppression | 9 | 19.58(10.09-38.02) |  |  |  |
| Haematospermia | 16 | 7.82(4.75-12.89) | Infectious pleural effusion | 9 | 4.61(2.39-8.89) |  |  |  |
| Testicular swelling | 16 | 4.17(2.54-6.84) | Stenotrophomonas infection | 9 | 6.01(3.12-11.58) |  |  |  |
| Mechanical ventilation | 16 | 3.03(1.85-4.96) | Myelitis | 8 | 4.61(2.3-9.23) |  |  |  |
| Spinal column injury | 16 | 2(1.22-3.28) | Blastomycosis | 8 | 13.24(6.57-26.66) |  |  |  |
| Oesophageal spasm | 16 | 3.15(1.93-5.17) | Hyperaemia | 8 | 3.67(1.83-7.36) |  |  |  |
| Blood immunoglobulin a decreased | 16 | 7.16(4.35-11.79) | Costochondritis | 8 | 4.76(2.38-9.55) |  |  |  |
| Central nervous system infection | 16 | 4.05(2.47-6.64) | Acute leukaemia | 8 | 5.69(2.84-11.41) |  |  |  |
| Body temperature abnormal | 16 | 2.12(1.3-3.47) | Choreoathetosis | 8 | 12.39(6.15-24.94) |  |  |  |
| Stenosis | 15 | 2(1.2-3.33) | Abscess fungal | 8 | 46.15(22.51-94.62) |  |  |  |
| Tumour flare | 15 | 7.52(4.5-12.59) | Candida sepsis | 8 | 9.33(4.64-18.76) |  |  |  |
| Lymphadenectomy | 15 | 4.83(2.89-8.05) | Sjs-ten overlap | 8 | 10.1(5.02-20.31) |  |  |  |
| Ankle operation | 15 | 2.98(1.79-4.96) | Lip haemorrhage | 8 | 5.76(2.87-11.55) |  |  |  |
| Wrist surgery | 15 | 4.62(2.77-7.71) | Exfoliative rash | 8 | 2.89(1.44-5.8) |  |  |  |
| Platelet transfusion | 15 | 3.01(1.81-5.01) | Orthopnoea | 8 | 2.5(1.25-5) |  |  |  |
| Urine flow decreased | 15 | 2.06(1.24-3.42) | Mucosal disorder | 8 | 3.6(1.8-7.21) |  |  |  |
| Vaccination complication | 15 | 4.61(2.76-7.69) | Haemothorax | 8 | 2.47(1.23-4.94) |  |  |  |
| Immunoglobulins abnormal | 15 | 16.84(9.96-28.48) | Mediastinitis | 8 | 12.21(6.06-24.58) |  |  |  |
| Painful respiration | 15 | 2.02(1.21-3.35) | Ectropion | 8 | 21.14(10.45-42.76) |  |  |  |
| Chondrocalcinosis | 15 | 3.24(1.95-5.39) | Lung abscess | 8 | 3.53(1.76-7.07) |  |  |  |
| Meningitis cryptococcal | 15 | 2.71(1.63-4.51) | Bicytopenia | 8 | 3.35(1.67-6.7) |  |  |  |
| Gastric operation | 15 | 5.22(3.13-8.72) | Fungal oesophagitis | 8 | 10.46(5.2-21.03) |  |  |  |
| Intra-abdominal haemorrhage | 15 | 2.09(1.26-3.48) | Meningitis fungal | 8 | 38.4(18.81-78.4) |  |  |  |
| Red blood cell abnormality | 15 | 4.9(2.94-8.18) | Linear iga disease | 8 | 3.93(1.96-7.88) |  |  |  |
| Retroperitoneal haemorrhage | 15 | 2.25(1.35-3.74) | Procalcitonin increased | 8 | 5.33(2.66-10.69) |  |  |  |
| Staphylococcal skin infection | 15 | 3.92(2.36-6.54) | Neutrophilic dermatosis | 8 | 14.93(7.4-30.09) |  |  |  |
| Retinal oedema | 14 | 2.36(1.39-4) | Eye infection fungal | 8 | 20.53(10.15-41.52) |  |  |  |
| Thyroidectomy | 14 | 2.44(1.44-4.13) | Language disorder | 8 | 2.53(1.26-5.06) |  |  |  |
| Axillary pain | 14 | 2.76(1.63-4.68) | Acute graft versus host disease in intestine | 8 | 6.19(3.09-12.43) |  |  |  |
| Colon neoplasm | 14 | 4.65(2.74-7.9) | Blood creatinine decreased | 8 | 2.22(1.11-4.44) |  |  |  |
| Lumbar puncture | 14 | 11.45(6.69-19.59) | Peripheral vein thrombosis | 8 | 3.25(1.62-6.5) |  |  |  |
| Skin graft | 14 | 7.09(4.16-12.07) | Anticoagulation drug level above therapeutic | 8 | 3.92(1.96-7.86) |  |  |  |
| Haemoperitoneum | 14 | 2.02(1.19-3.42) | Ocular surface squamous neoplasia | 8 | 264.75(115.89-604.79) |  |  |  |
| Intracranial mass | 14 | 3.17(1.87-5.38) | Thrombotic thrombocytopenic purpura | 8 | 2.51(1.26-5.03) |  |  |  |
| Abdominal neoplasm | 14 | 4.96(2.92-8.43) | Mycobacterium abscessus infection | 8 | 9.01(4.49-18.12) |  |  |  |
| Pharyngeal operation | 14 | 13.13(7.66-22.52) | Pseudomonas test positive | 8 | 8.29(4.13-16.65) |  |  |  |
| Infected skin ulcer | 14 | 2.28(1.35-3.86) | Gastrointestinal mucormycosis | 8 | 37.54(18.39-76.61) |  |  |  |
| Blood electrolytes abnormal | 14 | 3.99(2.35-6.77) | Mycobacterium avium complex infection | 8 | 2.71(1.35-5.43) |  |  |  |
| Thyroid hormones decreased | 14 | 4.33(2.55-7.35) | Familial periodic paralysis | 8 | 32.45(15.94-66.05) |  |  |  |
| Colon operation | 13 | 3.53(2.04-6.11) | Cd4 lymphocytes decreased | 8 | 3.87(1.93-7.75) |  |  |  |
| Axillary mass | 13 | 3.88(2.24-6.71) | Vitritis | 7 | 3.21(1.53-6.75) |  |  |  |
| Sternal fracture | 13 | 3.23(1.87-5.59) | Hypogonadism | 7 | 5.71(2.71-12.02) |  |  |  |
| Pancreatic cyst | 13 | 2.14(1.24-3.7) | Renal ischaemia | 7 | 11.96(5.66-25.27) |  |  |  |
| Biopsy lung | 13 | 10.39(5.96-18.13) | Colour blindness | 7 | 8.02(3.8-16.9) |  |  |  |
| Leukostasis syndrome | 13 | 26.82(15.07-47.73) | Ocular toxicity | 7 | 6.05(2.87-12.73) |  |  |  |
| Fungal foot infection | 13 | 8.51(4.89-14.81) | Areflexia | 7 | 3.02(1.44-6.34) |  |  |  |
| Capillary fragility | 13 | 7.53(4.33-13.09) | Steatohepatitis | 7 | 8.93(4.23-18.83) |  |  |  |
| Dacryostenosis acquired | 13 | 2.81(1.63-4.86) | Metamorphopsia | 7 | 3.44(1.63-7.22) |  |  |  |
| Allergy to arthropod bite | 13 | 12.3(7.03-21.51) | Erythropsia | 7 | 80.03(36.44-175.72) |  |  |  |
| Ventricular tachyarrhythmia | 13 | 11.88(6.8-20.77) | Encephalitis fungal | 7 | 81.51(37.09-179.13) |  |  |  |
| Cardiac death | 12 | 3.78(2.14-6.7) | Escherichia bacteraemia | 7 | 2.62(1.25-5.5) |  |  |  |
| Otorrhoea | 12 | 2.06(1.16-3.63) | Bronchopleural fistula | 7 | 17.06(8.05-36.15) |  |  |  |
| Otitis externa | 12 | 3.25(1.84-5.75) | Product monitoring error | 7 | 2.78(1.32-5.83) |  |  |  |
| Bladder mass | 12 | 7.6(4.27-13.52) | Enterobacter infection | 7 | 4.09(1.94-8.59) |  |  |  |
| Hydrocele | 12 | 3.63(2.05-6.42) | Tooth discolouration | 7 | 2.59(1.23-5.44) |  |  |  |
| Onycholysis | 12 | 2.25(1.27-3.96) | Autonomic neuropathy | 7 | 6.49(3.08-13.67) |  |  |  |
| Lip injury | 12 | 2.48(1.4-4.38) | Transplant failure | 7 | 2.64(1.26-5.54) |  |  |  |
| Bladder catheterisation | 12 | 3.86(2.18-6.83) | Fallot's tetralogy | 7 | 8.26(3.92-17.41) |  |  |  |
| Thyroid operation | 12 | 6.07(3.42-10.78) | Infection reactivation | 7 | 4.48(2.13-9.43) |  |  |  |
| Dermatitis infected | 12 | 7.08(3.99-12.59) | Penicillium infection | 7 | 29.94(14.03-63.91) |  |  |  |
| Shoulder fracture | 12 | 2.37(1.34-4.18) | Mucosal haemorrhage | 7 | 5.59(2.66-11.77) |  |  |  |
| Pneumonia streptococcal | 12 | 3.68(2.08-6.52) | Hypoaldosteronism | 7 | 24.18(11.37-51.45) |  |  |  |
| Gastric ph decreased | 12 | 3.5(1.98-6.2) | Burns first degree | 7 | 26.84(12.6-57.19) |  |  |  |
| Spontaneous haemorrhage | 12 | 3.06(1.73-5.41) | Alternaria infection | 7 | 18.73(8.83-39.72) |  |  |  |
| Blood uric acid abnormal | 12 | 9.19(5.16-16.37) | Pulmonary infarction | 7 | 3.63(1.73-7.63) |  |  |  |
| Coagulation time prolonged | 12 | 2.9(1.64-5.12) | Vulvovaginal swelling | 7 | 2.86(1.36-6.01) |  |  |  |
| Blood electrolytes decreased | 12 | 2.55(1.44-4.5) | Laryngitis fungal | 7 | 44.91(20.86-96.69) |  |  |  |
| Mantle cell lymphoma refractory | 12 | 61.9(32.59-117.58) | Gastric perforation | 7 | 2.29(1.09-4.81) |  |  |  |
| Precancerous cells present | 12 | 2.49(1.41-4.41) | Drug level fluctuating | 7 | 8.73(4.14-18.41) |  |  |  |
| Neuroendocrine carcinoma of the skin | 12 | 3.48(1.96-6.15) | Aspartate aminotransferase abnormal | 7 | 4.29(2.04-9.01) |  |  |  |
| Cerebral fungal infection | 12 | 14.13(7.88-25.34) | Heart transplant rejection | 7 | 6.46(3.07-13.61) |  |  |  |
| Retinal vascular occlusion | 12 | 4.52(2.55-8.01) | Prothrombin time shortened | 7 | 6.16(2.93-12.98) |  |  |  |
| Renal surgery | 11 | 5.04(2.77-9.16) | Drug monitoring procedure not performed | 7 | 2.78(1.32-5.83) |  |  |  |
| Heat stroke | 11 | 2.27(1.25-4.1) | Intraventricular haemorrhage | 7 | 2.45(1.17-5.15) |  |  |  |
| Lip ulceration | 11 | 3.09(1.7-5.6) | Electrocardiogram t wave abnormal | 7 | 5.62(2.67-11.83) |  |  |  |
| Intestinal mass | 11 | 2.37(1.31-4.29) | Atrioventricular block second degree | 7 | 2.36(1.12-4.95) |  |  |  |
| Ocular neoplasm | 11 | 7.24(3.97-13.21) | Colour blindness acquired | 7 | 25.44(11.95-54.17) |  |  |  |
| Uterine polyp | 11 | 2.24(1.23-4.05) | Idiosyncratic drug reaction | 7 | 7.56(3.59-15.93) |  |  |  |
| Breast operation | 11 | 4.87(2.68-8.86) | Blood phosphorus increased | 7 | 2.38(1.13-4.99) |  |  |  |
| Blister rupture | 11 | 3.71(2.04-6.73) | Glucocorticoid deficiency | 7 | 12.33(5.83-26.05) |  |  |  |
| Cardioversion | 11 | 2.41(1.33-4.36) | Central nervous system infection | 7 | 5.09(2.42-10.71) |  |  |  |
| Venous occlusion | 11 | 2.01(1.11-3.63) | Maternal drugs affecting foetus | 7 | 2.14(1.02-4.49) |  |  |  |
| Prostatic mass | 11 | 29.42(15.67-55.23) | Antipsychotic drug level increased | 7 | 2.35(1.12-4.93) |  |  |  |
| Bloody discharge | 11 | 3.07(1.69-5.57) | Miliaria | 6 | 2.61(1.17-5.83) |  |  |  |
| Immunisation reaction | 11 | 3.86(2.13-7.01) | Viraemia | 6 | 3.32(1.49-7.41) |  |  |  |
| Inner ear disorder | 11 | 3.57(1.97-6.47) | Neuromyopathy | 6 | 5.45(2.44-12.18) |  |  |  |
| Wisdom teeth removal | 11 | 2.41(1.33-4.37) | Pleuritic pain | 6 | 2.52(1.13-5.63) |  |  |  |
| Precancerous condition | 11 | 4.39(2.42-7.97) | Abdominal sepsis | 6 | 6.24(2.79-13.94) |  |  |  |
| Splenic haematoma | 11 | 11.98(6.53-21.98) | Lip ulceration | 6 | 4.86(2.18-10.86) |  |  |  |
| Pneumococcal sepsis | 11 | 5.28(2.91-9.61) | Periarthritis | 6 | 2.48(1.11-5.53) |  |  |  |
| Allergy to vaccine | 11 | 9.2(5.03-16.82) | Oedema genital | 6 | 10.97(4.89-24.58) |  |  |  |
| Penile haemorrhage | 11 | 4.03(2.22-7.31) | Faeces pale | 6 | 2.51(1.13-5.6) |  |  |  |
| Deafness bilateral | 11 | 2.04(1.13-3.69) | Hyperkinesia | 6 | 2.46(1.1-5.48) |  |  |  |
| Bladder obstruction | 11 | 7.84(4.3-14.31) | Crystalluria | 6 | 8.55(3.82-19.15) |  |  |  |
| Postoperative thrombosis | 11 | 5.62(3.09-10.23) | Nodal rhythm | 6 | 5.09(2.28-11.37) |  |  |  |
| Traumatic intracranial haemorrhage | 11 | 3.13(1.72-5.67) | Hypoperfusion | 6 | 6.39(2.86-14.29) |  |  |  |
| Gastrointestinal ulcer haemorrhage | 11 | 3.33(1.83-6.04) | Genital pain | 6 | 4.52(2.02-10.09) |  |  |  |
| Breast conserving surgery | 11 | 4.26(2.34-7.73) | Corneal disorder | 6 | 2.64(1.18-5.88) |  |  |  |
| Disseminated cryptococcosis | 11 | 4.26(2.35-7.74) | Sleep talking | 6 | 3.37(1.51-7.51) |  |  |  |
| Suture insertion | 10 | 5.54(2.96-10.38) | Macular opacity | 6 | 377.26(137.11-1038.04) |  |  |  |
| Elbow operation | 10 | 4.46(2.38-8.34) | Precocious puberty | 6 | 9.7(4.33-21.72) |  |  |  |
| Surgical failure | 10 | 2.31(1.24-4.3) | Cerebral artery embolism | 6 | 5.06(2.26-11.29) |  |  |  |
| Fungaemia | 10 | 2.01(1.08-3.74) | Oncologic complication | 6 | 4.82(2.16-10.76) |  |  |  |
| Sinusitis fungal | 10 | 3.75(2.01-7.02) | Fungal disease carrier | 6 | 44.38(19.39-101.58) |  |  |  |
| Acute sinusitis | 10 | 2.1(1.13-3.91) | Klebsiella sepsis | 6 | 5.01(2.24-11.19) |  |  |  |
| Streptococcal sepsis | 10 | 2.88(1.54-5.37) | Spinal cord disorder | 6 | 3.45(1.55-7.71) |  |  |  |
| Biopsy bone marrow | 10 | 5.86(3.12-10.97) | Soft tissue swelling | 6 | 7.38(3.3-16.51) |  |  |  |
| Pyogenic granuloma | 10 | 7.03(3.75-13.2) | Thrombophlebitis septic | 6 | 30.67(13.52-69.6) |  |  |  |
| Blood bilirubin abnormal | 10 | 2.5(1.34-4.67) | Myelitis transverse | 6 | 4.15(1.86-9.26) |  |  |  |
| Cystitis noninfective | 10 | 2.25(1.2-4.19) | Urinary bladder rupture | 6 | 23.88(10.57-53.96) |  |  |  |
| Carotid artery disease | 10 | 3.47(1.86-6.48) | Intracranial haematoma | 6 | 9.2(4.11-20.6) |  |  |  |
| Ear tube insertion | 10 | 10.37(5.5-19.55) | Meningitis coccidioides | 6 | 198.56(79.3-497.18) |  |  |  |
| Conduction disorder | 10 | 2.14(1.15-3.99) | Lichenoid keratosis | 6 | 2.44(1.09-5.44) |  |  |  |
| Oesophageal operation | 10 | 14.16(7.47-26.85) | Pneumonia cryptococcal | 6 | 5.47(2.45-12.21) |  |  |  |
| Pulmonary resection | 10 | 4.7(2.51-8.79) | Alcohol intolerance | 6 | 7.78(3.48-17.4) |  |  |  |
| Prostatic haemorrhage | 10 | 9.42(5-17.74) | Aortoenteric fistula | 6 | 61.85(26.74-143.06) |  |  |  |
| Abdominal operation | 10 | 2.39(1.28-4.46) | Acute lung injury | 6 | 3.74(1.67-8.33) |  |  |  |
| Wound infection staphylococcal | 10 | 2.3(1.23-4.29) | Rectal perforation | 6 | 10.84(4.84-24.3) |  |  |  |
| West nile viral infection | 10 | 4.29(2.29-8.02) | Vascular pseudoaneurysm | 6 | 3.24(1.45-7.22) |  |  |  |
| Cold type haemolytic anaemia | 10 | 14.07(7.42-26.67) | Candida pneumonia | 6 | 20.5(9.09-46.23) |  |  |  |
| Thyroid hormones increased | 10 | 2.28(1.22-4.25) | Coagulation time prolonged | 6 | 4.18(1.87-9.32) |  |  |  |
| Cardiac pacemaker replacement | 10 | 5.76(3.07-10.8) | Clostridium test positive | 6 | 2.25(1.01-5.02) |  |  |  |
| Intra-abdominal haematoma | 10 | 2.51(1.35-4.69) | Chronic respiratory failure | 6 | 4.52(2.03-10.1) |  |  |  |
| Cellulitis staphylococcal | 10 | 5.23(2.79-9.8) | Disseminated blastomycosis | 6 | 27.74(12.25-62.84) |  |  |  |
| Sarcoma | 9 | 2.57(1.33-4.96) | Electrocardiogram qt shortened | 6 | 18.31(8.13-41.24) |  |  |  |
| Bite | 9 | 3.8(1.97-7.35) | Angina bullosa haemorrhagica | 6 | 17.07(7.59-38.41) |  |  |  |
| Cochlea implant | 9 | 21.91(11.04-43.49) | Congenital central nervous system anomaly | 6 | 5.6(2.51-12.5) |  |  |  |
| Pharyngeal mass | 9 | 4.95(2.56-9.58) | Complications of transplant surgery | 6 | 19.15(8.5-43.15) |  |  |  |
| Eyelid operation | 9 | 10.21(5.23-19.92) | Cytomegalovirus chorioretinitis | 6 | 2.67(1.2-5.94) |  |  |  |
| Biopsy prostate | 9 | 31.45(15.63-63.28) | Concomitant disease progression | 6 | 3.96(1.77-8.83) |  |  |  |
| Listeriosis | 9 | 2.47(1.28-4.77) | Lower respiratory tract infection fungal | 6 | 13.43(5.98-30.14) |  |  |  |
| Oral contusion | 9 | 9.8(5.02-19.11) | Demyelinating polyneuropathy | 6 | 5.89(2.63-13.15) |  |  |  |
| Joint warmth | 9 | 2.2(1.14-4.25) | Bacterial disease carrier | 6 | 6.9(3.08-15.42) |  |  |  |
| Fungal sepsis | 9 | 4(2.07-7.73) | Electrocardiogram j wave abnormal | 6 | 290.2(110.3-763.52) |  |  |  |
| Spontaneous haematoma | 9 | 3.48(1.8-6.71) | Congenital musculoskeletal disorder of skull | 6 | 10.63(4.74-23.82) |  |  |  |
| Obstruction gastric | 9 | 2.17(1.12-4.18) | Hyperglycaemic hyperosmolar nonketotic syndrome | 6 | 4.18(1.87-9.32) |  |  |  |
| Head and neck cancer | 9 | 4(2.07-7.73) | Central nervous system vasculitis | 6 | 6.14(2.75-13.73) |  |  |  |
| Pleuropericarditis | 9 | 7.56(3.89-14.69) | Subacute cutaneous lupus erythematosus | 6 | 2.35(1.05-5.24) |  |  |  |
| Scleral haemorrhage | 9 | 15.23(7.75-29.95) | Eschar | 5 | 6.79(2.81-16.39) |  |  |  |
| Hyperviscosity syndrome | 9 | 11.96(6.11-23.4) | Xerosis | 5 | 3.65(1.52-8.79) |  |  |  |
| Platelet dysfunction | 9 | 8.19(4.21-15.94) | Myopericarditis | 5 | 7.26(3.01-17.53) |  |  |  |
| Bing-neel syndrome | 9 | 84.77(39.23-183.21) | Gingival blister | 5 | 10.95(4.52-26.52) |  |  |  |
| Transient global amnesia | 9 | 3.74(1.94-7.23) | Myopathy toxic | 5 | 8.12(3.36-19.63) |  |  |  |
| Vestibular disorder | 9 | 2.53(1.31-4.89) | Hyperlipasaemia | 5 | 7.86(3.25-18.99) |  |  |  |
| Oncologic complication | 9 | 2.5(1.3-4.82) | Elbow synostosis | 5 | 785.94(211.04-2926.94) |  |  |  |
| Spinal cord neoplasm | 9 | 2.91(1.51-5.62) | Quadriparesis | 5 | 2.7(1.12-6.5) |  |  |  |
| Pneumonia influenzal | 9 | 2.08(1.08-4.01) | Food interaction | 5 | 2.77(1.15-6.67) |  |  |  |
| Blood sodium abnormal | 9 | 2.66(1.38-5.13) | Tongue coated | 5 | 2.77(1.15-6.67) |  |  |  |
| Lymphocytic leukaemia | 9 | 7.17(3.69-13.93) | Hypoproteinaemia | 5 | 2.92(1.21-7.02) |  |  |  |
| Breast haemorrhage | 9 | 7.93(4.08-15.42) | Electric shock | 5 | 5.45(2.26-13.14) |  |  |  |
| Thrombotic stroke | 9 | 4.51(2.33-8.73) | Blister rupture | 5 | 4.84(2.01-11.68) |  |  |  |
| Spinal cord haemorrhage | 9 | 6.5(3.35-12.61) | Tonic convulsion | 5 | 3.53(1.46-8.5) |  |  |  |
| Intervertebral disc compression | 9 | 2.72(1.41-5.24) | Craniosynostosis | 5 | 5.92(2.45-14.28) |  |  |  |
| Prostate cancer recurrent | 9 | 4.31(2.23-8.34) | Hyporeflexia | 5 | 2.99(1.24-7.21) |  |  |  |
| Oesophageal dilation procedure | 9 | 16.39(8.32-32.26) | Hydrothorax | 5 | 4.85(2.01-11.7) |  |  |  |
| Gastrointestinal polyp haemorrhage | 9 | 3.6(1.87-6.96) | Lipohypertrophy | 5 | 7.18(2.97-17.33) |  |  |  |
| Left atrial appendage closure implant | 9 | 33.05(16.39-66.64) | Brachycephaly | 5 | 69.86(27.73-176) |  |  |  |
| Nail injury | 8 | 4.02(2-8.09) | Hypospadias | 5 | 2.46(1.02-5.91) |  |  |  |
| Endoscopy | 8 | 4.17(2.07-8.39) | Mucosal erosion | 5 | 6.08(2.52-14.67) |  |  |  |
| Blister infected | 8 | 3(1.49-6.04) | Haematoma muscle | 5 | 3.03(1.26-7.28) |  |  |  |
| Solar lentigo | 8 | 2.03(1.01-4.07) | Angiodermatitis | 5 | 55.15(22.11-137.6) |  |  |  |
| Vertebroplasty | 8 | 7.31(3.61-14.79) | Leukoplakia oral | 5 | 9.36(3.87-22.63) |  |  |  |
| Purpura senile | 8 | 22.51(10.87-46.62) | Tinea pedis | 5 | 2.95(1.23-7.11) |  |  |  |
| Allergic cough | 8 | 2.67(1.33-5.36) | Photoonycholysis | 5 | 108.41(41.96-280.06) |  |  |  |
| Ingrown hair | 8 | 3.31(1.65-6.65) | Renal abscess | 5 | 5.94(2.46-14.34) |  |  |  |
| Serositis | 8 | 4.91(2.44-9.89) | Spontaneous haematoma | 5 | 5.56(2.31-13.42) |  |  |  |
| Keratomileusis | 8 | 9.79(4.82-19.89) | Slow response to stimuli | 5 | 3.26(1.35-7.85) |  |  |  |
| Pseudohyperkalaemia | 8 | 82.53(36.56-186.33) | Hepatobiliary disease | 5 | 6.69(2.77-16.15) |  |  |  |
| Corneal transplant | 8 | 3.45(1.72-6.94) | Osteomyelitis fungal | 5 | 29.38(11.98-72.04) |  |  |  |
| Allergic sinusitis | 8 | 2.52(1.25-5.05) | Infective keratitis | 5 | 54.2(21.74-135.14) |  |  |  |
| Defaecation disorder | 8 | 2.93(1.46-5.88) | Blood ph decreased | 5 | 5.27(2.18-12.7) |  |  |  |
| Skin neoplasm excision | 8 | 3.01(1.5-6.05) | Hypoprothrombinaemia | 5 | 17.96(7.38-43.7) |  |  |  |
| Transfusion reaction | 8 | 3.34(1.66-6.71) | Lacunar infarction | 5 | 2.56(1.06-6.16) |  |  |  |
| Hepatic haemorrhage | 8 | 3.32(1.65-6.67) | Crystal arthropathy | 5 | 23.12(9.47-56.43) |  |  |  |
| Antibody test abnormal | 8 | 3.16(1.57-6.34) | Pneumomediastinum | 5 | 2.5(1.04-6.03) |  |  |  |
| Eye laser surgery | 8 | 4.67(2.32-9.41) | Vaccination complication | 5 | 4.4(1.82-10.6) |  |  |  |
| Benign neoplasm of skin | 8 | 6.85(3.39-13.85) | Pharyngeal abscess | 5 | 8.14(3.37-19.68) |  |  |  |
| Cataract operation complication | 8 | 5.84(2.89-11.78) | Axonal neuropathy | 5 | 5.35(2.22-12.89) |  |  |  |
| Medical device site infection | 8 | 3.16(1.57-6.34) | Premature menopause | 5 | 5.34(2.21-12.87) |  |  |  |
| Prostatic specific antigen decreased | 8 | 6.33(3.13-12.78) | Gallbladder oedema | 5 | 17.76(7.3-43.2) |  |  |  |
| Arteriovenous malformation | 8 | 2.92(1.45-5.86) | Gingival ulceration | 5 | 7.13(2.95-17.21) |  |  |  |
| Blood immunoglobulin g abnormal | 8 | 16.05(7.83-32.91) | Enterococcal sepsis | 5 | 6.18(2.56-14.9) |  |  |  |
| Retroperitoneal lymphadenopathy | 8 | 4.43(2.2-8.93) | Jugular vein thrombosis | 5 | 2.82(1.17-6.8) |  |  |  |
| Post procedural inflammation | 8 | 3.35(1.66-6.73) | Pulmonary necrosis | 5 | 16.46(6.77-40) |  |  |  |
| Squamous cell carcinoma of lung | 8 | 2.64(1.31-5.3) | Troponin i increased | 5 | 2.94(1.22-7.09) |  |  |  |
| Hangnail | 7 | 12.85(6-27.55) | Primary myelofibrosis | 5 | 10.51(4.34-25.45) |  |  |  |
| Cellulite | 7 | 5.32(2.51-11.26) | Somatic symptom disorder | 5 | 2.64(1.1-6.35) |  |  |  |
| Critical illness | 7 | 2.29(1.09-4.82) | Gastrointestinal polyp | 5 | 10.92(4.51-26.43) |  |  |  |
| Jaw operation | 7 | 2.99(1.42-6.29) | Citrobacter infection | 5 | 12.33(5.09-29.88) |  |  |  |
| Oligospermia | 7 | 17.23(7.98-37.21) | Dose calculation error | 5 | 6.02(2.5-14.53) |  |  |  |
| Mastoiditis | 7 | 2.31(1.1-4.87) | Salivary hyposecretion | 5 | 4.81(2-11.61) |  |  |  |
| Sinoatrial block | 7 | 4.47(2.12-9.46) | Bipolar i disorder | 5 | 2.57(1.07-6.2) |  |  |  |
| Gouty arthritis | 7 | 3.15(1.49-6.64) | Oral lichen planus | 5 | 8.83(3.65-21.35) |  |  |  |
| Renal haematoma | 7 | 4.01(1.9-8.47) | Hypertensive urgency | 5 | 7.49(3.1-18.08) |  |  |  |
| Chylothorax | 7 | 2.88(1.37-6.08) | Retroperitoneal fibrosis | 5 | 9.03(3.74-21.84) |  |  |  |
| Motor neurone disease | 7 | 5.11(2.41-10.8) | Urinary tract candidiasis | 5 | 14.97(6.17-36.34) |  |  |  |
| Antibody test negative | 7 | 8.62(4.05-18.34) | Disseminated coccidioidomycosis | 5 | 32.08(13.06-78.8) |  |  |  |
| Haemophilus infection | 7 | 2.37(1.12-4.98) | Cerebral microhaemorrhage | 5 | 10.8(4.46-26.15) |  |  |  |
| Renal cyst haemorrhage | 7 | 8.87(4.16-18.88) | Coagulation time shortened | 5 | 16.9(6.95-41.09) |  |  |  |
| Clostridial sepsis | 7 | 6.09(2.87-12.91) | Complications of transplanted lung | 5 | 13.73(5.66-33.3) |  |  |  |
| Minimal residual disease | 7 | 3.74(1.77-7.89) | Neonatal multi-organ failure | 5 | 116.44(44.84-302.36) |  |  |  |
| Oesophageal achalasia | 7 | 3.2(1.52-6.75) | Drug dispensed to wrong patient | 5 | 3.63(1.51-8.73) |  |  |  |
| Scrotal haematocoele | 7 | 31.59(14.3-69.82) | Peripheral artery thrombosis | 5 | 2.62(1.09-6.3) |  |  |  |
| Retinal operation | 7 | 8.29(3.9-17.63) | Congenital nose malformation | 5 | 13.15(5.43-31.89) |  |  |  |
| Nail bed infection | 7 | 9.54(4.47-20.33) | Acute left ventricular failure | 5 | 6.01(2.49-14.5) |  |  |  |
| Chromosomal deletion | 7 | 15.17(7.05-32.63) | Blood bilirubin unconjugated increased | 5 | 8.34(3.45-20.15) |  |  |  |
| Gingival erythema | 7 | 2.75(1.3-5.79) | Blood corticosterone increased | 5 | 785.94(211.04-2926.94) |  |  |  |
| Epidural injection | 7 | 9.48(4.45-20.2) | Eastern cooperative oncology group performance status worsened | 5 | 2.92(1.21-7.03) |  |  |  |
| Limb crushing injury | 7 | 3.08(1.46-6.5) | Enterobacter test positive | 5 | 18.38(7.56-44.74) |  |  |  |
| Oesophageal obstruction | 7 | 2.51(1.19-5.3) | Malignant melanoma stage 0 | 5 | 2.76(1.15-6.64) |  |  |  |
| Bone marrow infiltration | 7 | 4.46(2.11-9.43) | Cortical visual impairment | 5 | 87.33(34.27-222.54) |  |  |  |
| Blood urea abnormal | 7 | 2.44(1.16-5.14) | Delayed recovery from anaesthesia | 5 | 5.68(2.36-13.71) |  |  |  |
| Cardiac hypertrophy | 7 | 2.32(1.1-4.89) | Therapeutic response increased | 5 | 4.66(1.94-11.24) |  |  |  |
| Skin bacterial infection | 7 | 2.55(1.21-5.37) | Acute graft versus host disease in liver | 5 | 7.23(2.99-17.45) |  |  |  |
| Lymphoma transformation | 7 | 5.51(2.6-11.68) | Female genital tract fistula | 5 | 2.44(1.01-5.88) |  |  |  |
| Implantable cardiac monitor insertion | 7 | 18.05(8.35-39.03) | Pulmonary valve stenosis congenital | 5 | 10.69(4.42-25.88) |  |  |  |
| Spinal nerve stimulator implantation | 7 | 5.86(2.76-12.41) | Hyposmia | 4 | 3.28(1.23-8.76) |  |  |  |
| Prostatic urethral lift procedure | 7 | 137.87(53.44-355.64) | Gliosis | 4 | 10.1(3.76-27.12) |  |  |  |
| Artificial crown procedure | 7 | 4.26(2.02-9) | Lip pruritus | 4 | 3.54(1.33-9.46) |  |  |  |
| Urinary bladder suspension | 7 | 6.02(2.84-12.75) | Syndactyly | 4 | 4.53(1.69-12.12) |  |  |  |
| Arterial therapeutic procedure | 7 | 10.18(4.77-21.72) | Iris adhesions | 4 | 6.99(2.61-18.72) |  |  |  |
| Immunodeficiency common variable | 7 | 3.95(1.87-8.34) | Neonatal seizure | 4 | 6.23(2.33-16.67) |  |  |  |
| Uterine dilation and curettage | 7 | 2.66(1.26-5.6) | Renin decreased | 4 | 50.3(18.17-139.28) |  |  |  |
| Oral mucosal discolouration | 7 | 3.38(1.6-7.13) | Duodenal atresia | 4 | 33.99(12.43-92.96) |  |  |  |
| Pyoderma | 6 | 3.54(1.58-7.94) | Resting tremor | 4 | 4.7(1.76-12.57) |  |  |  |
| Rectal neoplasm | 6 | 6.95(3.08-15.67) | Gallstone ileus | 4 | 114.32(39.39-331.75) |  |  |  |
| Dental cleaning | 6 | 11.82(5.2-26.88) | Penile erythema | 4 | 8.61(3.21-23.1) |  |  |  |
| Biopsy skin | 6 | 11.21(4.93-25.46) | Polyserositis | 4 | 9.15(3.41-24.54) |  |  |  |
| Pelvic mass | 6 | 2.58(1.15-5.77) | Sputum purulent | 4 | 5.36(2-14.35) |  |  |  |
| Polyserositis | 6 | 4.76(2.12-10.69) | Protothecosis | 4 | 35.93(13.12-98.41) |  |  |  |
| Inadequate diet | 6 | 3.81(1.7-8.54) | Aspergillus test | 4 | 100.6(35.01-289.07) |  |  |  |
| Arterial repair | 6 | 10.74(4.73-24.39) | Shock symptom | 4 | 7.83(2.92-21) |  |  |  |
| Throat lesion | 6 | 2.73(1.22-6.11) | Mycetoma mycotic | 4 | 26.2(9.64-71.23) |  |  |  |
| Scrotal oedema | 6 | 2.88(1.29-6.45) | Anencephaly | 4 | 7.14(2.67-19.14) |  |  |  |
| Bone operation | 6 | 3.15(1.41-7.06) | Synostosis | 4 | 44.12(16.01-121.61) |  |  |  |
| Groin infection | 6 | 3.26(1.45-7.3) | Renal aneurysm | 4 | 15.92(5.9-42.94) |  |  |  |
| Soft tissue mass | 6 | 3.69(1.65-8.28) | Butterfly rash | 4 | 3.26(1.22-8.71) |  |  |  |
| Chorioretinitis | 6 | 2.75(1.23-6.15) | Death neonatal | 4 | 3.28(1.23-8.76) |  |  |  |
| Breast haematoma | 6 | 8.84(3.91-20) | Genital erythema | 4 | 7.06(2.64-18.93) |  |  |  |
| Polypectomy | 6 | 2.33(1.04-5.21) | Ovarian failure | 4 | 6.93(2.59-18.56) |  |  |  |
| Adrenal mass | 6 | 2.37(1.06-5.29) | Hypertelorism | 4 | 8.27(3.08-22.19) |  |  |  |
| Nasal crusting | 6 | 2.33(1.04-5.21) | Chorioretinitis | 4 | 5.29(1.98-14.17) |  |  |  |
| Autoimmune neutropenia | 6 | 6.43(2.86-14.49) | Lip erosion | 4 | 5.29(1.98-14.17) |  |  |  |
| Globulins decreased | 6 | 5(2.23-11.23) | Heart transplant | 4 | 3.89(1.45-10.39) |  |  |  |
| Nail cuticle fissure | 6 | 26(11.15-60.63) | Nasal abscess | 4 | 15.24(5.65-41.1) |  |  |  |
| Testicular haemorrhage | 6 | 22.41(9.67-51.94) | Pouchitis | 4 | 4.14(1.55-11.06) |  |  |  |
| Paraneoplastic syndrome | 6 | 3.47(1.55-7.77) | Alkalosis | 4 | 12.7(4.72-34.18) |  |  |  |
| Cellulitis orbital | 6 | 3.21(1.43-7.19) | Renal haematoma | 4 | 6.6(2.46-17.68) |  |  |  |
| Mineral supplementation | 6 | 3.39(1.51-7.58) | Nasal necrosis | 4 | 27.64(10.15-75.23) |  |  |  |
| Faecal occult blood | 6 | 3.19(1.43-7.15) | Tongue necrosis | 4 | 48.36(17.49-133.73) |  |  |  |
| Brain neoplasm benign | 6 | 3.79(1.69-8.49) | Corneal bleeding | 4 | 34.93(12.76-95.61) |  |  |  |
| Cystitis klebsiella | 6 | 12.04(5.29-27.38) | Anterior displaced anus | 4 | 86.72(30.49-246.7) |  |  |  |
| Blood viscosity abnormal | 6 | 14.77(6.46-33.77) | Vulvovaginal erythema | 4 | 3.74(1.4-10) |  |  |  |
| Gastrointestinal surgery | 6 | 2.37(1.06-5.3) | Eyelid ptosis congenital | 4 | 39.3(14.31-107.91) |  |  |  |
| Penile ulceration | 6 | 6.99(3.1-15.76) | Gallbladder enlargement | 4 | 3.45(1.29-9.22) |  |  |  |
| Nail bed disorder | 6 | 3.66(1.63-8.21) | Prostatic abscess | 4 | 14.54(5.39-39.17) |  |  |  |
| Abnormal clotting factor | 6 | 10.57(4.66-23.98) | Hepatic artery aneurysm | 4 | 43.36(15.74-119.44) |  |  |  |
| Post procedural sepsis | 6 | 2.7(1.21-6.05) | Fungal peritonitis | 4 | 3.36(1.26-8.98) |  |  |  |
| Pneumonia haemophilus | 6 | 3.75(1.67-8.4) | Blood urea decreased | 4 | 3.63(1.36-9.71) |  |  |  |
| Gastrointestinal lymphoma | 6 | 5.86(2.6-13.17) | Bleeding time shortened | 4 | 279.44(86.05-907.44) |  |  |  |
| Ocular procedural complication | 6 | 5.8(2.58-13.06) | Conversion disorder | 4 | 2.73(1.02-7.3) |  |  |  |
| Cardiac device implantation | 6 | 37.14(15.62-88.3) | Hyperadrenocorticism | 4 | 9.35(3.48-25.09) |  |  |  |
| Excessive cerumen production | 6 | 2.33(1.04-5.22) | Puerperal pyrexia | 4 | 71.86(25.54-202.18) |  |  |  |
| Post procedural haematuria | 6 | 9.49(4.19-21.49) | Necrotising retinitis | 4 | 4.9(1.83-13.11) |  |  |  |
| Campylobacter gastroenteritis | 6 | 2.7(1.21-6.03) | Subcutaneous emphysema | 4 | 3.81(1.43-10.18) |  |  |  |
| B-cell prolymphocytic leukaemia | 6 | 99.99(38-263.07) | Sinusitis aspergillus | 4 | 26.2(9.64-71.23) |  |  |  |
| Heavy exposure to ultraviolet light | 6 | 7.34(3.26-16.57) | Vulval ulceration | 4 | 13.52(5.02-36.41) |  |  |  |
| Beta 2 microglobulin increased | 6 | 9.15(4.04-20.72) | Pulmonary nocardiosis | 4 | 3.59(1.34-9.6) |  |  |  |
| Urinary tract infection staphylococcal | 6 | 5.53(2.46-12.44) | Circumoral oedema | 4 | 12.83(4.77-34.53) |  |  |  |
| Lower respiratory tract infection fungal | 6 | 4.63(2.06-10.38) | Subdiaphragmatic abscess | 4 | 41.23(14.99-113.39) |  |  |  |
| Metastatic squamous cell carcinoma | 6 | 3.52(1.57-7.89) | Vulvovaginal injury | 4 | 3.99(1.49-10.67) |  |  |  |
| Paranasal sinus haemorrhage | 6 | 14.44(6.32-33) | Pulmonary resection | 4 | 5.39(2.01-14.41) |  |  |  |
| Splinter | 5 | 6.94(2.85-16.92) | Urinary tract pain | 4 | 7.14(2.67-19.14) |  |  |  |
| Arrhythmic storm | 5 | 4.21(1.74-10.21) | Procedural nausea | 4 | 4.48(1.68-11.99) |  |  |  |
| Hearing aid user | 5 | 3.31(1.37-8.01) | Single umbilical artery | 4 | 10.02(3.73-26.91) |  |  |  |
| Stent removal | 5 | 8.33(3.41-20.36) | Necrotising scleritis | 4 | 21.5(7.93-58.23) |  |  |  |
| Grief reaction | 5 | 2.87(1.19-6.92) | Bile acids increased | 4 | 11.59(4.31-31.16) |  |  |  |
| Haemobilia | 5 | 3.76(1.55-9.11) | Cardiac valve vegetation | 4 | 9.9(3.69-26.59) |  |  |  |
| Tongue haematoma | 5 | 8.46(3.46-20.68) | Conductive deafness | 4 | 11.81(4.39-31.75) |  |  |  |
| Ear injury | 5 | 2.84(1.17-6.85) | Acanthamoeba keratitis | 4 | 93.15(32.59-266.21) |  |  |  |
| Traumatic pain | 5 | 2.7(1.12-6.53) | Hepatic infection fungal | 4 | 36.45(13.3-99.88) |  |  |  |
| Monocytosis | 5 | 2.76(1.14-6.66) | Necrotising myositis | 4 | 3.43(1.28-9.15) |  |  |  |
| Venous operation | 5 | 7.47(3.06-18.22) | Lemierre syndrome | 4 | 22.46(8.28-60.88) |  |  |  |
| Ephelides | 5 | 2.84(1.18-6.87) | Cryptococcal fungaemia | 4 | 22.86(8.43-62.01) |  |  |  |
| Nephrostomy | 5 | 4.42(1.82-10.72) | Soft tissue necrosis | 4 | 5.74(2.15-15.37) |  |  |  |
| Ligament pain | 5 | 2.54(1.05-6.13) | Osteitis deformans | 4 | 15.06(5.59-40.6) |  |  |  |
| Catheter removal | 5 | 3.97(1.64-9.61) | Viral haemorrhagic cystitis | 4 | 4.61(1.73-12.34) |  |  |  |
| Tongue eruption | 5 | 3.2(1.33-7.75) | Graft versus host disease in lung | 4 | 6.47(2.41-17.31) |  |  |  |
| Scapula fracture | 5 | 2.95(1.22-7.13) | Blood uric acid decreased | 4 | 7.76(2.9-20.81) |  |  |  |
| Nasal abscess | 5 | 6.6(2.71-16.08) | Enterococcus test positive | 4 | 5.86(2.19-15.69) |  |  |  |
| Nail pitting | 5 | 3.31(1.37-8.01) | Multiple lentigines syndrome | 4 | 89.82(31.5-256.08) |  |  |  |
| Eye infection viral | 5 | 4.46(1.84-10.81) | Heart failure with reduced ejection fraction | 4 | 10.66(3.97-28.63) |  |  |  |
| Thoracic cavity drainage | 5 | 6.12(2.52-14.89) | Urogenital infection fungal | 4 | 157.19(52.55-470.19) |  |  |  |
| Serratia infection | 5 | 2.59(1.07-6.26) | Developmental hip dysplasia | 4 | 3.57(1.34-9.53) |  |  |  |
| Dependence on respirator | 5 | 3.47(1.43-8.4) | Respiratory tract irritation | 4 | 3.42(1.28-9.14) |  |  |  |
| Haematoma infection | 5 | 2.91(1.2-7.04) | Bronchial artery embolisation | 4 | 167.67(55.64-505.2) |  |  |  |
| Type v hyperlipidaemia | 5 | 3.2(1.33-7.75) | Electrocardiogram u wave present | 4 | 44.12(16.01-121.61) |  |  |  |
| Disease risk factor | 5 | 3.43(1.42-8.29) | Blood creatine phosphokinase decreased | 4 | 10.1(3.76-27.12) |  |  |  |
| Chest tube insertion | 5 | 5.95(2.45-14.47) | Microangiopathic haemolytic anaemia | 4 | 4.63(1.73-12.39) |  |  |  |
| Blood creatine abnormal | 5 | 3.03(1.26-7.33) | Congenital absence of cranial vault | 4 | 71.86(25.54-202.18) |  |  |  |
| Pituitary haemorrhage | 5 | 6.23(2.56-15.15) | Electrocardiogram st segment abnormal | 4 | 7.04(2.63-18.87) |  |  |  |
| Oesophageal dilatation | 5 | 3.67(1.52-8.89) | Intestinal pseudo-obstruction | 4 | 2.85(1.07-7.61) |  |  |  |
| Post thrombotic syndrome | 5 | 4.2(1.73-10.17) | Neutrophil percentage increased | 4 | 3.59(1.34-9.59) |  |  |  |
| Neutrophilic dermatosis | 5 | 3.19(1.32-7.7) | Convulsive threshold lowered | 4 | 10.44(3.88-28.03) |  |  |  |
| Immunoglobulin therapy | 5 | 23.05(9.17-57.95) | Intra-abdominal haematoma | 4 | 2.9(1.08-7.74) |  |  |  |
| Cystitis escherichia | 5 | 4.46(1.84-10.81) | Hypoplastic left heart syndrome | 4 | 6.62(2.47-17.73) |  |  |  |
| Hypertensive urgency | 5 | 2.58(1.07-6.23) | Electrocardiogram qt interval abnormal | 4 | 7.49(2.79-20.06) |  |  |  |
| Skin ulcer haemorrhage | 5 | 4.01(1.66-9.72) | Congenital musculoskeletal disorder | 4 | 8.22(3.06-22.04) |  |  |  |
| Legionella infection | 5 | 2.88(1.19-6.96) | Congenital eyelid malformation | 4 | 47.45(17.17-131.11) |  |  |  |
| Fluid replacement | 5 | 3.95(1.63-9.57) | Acute monocytic leukaemia | 4 | 21.68(8-58.75) |  |  |  |
| Mammogram abnormal | 5 | 2.79(1.16-6.75) | Incorrect product formulation administered | 4 | 3.39(1.27-9.07) |  |  |  |
| Lymph gland infection | 5 | 3.09(1.28-7.46) | Blood bicarbonate decreased | 4 | 2.82(1.05-7.52) |  |  |  |
| Vertebral column mass | 5 | 4.79(1.98-11.63) | Blood beta-d-glucan increased | 4 | 17.11(6.34-46.19) |  |  |  |
| Stomatitis haemorrhagic | 5 | 13.71(5.55-33.86) | Cutaneous coccidioidomycosis | 4 | 359.28(105.17-1227.37) |  |  |  |
| Heart valve stenosis | 5 | 4.15(1.71-10.05) | Prurigo | 3 | 4.57(1.47-14.22) |  |  |  |
| Ear neoplasm malignant | 5 | 5.95(2.45-14.47) | Hypopyon | 3 | 3.27(1.05-10.18) |  |  |  |
| Urinary tract infection pseudomonal | 5 | 2.92(1.21-7.06) | Drug clearance | 3 | 314.37(78.62-1257.04) |  |  |  |
| Renal artery stent placement | 5 | 10.52(4.29-25.8) | Bronchial polyp | 3 | 57.16(17.53-186.38) |  |  |  |
| Medial tibial stress syndrome | 5 | 4.21(1.74-10.21) | Skin maceration | 3 | 20.73(6.56-65.47) |  |  |  |
| Lymphoplasmacytoid lymphoma/immunocytoma | 5 | 15.04(6.08-37.24) | Proctectomy | 3 | 7.83(2.51-24.44) |  |  |  |
| Biopsy bone marrow abnormal | 5 | 6.6(2.71-16.08) | Relapsing fever | 3 | 9.07(2.9-28.35) |  |  |  |
| Squamous cell carcinoma of head and neck | 5 | 5.58(2.3-13.57) | Cyanopsia | 3 | 7.25(2.32-22.64) |  |  |  |
| Vaccine induced antibody absent | 5 | 15.26(6.16-37.78) | Sinus polyp | 3 | 4.94(1.59-15.38) |  |  |  |
| Central nervous system fungal infection | 5 | 5.92(2.43-14.39) | Central obesity | 3 | 3.47(1.12-10.8) |  |  |  |
| Vaccination site erythema | 5 | 6.02(2.47-14.63) | Orbital oedema | 3 | 5.91(1.9-18.43) |  |  |  |
| Lymphatic system neoplasm | 5 | 13.21(5.36-32.58) | Eye abscess | 3 | 8.61(2.76-26.91) |  |  |  |
| Wolff-parkinson-white syndrome | 5 | 3.62(1.5-8.77) | Hyperchloraemia | 3 | 3.8(1.22-11.83) |  |  |  |
| Monoclonal immunoglobulin increased | 5 | 4.46(1.84-10.81) | Fungal cystitis | 3 | 34.93(10.92-111.72) |  |  |  |
| Lower respiratory tract infection bacterial | 5 | 2.6(1.08-6.27) | Nuchal rigidity | 3 | 3.36(1.08-10.44) |  |  |  |
| Lymphocyte morphology abnormal | 5 | 3.42(1.41-8.27) | Genital erosion | 3 | 10.42(3.33-32.62) |  |  |  |
| Biopsy bone | 4 | 12.21(4.46-33.42) | Amaurosis | 3 | 3.25(1.05-10.11) |  |  |  |
| Wound sepsis | 4 | 4.21(1.56-11.32) | Dermatophytosis | 3 | 8.61(2.76-26.91) |  |  |  |
| Gingival blister | 4 | 3.01(1.12-8.07) | Dehiscence | 3 | 11.03(3.52-34.54) |  |  |  |
| Aortic rupture | 4 | 3.37(1.26-9.05) | Dermatosis | 3 | 4.42(1.42-13.75) |  |  |  |
| Cystoscopy | 4 | 7.88(2.9-21.36) | Mucosal necrosis | 3 | 15.33(4.88-48.21) |  |  |  |
| Mole excision | 4 | 5.45(2.02-14.7) | Ichthyosis | 3 | 10.2(3.26-31.9) |  |  |  |
| Glaucoma surgery | 4 | 5.55(2.06-14.99) | Burn oesophageal | 3 | 4.98(1.6-15.5) |  |  |  |
| Infestation | 4 | 4.19(1.56-11.26) | Erythromelalgia | 3 | 7.09(2.27-22.13) |  |  |  |
| Nasal herpes | 4 | 4.74(1.76-12.75) | Splenic lesion | 3 | 7.89(2.53-24.64) |  |  |  |
| Vaginal prolapse | 4 | 3.07(1.15-8.24) | Ear malformation | 3 | 8.03(2.57-25.07) |  |  |  |
| Mastoid effusion | 4 | 28.89(10.18-81.99) | Nail ridging | 3 | 4.15(1.33-12.9) |  |  |  |
| Evans syndrome | 4 | 2.71(1.01-7.26) | Arterial rupture | 3 | 3.8(1.22-11.81) |  |  |  |
| Polychromasia | 4 | 4.29(1.59-11.54) | Mucosal toxicity | 3 | 7.37(2.36-23) |  |  |  |
| Elective surgery | 4 | 6.06(2.24-16.37) | Porphyria | 3 | 9.53(3.05-29.79) |  |  |  |
| Onychomalacia | 4 | 9.85(3.62-26.82) | Tongue eruption | 3 | 5.55(1.78-17.29) |  |  |  |
| Synovial rupture | 4 | 3.54(1.32-9.5) | Amyotrophy | 3 | 8.38(2.68-26.19) |  |  |  |
| Spleen atrophy | 4 | 18.05(6.51-50.07) | Haemochromatosis | 3 | 3.21(1.03-9.98) |  |  |  |
| Lung perforation | 4 | 2.88(1.07-7.72) | Cleft lip | 3 | 3.51(1.13-10.93) |  |  |  |
| Eye excision | 4 | 9.32(3.42-25.35) | Solar dermatitis | 3 | 7.96(2.55-24.85) |  |  |  |
| Neurosurgery | 4 | 6.33(2.34-17.09) | Cholangitis infective | 3 | 7.96(2.55-24.85) |  |  |  |
| Meningorrhagia | 4 | 6.52(2.41-17.62) | Laryngotracheal oedema | 3 | 85.74(25.66-286.46) |  |  |  |
| Subdural hygroma | 4 | 3.7(1.38-9.95) | Gallbladder empyema | 3 | 33.09(10.36-105.67) |  |  |  |
| Epiretinal membrane | 4 | 4.21(1.56-11.32) | Respiratory paralysis | 3 | 19.65(6.23-62) |  |  |  |
| Tachycardia paroxysmal | 4 | 5.19(1.92-13.99) | Myeloid leukaemia | 3 | 6.35(2.04-19.8) |  |  |  |
| Urinary bladder polyp | 4 | 4.87(1.81-13.11) | Enterobacter sepsis | 3 | 8.17(2.61-25.5) |  |  |  |
| Pleural infection | 4 | 5.52(2.05-14.89) | Infective corneal ulcer | 3 | 78.59(23.67-261.01) |  |  |  |
| Ureteral stent insertion | 4 | 3.52(1.31-9.46) | Candida test positive | 3 | 7.51(2.41-23.46) |  |  |  |
| White blood cell count | 4 | 4.9(1.82-13.19) | Umbilical discharge | 3 | 25.84(8.14-81.98) |  |  |  |
| Soft tissue haemorrhage | 4 | 3.25(1.21-8.71) | Gallbladder rupture | 3 | 3.31(1.06-10.29) |  |  |  |
| Incision site swelling | 4 | 3.14(1.17-8.43) | Proteus infection | 3 | 4.2(1.35-13.07) |  |  |  |
| Bronchopneumopathy | 4 | 3.83(1.43-10.31) | Intracranial infection | 3 | 19.05(6.04-60.09) |  |  |  |
| Periorbital haemorrhage | 4 | 5.32(1.97-14.34) | Encephalomyelitis | 3 | 9.62(3.08-30.1) |  |  |  |
| Meningitis fungal | 4 | 6.42(2.37-17.35) | Ileostomy closure | 3 | 8.42(2.7-26.31) |  |  |  |
| Urethral obstruction | 4 | 2.95(1.1-7.91) | Vulvovaginal disorder | 3 | 46.01(14.25-148.57) |  |  |  |
| Post procedural oedema | 4 | 5.28(1.96-14.25) | Genital ulceration | 3 | 3.68(1.18-11.44) |  |  |  |
| Carotid endarterectomy | 4 | 7.1(2.62-19.23) | Stoma site candida | 3 | 82.01(24.62-273.14) |  |  |  |
| Disease susceptibility | 4 | 3.32(1.24-8.91) | Epidermolysis bullosa | 3 | 9.38(3-29.34) |  |  |  |
| Haemorrhagic ascites | 4 | 5.22(1.94-14.07) | Secondary hypertension | 3 | 8.86(2.83-27.68) |  |  |  |
| Catheter site haematoma | 4 | 5.38(2-14.52) | Injection site phlebitis | 3 | 18.14(5.76-57.15) |  |  |  |
| Vocal cord operation | 4 | 11.11(4.07-30.35) | Thermal burns of eye | 3 | 3.43(1.1-10.67) |  |  |  |
| Impaired fasting glucose | 4 | 4.71(1.75-12.68) | Globulins decreased | 3 | 7.17(2.3-22.38) |  |  |  |
| Dysplastic naevus | 4 | 3.03(1.13-8.13) | Enzyme inhibition | 3 | 26.57(8.37-84.35) |  |  |  |
| Neurocryptococcosis | 4 | 26.26(9.3-74.12) | Pulmonary vasculitis | 3 | 4.9(1.57-15.26) |  |  |  |
| Globulin abnormal | 4 | 26.26(9.3-74.12) | Leukaemoid reaction | 3 | 10.48(3.35-32.8) |  |  |  |
| Systolic hypertension | 4 | 3.14(1.17-8.43) | Splenic infection fungal | 3 | 46.01(14.25-148.57) |  |  |  |
| Lymph node palpable | 4 | 7.47(2.76-20.24) | Molluscum contagiosum | 3 | 4.99(1.6-15.54) |  |  |  |
| Prostatic obstruction | 4 | 7.34(2.71-19.89) | Chronic hepatic failure | 3 | 4.32(1.39-13.44) |  |  |  |
| Acquired oesophageal web | 4 | 4.87(1.81-13.11) | Drug-disease interaction | 3 | 3.59(1.15-11.18) |  |  |  |
| Urogenital haemorrhage | 4 | 3(1.12-8.04) | Infection protozoal | 3 | 48.36(14.95-156.51) |  |  |  |
| Microsporidia infection | 4 | 3.02(1.13-8.1) | Pre-existing disease | 3 | 7.54(2.42-23.55) |  |  |  |
| Nail bed inflammation | 4 | 3.69(1.37-9.91) | Secondary syphilis | 3 | 26.57(8.37-84.35) |  |  |  |
| Oedematous kidney | 4 | 3.57(1.33-9.58) | Pericardial drainage | 3 | 11.57(3.69-36.25) |  |  |  |
| Pulmonary necrosis | 4 | 4.51(1.68-12.15) | Cholangitis acute | 3 | 3.47(1.12-10.8) |  |  |  |
| Haemorrhagic cyst | 4 | 3.16(1.18-8.49) | Superinfection fungal | 3 | 30.92(9.7-98.55) |  |  |  |
| Serum ferritin abnormal | 4 | 3.7(1.38-9.95) | Lymphocytic leukaemia | 3 | 6.78(2.17-21.17) |  |  |  |
| Periorbital haematoma | 4 | 2.73(1.02-7.31) | Cranial nerve disorder | 3 | 3.96(1.27-12.33) |  |  |  |
| Pneumonia chlamydial | 4 | 10.83(3.97-29.57) | Urticaria chronic | 3 | 4.39(1.41-13.65) |  |  |  |
| Eye infection fungal | 4 | 3.48(1.3-9.35) | Osteogenesis imperfecta | 3 | 5.18(1.66-16.14) |  |  |  |
| Vascular operation | 4 | 3.15(1.17-8.46) | Hepatic candidiasis | 3 | 49.64(15.32-160.8) |  |  |  |
| Pulmonary contusion | 4 | 4.23(1.57-11.37) | Klebsiella test positive | 3 | 4.01(1.29-12.49) |  |  |  |
| Orchitis noninfective | 4 | 6.67(2.46-18.03) | Placental insufficiency | 3 | 3.59(1.15-11.15) |  |  |  |
| Mitral valve replacement | 4 | 3.78(1.41-10.17) | Electrocardiogram j wave | 3 | 44.91(13.92-144.89) |  |  |  |
| Lymphocyte percentage increased | 4 | 4.01(1.49-10.79) | Metastatic lymphoma | 3 | 5.8(1.86-18.09) |  |  |  |
| Fungal abscess central nervous system | 4 | 20.63(7.4-57.54) | Penile haemorrhage | 3 | 3.14(1.01-9.78) |  |  |  |
| Oesophageal food impaction | 4 | 2.87(1.07-7.7) | Ear infection fungal | 3 | 7.76(2.49-24.24) |  |  |  |
| Red cell distribution width abnormal | 4 | 5.94(2.2-16.03) | Pneumonia necrotising | 3 | 4.16(1.34-12.96) |  |  |  |
| B-cell small lymphocytic lymphoma recurrent | 4 | 61.9(20.37-188.05) | Plasma cell leukaemia | 3 | 3.58(1.15-11.13) |  |  |  |
| Immunoglobulins increased | 4 | 2.96(1.1-7.93) | Pyroglutamic acidosis | 3 | 5.6(1.8-17.44) |  |  |  |
| Basosquamous carcinoma of skin | 4 | 20.63(7.4-57.54) | Fibrinous bronchitis | 3 | 36.27(11.33-116.15) |  |  |  |
| Heart valve calcification | 4 | 6.99(2.58-18.91) | Mitochondrial toxicity | 3 | 3.48(1.12-10.82) |  |  |  |
| Chronic respiratory disease | 4 | 3.15(1.17-8.46) | Fat tissue decreased | 3 | 5.58(1.79-17.39) |  |  |  |
| Ocular retrobulbar haemorrhage | 4 | 9.42(3.46-25.63) | Spinal cord haemorrhage | 3 | 6.16(1.98-19.22) |  |  |  |
| Incision site impaired healing | 4 | 2.86(1.07-7.67) | Light chain disease | 3 | 18.68(5.92-58.88) |  |  |  |
| Vessel puncture site bruise | 4 | 5.28(1.96-14.25) | Respiratory moniliasis | 3 | 12.57(4.01-39.43) |  |  |  |
| Ocular lymphoma | 3 | 6.02(1.91-18.95) | Skin neoplasm excision | 3 | 3.25(1.04-10.1) |  |  |  |
| Tongue operation | 3 | 13.83(4.3-44.43) | Intestinal anastomosis | 3 | 4.24(1.36-13.19) |  |  |  |
| Blepharoplasty | 3 | 9.42(2.96-29.93) | Retinal ischaemia | 3 | 5.08(1.63-15.84) |  |  |  |
| Prostatism | 3 | 8.02(2.53-25.4) | Koebner phenomenon | 3 | 28.58(8.99-90.9) |  |  |  |
| Stoma site ulcer | 3 | 4.22(1.35-13.23) | Allergy to metals | 3 | 3.92(1.26-12.2) |  |  |  |
| Malignant polyp | 3 | 3.8(1.21-11.9) | Tertiary adrenal insufficiency | 3 | 58.94(18.05-192.5) |  |  |  |
| Tongue spasm | 3 | 3.25(1.04-10.16) | Electrocardiogram pr shortened | 3 | 13.1(4.18-41.09) |  |  |  |
| Pleural adhesion | 3 | 6.31(2-19.89) | Burkholderia cepacia complex infection | 3 | 8.03(2.57-25.07) |  |  |  |
| Bursa disorder | 3 | 3.53(1.13-11.05) | Acute disseminated encephalomyelitis | 3 | 6.99(2.24-21.8) |  |  |  |
| Kidney rupture | 3 | 4.78(1.52-15) | Activated partial thromboplastin time shortened | 3 | 8.94(2.86-27.94) |  |  |  |
| Biopsy kidney | 3 | 3.28(1.05-10.26) | Vascular access site infection | 3 | 22.73(7.18-71.91) |  |  |  |
| Hepatic rupture | 3 | 5(1.59-15.7) | Salpingo-oophorectomy unilateral | 3 | 21.19(6.71-66.97) |  |  |  |
| Steroid therapy | 3 | 3.76(1.2-11.76) | Blood immunoglobulin a increased | 3 | 5.3(1.7-16.51) |  |  |  |
| Papillitis | 3 | 7.47(2.36-23.62) | Drug chemical incompatibility | 3 | 33.68(10.54-107.61) |  |  |  |
| Inguinal mass | 3 | 3.74(1.19-11.69) | Creatinine renal clearance increased | 3 | 3.85(1.24-11.98) |  |  |  |
| Chest wall mass | 3 | 3.76(1.2-11.76) | Paracoccidioides infection | 3 | 48.36(14.95-156.51) |  |  |  |
| Eyelid bleeding | 3 | 5.6(1.78-17.63) | Central nervous system mass | 3 | 29.02(9.12-92.33) |  |  |  |
| Bronchoscopy | 3 | 3.67(1.17-11.49) | B-cell type acute leukaemia | 3 | 3.41(1.1-10.61) |  |  |  |
| Thoracotomy | 3 | 7.39(2.34-23.34) | Blood creatine phosphokinase mb increased | 3 | 3.74(1.2-11.64) |  |  |  |
| Pericardial disease | 3 | 3.44(1.1-10.76) | Blood bilirubin decreased | 3 | 3.92(1.26-12.2) |  |  |  |
| Arterial stent insertion | 3 | 4.22(1.35-13.23) | Abdominal compartment syndrome | 3 | 4.8(1.54-14.95) |  |  |  |
| Spinal cord haematoma | 3 | 3.27(1.04-10.21) | Vaginal mucosal blistering | 3 | 13.28(4.23-41.68) |  |  |  |
| Gingival operation | 3 | 4.85(1.54-15.23) | Lower respiratory tract infection viral | 3 | 4.56(1.46-14.18) |  |  |  |
| Campylobacter sepsis | 3 | 25(7.57-82.59) | Gastrointestinal wall thinning | 3 | 43.87(13.61-141.4) |  |  |  |
| Tonsillar neoplasm | 3 | 24.07(7.3-79.35) | Human polyomavirus infection | 3 | 4.51(1.45-14.05) |  |  |  |
| Nasal mucosal ulcer | 3 | 8.78(2.77-27.86) | Septic pulmonary embolism | 3 | 12.57(4.01-39.43) |  |  |  |
| Biopsy lymph gland | 3 | 9.42(2.96-29.93) | Liver function test decreased | 3 | 5.89(1.89-18.37) |  |  |  |
| Pneumothorax traumatic | 3 | 4.51(1.44-14.16) | Chronic pulmonary histoplasmosis | 3 | 1886.21(196.19-18134.09) |  |  |  |
| Skin cancer metastatic | 3 | 8.9(2.81-28.25) | Stenotrophomonas test positive | 3 | 21.68(6.86-68.54) |  |  |  |
| Volvulus of small bowel | 3 | 4.89(1.56-15.35) | Urinary tract infection enterococcal | 3 | 3.32(1.07-10.33) |  |  |  |
| Corneal endotheliitis | 3 | 8.23(2.6-26.06) | Acute myeloid leukaemia (in remission) | 3 | 72.55(21.96-239.69) |  |  |  |
| Retroperitoneal mass | 3 | 6.07(1.93-19.13) | Acute motor axonal neuropathy | 3 | 20.73(6.56-65.47) |  |  |  |
| Endovenous ablation | 3 | 12.74(3.98-40.83) | Generalised bullous fixed drug eruption | 3 | 7.99(2.56-24.96) |  |  |  |
| Cancer in remission | 3 | 3.39(1.08-10.59) | Anticonvulsant drug level abnormal | 3 | 23.29(7.36-73.72) |  |  |  |
| Bone marrow necrosis | 3 | 4.71(1.5-14.78) | C-reactive protein decreased | 3 | 3.47(1.12-10.8) |  |  |  |
| Bladder neck obstruction | 3 | 9.15(2.88-29.06) | Chronic graft versus host disease in liver | 3 | 6.05(1.94-18.85) |  |  |  |
| Histiocytic sarcoma | 3 | 11.02(3.45-35.14) | Upper respiratory fungal infection | 3 | 13.19(4.2-41.38) |  |  |  |
| Monocyte count abnormal | 3 | 3.8(1.21-11.9) | Embolic cerebral infarction | 3 | 3.83(1.23-11.93) |  |  |  |
| Splenic artery aneurysm | 3 | 4.92(1.57-15.46) | Negative cardiac inotropic effect | 3 | 94.31(28.02-317.39) |  |  |  |
| Scrotal infection | 3 | 4.45(1.42-13.96) | Retinopathy of prematurity | 3 | 5.44(1.74-16.94) |  |  |  |
| Catheter site cellulitis | 3 | 4.22(1.35-13.23) | Adamts13 activity decreased | 3 | 9.62(3.08-30.1) |  |  |  |
| Breast discolouration | 3 | 6.43(2.04-20.29) | Congenital foot malformation | 3 | 8.13(2.6-25.39) |  |  |  |
| Von willebrand's disease | 3 | 4.11(1.31-12.89) | Ruptured cerebral aneurysm | 3 | 4.73(1.52-14.72) |  |  |  |
| Lymph node haemorrhage | 3 | 26(7.85-86.1) | Suspected drug-induced liver injury | 3 | 16.99(5.4-53.5) |  |  |  |
| Thoracic haemorrhage | 3 | 4.58(1.46-14.36) | Chronic graft versus host disease in lung | 3 | 15.85(5.04-49.85) |  |  |  |
| Ureteric compression | 3 | 9.42(2.96-29.93) |  |  |  |  |  |  |
| Prostatic adenoma | 3 | 5.6(1.78-17.63) |  |  |  |  |  |  |
| Cutaneous lymphoma | 3 | 3.63(1.16-11.36) |  |  |  |  |  |  |
| Lymphadenitis bacterial | 3 | 5.51(1.75-17.32) |  |  |  |  |  |  |
| Bacterial diarrhoea | 3 | 4.19(1.34-13.14) |  |  |  |  |  |  |
| Refractory cancer | 3 | 3.28(1.05-10.26) |  |  |  |  |  |  |
| Rathke's cleft cyst | 3 | 13(4.05-41.67) |  |  |  |  |  |  |
| Calculus prostatic | 3 | 20.97(6.41-68.58) |  |  |  |  |  |  |
| Bifascicular block | 3 | 5.96(1.89-18.78) |  |  |  |  |  |  |
| Prosthesis implantation | 3 | 4.51(1.44-14.16) |  |  |  |  |  |  |
| Papilloma excision | 3 | 16.66(5.15-53.93) |  |  |  |  |  |  |
| Intracardiac mass | 3 | 5.04(1.6-15.83) |  |  |  |  |  |  |
| Viral pericarditis | 3 | 5.86(1.86-18.43) |  |  |  |  |  |  |
| Skin lesion removal | 3 | 8.67(2.73-27.48) |  |  |  |  |  |  |
| Pelvic organ prolapse | 3 | 3.4(1.09-10.64) |  |  |  |  |  |  |
| Basosquamous carcinoma | 3 | 5.91(1.88-18.6) |  |  |  |  |  |  |
| Cellulitis streptococcal | 3 | 6.07(1.93-19.13) |  |  |  |  |  |  |
| Radiotherapy to brain | 3 | 12.74(3.98-40.83) |  |  |  |  |  |  |
| Scrotal haematoma | 3 | 10.16(3.19-32.32) |  |  |  |  |  |  |
| Prolymphocytic leukaemia | 3 | 27.08(8.15-89.93) |  |  |  |  |  |  |
| Oesophageal neoplasm | 3 | 5.33(1.69-16.75) |  |  |  |  |  |  |
| Urinary tract neoplasm | 3 | 14.77(4.59-47.57) |  |  |  |  |  |  |
| Nail bed tenderness | 3 | 8.33(2.63-26.4) |  |  |  |  |  |  |
| Human anaplasmosis | 3 | 10.48(3.29-33.39) |  |  |  |  |  |  |
| Nipple exudate bloody | 3 | 5.86(1.86-18.43) |  |  |  |  |  |  |
| Candida pneumonia | 3 | 3.48(1.11-10.87) |  |  |  |  |  |  |
| Hodgkin's disease nodular sclerosis | 3 | 12.26(3.83-39.24) |  |  |  |  |  |  |
| Platelet function test abnormal | 3 | 7.74(2.45-24.48) |  |  |  |  |  |  |
| Blood alkaline phosphatase | 3 | 7.14(2.26-22.56) |  |  |  |  |  |  |
| Infective exacerbation of bronchiectasis | 3 | 3.39(1.08-10.59) |  |  |  |  |  |  |
| Drug specific antibody absent | 3 | 3.85(1.23-12.04) |  |  |  |  |  |  |
| Capillary permeability increased | 3 | 19.12(5.87-62.24) |  |  |  |  |  |  |
| Aortic intramural haematoma | 3 | 5.8(1.84-18.27) |  |  |  |  |  |  |
| Renal vascular thrombosis | 3 | 4.39(1.4-13.77) |  |  |  |  |  |  |
| Autologous haematopoietic stem cell transplant | 3 | 11.02(3.45-35.14) |  |  |  |  |  |  |
| Limb traumatic amputation | 3 | 3.57(1.14-11.18) |  |  |  |  |  |  |
| Allogenic stem cell transplantation | 3 | 5.24(1.67-16.47) |  |  |  |  |  |  |
| Graft versus host disease oral | 3 | 15.11(4.69-48.72) |  |  |  |  |  |  |
| Post procedural cellulitis | 3 | 5.33(1.69-16.75) |  |  |  |  |  |  |
| Complement deficiency disease | 3 | 27.08(8.15-89.93) |  |  |  |  |  |  |
| Urinary retention postoperative | 3 | 5.37(1.71-16.89) |  |  |  |  |  |  |
| Peripheral nerve operation | 3 | 4.54(1.45-14.26) |  |  |  |  |  |  |
| Ophthalmic vascular thrombosis | 3 | 10.32(3.24-32.85) |  |  |  |  |  |  |
| Hypercalcaemia of malignancy | 3 | 3.51(1.12-10.99) |  |  |  |  |  |  |
| Benign neoplasm of bladder | 3 | 7.14(2.26-22.56) |  |  |  |  |  |  |
| Platelet aggregation abnormal | 3 | 7.22(2.29-22.81) |  |  |  |  |  |  |
| Gastrointestinal arteriovenous malformation | 3 | 3.33(1.07-10.42) |  |  |  |  |  |  |
