# Supplementary material for: Risk stratification of ibrutinib toxicity co-administered with triazole antifungals: insights from real-world pharmacovigilance and clinical evidence
Source: Front Med (Lausanne). 2026 Aug 31;13:1875804. doi: 10.3389/fmed.2026.1875804 (PMC13572604; doi:10.3389/fmed.2026.1875804)
Supplement: Supplementary file 6 [file Table_6.docx]

Table S6 Detection of adverse event signals for the combination of ibrutinib and triazole.

| Drugs | PT | n | ROR(95%Cl) | PRR(χ^2^) |
| --- | --- | --- | --- | --- |
| Ibrutinib+fluconazole | Pneumonia | 31 | 3.24(2.27-4.62) | 3.2(47.05) |
|  | Pyrexia | 22 | 2.37(1.56-3.61) | 2.36(17.26) |
|  | Contusion | 22 | 8.13(5.34-12.39) | 8.05(135.9) |
|  | Platelet count decreased | 20 | 6.75(4.35-10.5) | 6.69(96.91) |
|  | Drug interaction | 17 | 4.1(2.54-6.61) | 4.07(39.44) |
|  | Disease progression | 17 | 5.04(3.13-8.13) | 5(54.54) |
|  | Atrial fibrillation | 16 | 6.08(3.72-9.95) | 6.03(67.28) |
|  | Anaemia | 14 | 2.74(1.62-4.64) | 2.73(15.38) |
|  | Stomatitis | 13 | 7.31(4.23-12.61) | 7.26(70.21) |
|  | Febrile neutropenia | 13 | 7.02(4.07-12.11) | 6.97(66.56) |
|  | Urinary tract infection | 13 | 2.66(1.54-4.59) | 2.65(13.37) |
|  | Haemorrhage | 12 | 4.07(2.31-7.19) | 4.05(27.64) |
|  | Thrombocytopenia | 11 | 3.72(2.06-6.73) | 3.7(21.73) |
|  | Cardiac failure congestive | 11 | 5.73(3.17-10.36) | 5.7(42.66) |
|  | Haemoglobin decreased | 10 | 3.7(1.99-6.9) | 3.69(19.63) |
|  | Epistaxis | 9 | 4.2(2.18-8.09) | 4.18(21.83) |
|  | Lymphadenopathy | 8 | 8.55(4.27-17.13) | 8.52(53.08) |
|  | Pleural effusion | 8 | 5.04(2.52-10.1) | 5.02(25.79) |
|  | Oedema peripheral | 8 | 3.24(1.62-6.49) | 3.23(12.33) |
|  | White blood cell count decreased | 8 | 2.57(1.28-5.14) | 2.56(7.62) |
|  | Sepsis | 7 | 2.32(1.11-4.88) | 2.32(5.26) |
|  | Dysphagia | 7 | 2.69(1.28-5.64) | 2.68(7.38) |
|  | Haematoma | 7 | 10.09(4.8-21.21) | 10.06(57.09) |
|  | Petechiae | 7 | 26.74(12.72-56.19) | 26.63(172.55) |
|  | Cellulitis | 7 | 5.05(2.4-10.6) | 5.03(22.62) |
|  | Gastrointestinal haemorrhage | 7 | 2.91(1.38-6.11) | 2.9(8.72) |
|  | White blood cell count increased | 7 | 7.28(3.46-15.29) | 7.25(37.76) |
|  | Neutrophil count decreased | 7 | 6.1(2.9-12.81) | 6.08(29.7) |
|  | Cataract | 6 | 3.51(1.57-7.82) | 3.5(10.72) |
|  | Fungal infection | 6 | 6.18(2.77-13.77) | 6.16(25.94) |
|  | Pancytopenia | 6 | 4.13(1.85-9.2) | 4.12(14.18) |
|  | Respiratory failure | 6 | 3.25(1.46-7.25) | 3.25(9.34) |
|  | Increased tendency to bruise | 6 | 23.65(10.61-52.74) | 23.58(129.61) |
|  | Chronic lymphocytic leukaemia | 6 | 46(20.62-102.61) | 45.85(262.77) |
|  | Dysuria | 5 | 5.13(2.13-12.34) | 5.12(16.57) |
|  | Ecchymosis | 5 | 30.28(12.58-72.88) | 30.2(141) |
|  | Haematuria | 5 | 5.32(2.21-12.79) | 5.3(17.47) |
|  | Leukocytosis | 5 | 11.22(4.66-26.99) | 11.19(46.37) |
|  | Mantle cell lymphoma | 5 | 113.83(47.23-274.36) | 113.51(555.17) |
|  | Haemorrhage subcutaneous | 5 | 49.95(20.75-120.26) | 49.81(238.71) |
|  | Deep vein thrombosis | 5 | 3.32(1.38-7.98) | 3.31(8.06) |
|  | Subdural haematoma | 5 | 12.44(5.17-29.92) | 12.4(52.41) |
|  | Bone marrow transplant | 5 | 113.33(47.02-273.15) | 113.01(552.71) |
|  | Hip fracture | 4 | 4.55(1.71-12.14) | 4.54(11.05) |
|  | Immunodeficiency | 4 | 8.52(3.19-22.72) | 8.5(26.47) |
|  | Skin cancer | 4 | 5.92(2.22-15.79) | 5.91(16.32) |
|  | Pneumonia fungal | 4 | 30.91(11.58-82.49) | 30.84(115.36) |
|  | Septic shock | 4 | 3.42(1.28-9.12) | 3.41(6.83) |
|  | Skin discolouration | 4 | 3.06(1.15-8.15) | 3.05(5.52) |
|  | Cerebral haemorrhage | 4 | 4.08(1.53-10.89) | 4.08(9.29) |
|  | Urinary incontinence | 4 | 5.22(1.96-13.93) | 5.21(13.63) |
|  | Chronic graft versus host disease | 4 | 41.83(15.67-111.66) | 41.73(158.78) |
|  | Autoimmune haemolytic anaemia | 4 | 35.29(13.22-94.2) | 35.21(132.8) |
|  | Lymphoma | 3 | 6.97(2.25-21.64) | 6.96(15.31) |
|  | Ulcer | 3 | 7.15(2.3-22.19) | 7.14(15.84) |
|  | Pulmonary mass | 3 | 6.33(2.04-19.65) | 6.32(13.44) |
|  | Cryptococcosis | 3 | 64.75(20.83-201.24) | 64.64(187.5) |
|  | Pneumonitis | 3 | 3.79(1.22-11.78) | 3.79(6.16) |
|  | Lip swelling | 3 | 3.31(1.07-10.27) | 3.31(4.83) |
|  | Impaired healing | 3 | 3.46(1.11-10.73) | 3.45(5.23) |
|  | Lymphocytosis | 3 | 53.87(17.34-167.38) | 53.78(155.07) |
|  | Gastric ulcer | 3 | 6.22(2.01-19.32) | 6.22(13.13) |
|  | Staphylococcal infection | 3 | 3.71(1.2-11.53) | 3.71(5.94) |
|  | Candida infection | 3 | 5.39(1.74-16.73) | 5.38(10.7) |
|  | Mental status changes | 3 | 4.68(1.51-14.52) | 4.67(8.66) |
|  | Platelet count abnormal | 3 | 16.03(5.16-49.77) | 16.01(42.18) |
|  | Hypogammaglobulinaemia | 3 | 16.37(5.27-50.81) | 16.34(43.18) |
|  | Supraventricular tachycardia | 3 | 13.16(4.24-40.85) | 13.14(33.62) |
|  | White blood cell count abnormal | 3 | 15.13(4.87-46.97) | 15.1(39.49) |
|  | Full blood count abnormal | 3 | 3.48(1.12-10.81) | 3.48(5.3) |
|  | Graft versus host disease | 3 | 17.1(5.51-53.09) | 17.07(45.37) |
|  | Clostridium difficile infection | 3 | 4.13(1.33-12.82) | 4.12(7.1) |
|  | Blood immunoglobulin m increased | 3 | 188.73(60.56-588.17) | 188.41(555.2) |
|  | Cytokine release syndrome | 3 | 5.3(1.71-16.44) | 5.29(10.43) |
| Ibrutinib+Voriconazole | Pneumonia | 11 | 3.21(1.77-5.82) | 3.17(16.42) |
|  | Drug interaction | 11 | 7.47(4.11-13.56) | 7.35(60.54) |
|  | Aspergillus infection | 10 | 127.62(68.29-238.5) | 125.61(1234.19) |
|  | Atrial fibrillation | 8 | 8.52(4.24-17.12) | 8.43(52.44) |
|  | Bronchopulmonary aspergillosis | 7 | 91.05(43.2-191.86) | 90.04(615.7) |
|  | Thrombocytopenia | 6 | 5.69(2.54-12.71) | 5.64(22.95) |
|  | Disease progression | 6 | 4.97(2.22-11.11) | 4.93(18.84) |
|  | Hypogammaglobulinaemia | 6 | 92.23(41.25-206.18) | 91.36(535.6) |
|  | Epistaxis | 5 | 6.54(2.71-15.76) | 6.49(23.26) |
|  | Oedema peripheral | 5 | 5.68(2.35-13.69) | 5.64(19.11) |
|  | Hip fracture | 4 | 12.76(4.78-34.12) | 12.69(43.09) |
|  | Lymphadenopathy | 4 | 11.96(4.48-31.98) | 11.89(39.93) |
|  | Haemorrhage | 4 | 3.79(1.42-10.13) | 3.77(8.17) |
|  | Dysphagia | 4 | 4.3(1.61-11.49) | 4.28(10.06) |
|  | Pleural effusion | 4 | 7.05(2.64-18.85) | 7.01(20.64) |
|  | Haemoglobin decreased | 4 | 4.14(1.55-11.07) | 4.12(9.47) |
|  | White blood cell count increased | 4 | 11.65(4.36-31.13) | 11.58(38.68) |
|  | Blister | 3 | 5.28(1.7-16.42) | 5.26(10.37) |
|  | Ascites | 3 | 10.73(3.45-33.37) | 10.69(26.35) |
|  | Ecchymosis | 3 | 50.83(16.34-158.1) | 50.59(145.75) |
|  | Cholelithiasis | 3 | 11.66(3.75-36.26) | 11.61(29.1) |
|  | Fungal infection | 3 | 8.64(2.78-26.86) | 8.6(20.16) |
|  | Petechiae | 3 | 32.02(10.3-99.57) | 31.87(89.68) |
|  | Stomatitis | 3 | 4.7(1.51-14.6) | 4.68(8.69) |
|  | Pancytopenia | 3 | 5.78(1.86-17.96) | 5.75(11.79) |
|  | Septic shock | 3 | 7.18(2.31-22.33) | 7.15(15.88) |
|  | Cerebral aspergillosis | 3 | 474.61(152.09-1481.03) | 472.35(1401.9) |
|  | Gastrointestinal haemorrhage | 3 | 3.48(1.12-10.83) | 3.47(5.28) |
|  | Liver function test abnormal | 3 | 14.11(4.54-43.86) | 14.04(36.35) |
|  | Chronic lymphocytic leukaemia | 3 | 64.29(20.67-199.97) | 63.99(185.86) |
|  | Respiratory tract infection | 3 | 11(3.54-34.2) | 10.95(27.14) |
| Ibrutinib+Itraconazole | Pneumonia | 5 | 6.14(2.52-14.97) | 5.97(20.8) |
|  | Pyrexia | 3 | 3.77(1.2-11.84) | 3.72(6) |
|  | Constipation | 3 | 5.71(1.82-17.91) | 5.62(11.42) |
|  | Haemorrhage | 3 | 11.95(3.81-37.47) | 11.73(29.5) |
|  | Sinusitis | 3 | 11.79(3.76-36.97) | 11.58(29.03) |
| Ibrutinib+Isavuconazole | Off label use | 11 | 5.5(2.97-10.2) | 5.12(37.08) |
|  | Febrile neutropenia | 4 | 29.7(10.98-80.35) | 28.82(107.53) |
|  | Platelet count decreased | 4 | 18.5(6.84-50.07) | 17.97(64.21) |
|  | Sepsis | 3 | 13.65(4.34-42.87) | 13.36(34.35) |
|  | Urinary tract infection | 3 | 8.38(2.67-26.32) | 8.21(19.04) |
| Ibrutinib+Posaconazole | Off label use | 23 | 3.12(2.05-4.75) | 3.02(31.52) |
|  | Pneumonia | 8 | 3.16(1.57-6.36) | 3.12(11.6) |
|  | Pyrexia | 7 | 2.87(1.36-6.04) | 2.84(8.37) |
|  | Disease progression | 7 | 7.9(3.74-16.66) | 7.8(41.55) |
|  | Haemorrhage | 6 | 7.76(3.47-17.36) | 7.67(34.85) |
|  | Pleural effusion | 6 | 14.43(6.45-32.29) | 14.26(74.01) |
|  | Muscular weakness | 4 | 4.97(1.86-13.31) | 4.94(12.59) |
|  | Pericardial effusion | 4 | 24.95(9.32-66.76) | 24.74(91.13) |
|  | Hypoxia | 3 | 12.2(3.92-37.98) | 12.13(30.66) |
|  | Myositis | 3 | 50.72(16.29-157.88) | 50.4(145.19) |
|  | Full blood count abnormal | 3 | 13.24(4.26-41.22) | 13.16(33.73) |
|  | Graft versus host disease | 3 | 65.03(20.89-202.45) | 64.62(187.79) |
|  | Gastrointestinal disorder | 3 | 3.17(1.02-9.86) | 3.15(4.42) |
