# Supplementary material for: Risk stratification of ibrutinib toxicity co-administered with triazole antifungals: insights from real-world pharmacovigilance and clinical evidence
Source: Front Med (Lausanne). 2026 Aug 31;13:1875804. doi: 10.3389/fmed.2026.1875804 (PMC13572604; doi:10.3389/fmed.2026.1875804)
Supplement: Supplementary file 7 [file Table_7.docx]

Table S7 The comparison of demographic and clinical characteristics between included and excluded cases in the combination therapy group (n=590)

| Characteristics | Included cases (n=157, 26.61%) | Excluded cases (n=433, 73.39%) |
| --- | --- | --- |
| Male | 95 (60.51%) | 273 (63.05%) |
| 18 year≤ Age ≤64 year | 51(32.48%) | 112(25.87%) |
| Age >64 year | 90 (57.32%) | 142 (32.79%) |
| Serious outcome | 130 (82.80%) | 265 (61.20%) |
